# Exploration of Human Skin Phageome to Reveal Endolysins and Novel Antimicrobial Peptides for Therapeutic Applications

**DOI:** 10.1002/mbo3.70115

**Published:** 2025-11-09

**Authors:** Jibon Kumar Paul, Arzuba Akter, Nurnabi Azad Jewel, Mohimenul Haque Rolin, Daniyal Karim, Raphael Kabir Niloy, Shakhinur Islam Mondal

**Affiliations:** ^1^ Department of Biochemistry and Molecular Biology Shahjalal University of Science and Technology Sylhet Bangladesh; ^2^ Department of Genetic Engineering and Biotechnology Shahjalal University of Science and Technology Sylhet Bangladesh

**Keywords:** antimicrobial peptides, antimicrobial resistance, endolysins, human skin phageome

## Abstract

The global rise of antibiotic‐resistant pathogens has intensified the search for alternative therapeutics. Bacteriophage‐derived endolysins are emerging as promising candidates. They exhibit strong potential due to their target specificity, rapid bactericidal action, and low tendency to induce bacterial resistance. This study presents a comprehensive metagenomic analysis of the human skin phageome using 1564 samples from 10 metagenomic projects. Our analysis led to the classification of 696 phage genomes into clusters and singletons. These genomes displayed considerable variation in size, GC content (average 56%), and coding efficiency (72%). A total of 968 endolysins were identified, including 75 SAR variants, with diverse domain architectures such as CHAP, Amidase, and SH3, suggesting host‐specific adaptations. Notably, we identified 37 previously unreported endolysin‐derived antimicrobial peptides (AMPs), several of which exhibited nontoxic, antifungal, and antiviral properties. Molecular dynamics and docking studies revealed strong binding affinity and stability of peptides EP‐464 and EP‐519 to key virulence factors, including *Staphylococcus epidermidis* autolysin (PDB: 4EPC), beta‐lactamase VIM‐2 (PDB: 5O7N), and AHL synthase LasI (PDB: 1RO5). These interactions suggest potential for disrupting bacterial virulence, resistance mechanisms, and quorum sensing. This study provides the first large‐scale functional characterization of the human skin phageome focused on therapeutic endolysins and their novel AMP derivatives, offering promising candidates for the development of next‐generation antimicrobial agents. However, further experimental validation is essential to assess their clinical efficacy in treating skin‐related infections.

## Graphical abstract

This study uncovers diverse endolysins and novel antimicrobial peptides from the human skin phageome, highlighting their potential to target antibiotic‐resistant pathogens and disrupt virulence mechanisms, paving the way for next‐generation skin therapeutics.

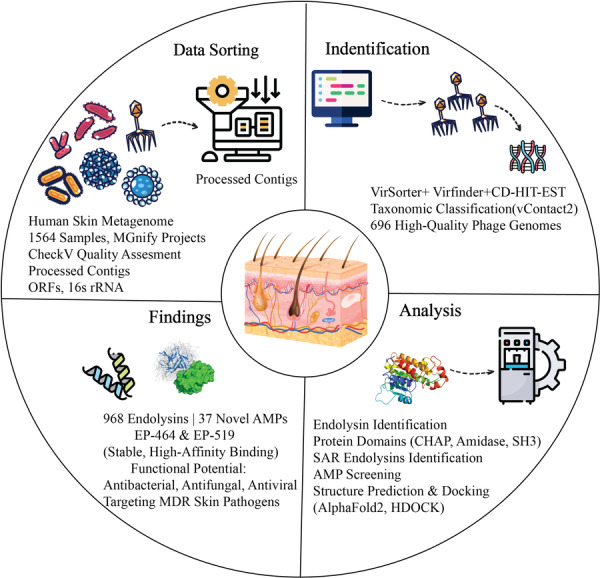

## 1 Introduction

The discovery of antibiotics in the early 20th century revolutionized medicine, saving millions of lives and contributing to an average increase of 23 years in human lifespan (Hutchings et al. 2019). Antibiotics are affordable, effective, and essential in preventing infections during surgical procedures and cancer therapies. However, the widespread and often unregulated use of antibiotics has led to a rapid rise in antibiotic resistance, posing a serious global health threat (Rahman et al. 2021). The World Health Organization (WHO) warned in its 2019 report that antimicrobial resistance (AMR) already causes approximately 700,000 deaths annually and could result in 10 million deaths per year by 2050, with an estimated 24 million people falling into extreme poverty by 2030 if no action is taken.

Despite the escalating crisis, the development of new antibiotics has significantly slowed over recent decades (Hutchings et al. 2019). This has spurred the search for alternative antimicrobial strategies, among which bacteriophage (phage) therapy has re‐emerged as a promising approach. Phages are viruses that specifically infect and lyse bacteria, primarily using enzymes such as endolysins that degrade bacterial cell walls during the final stage of infection (Briers 2014). Historically, phages were used to treat infections like cholera and dysentery in Europe and the former USSR over a century ago (Irshad et al. 2012), but the advent of antibiotics limited their development.

Compared to conventional antibiotics, phages and their endolysins offer several advantages (Mondal et al. 2020). They exhibit high specificity toward target pathogens while sparing beneficial commensal microbes (Mondal et al. 2020, 2021; Rahman et al. 2021), can be effective in biofilms and mucosal environments, and present a lower risk of resistance development (H. Wang et al. 2016). Endolysins, in particular, can function externally on bacterial cells and do not require viable bacterial cultures for activity, making them suitable for both systemic and topical applications. Furthermore, advances in protein engineering have enabled the development of synthetic or chimeric endolysins with improved host specificity and lytic efficacy (Briers 2014; Haddad Kashani et al. 2018; T. Khan, Mondal, et al. 2024).

In addition to intact endolysins, antimicrobial peptides (AMPs), particularly those derived from endolysins, are emerging as promising alternatives to traditional antibiotics due to their broad‐spectrum activity, rapid mode of action, and reduced potential for resistance development (Thandar et al. 2016). Endolysin‐derived AMPs often retain enzymatic or membrane‐disruptive functions and can be bioengineered for enhanced specificity, stability and therapeutic potential (Wojciechowska 2025). These low molecular‐weight oligopeptides, typically 15–150 amino acids in length, disrupt bacterial membranes and exhibit broad‐spectrum antimicrobial activity (Vos et al. 2017). In the context of the skin microbiome, such peptides hold promise as targeted therapeutics against multidrug‐resistant (MDR) pathogens and biofilm‐associated infections, especially in topical applications. Additionally, their nontoxic nature and potential antiviral and antifungal properties further underscore their value as next‐generation antimicrobials for treating skin‐related infections (Schmelcher et al. 2012; Nelson et al. 2001; Haddad Kashani et al. 2018).

One example of a clinically approved endolysin‐based product is Staphefekt SA.100, which is used in Europe to treat *Staphylococcus aureus* skin infections, including methicillin‐resistant strains (Totté et al. 2017). Skin and soft tissue infections (SSTIs) remain a significant burden worldwide, with an estimated 155 million cases in 2013 alone (Vos et al. 2017). Infections such as cellulitis, impetigo, erysipelas, and folliculitis are predominantly caused by pathogens including *Staphylococcus aureus*, *Streptococcus pyogenes*, and *Pseudomonas aeruginosa* (Vary and O'Connor 2014; AlSalem et al. 2015). In Bangladesh, studies have reported high rates of AMR in skin infections, with methicillin‐resistant *S. aureus* found in up to 31% of patients (Kawaguchiya et al. 2011). Despite the existence of the National Strategic Plan for AMR Containment (2021–2026), more research is needed into alternative treatments to counteract the rise in drug‐resistant bacteria (Ahmed et al. 2014).

The human skin microbiome, a diverse ecosystem of bacteria, fungi, viruses, and archaea, plays a critical role in skin health and defending against infections (Yang et al. 2015). While most skin microbiome studies have focused on bacterial communities, bacteriophages, especially those infecting dominant genera like *Staphylococcus*, *Propionibacterium*, and *Corynebacterium*, remain underexplored. These phages regulate microbial balance and may harbor novel therapeutic elements such as endolysins and AMPs.

Advances in metagenomics and computational biology now allow in‐depth investigation of the human skin virome, including uncultured phage genomes. Through whole‐genome shotgun sequencing, it is possible to mine the skin metagenome for phage‐encoded endolysins and AMPs, enabling the discovery of novel antimicrobial candidates. Computational pipelines can rapidly annotate gene functions, predict antimicrobial activity, and model protein structures and host–pathogen interactions (Fancello et al. 2020; Karim et al. 2024), thereby accelerating discovery and reducing laboratory costs in the early phases of development.

Compared to well‐studied niches like the gut or oral microbiome, the skin harbors a distinct phage population that can uniquely shape colonization by antibiotic‐resistant bacteria. Exploring the skin phageome provides a valuable opportunity to uncover new bioactive compounds tailored for the cutaneous environment, targets that may be missed by studies on gut‐ or oral‐derived phages (Ellis et al. 2019).

This study aims to comprehensively analyze endolysins and their derivative AMPs from the human skin metagenome, focusing on their spatial distribution, structural features, evolutionary relationships, and therapeutic potential. By integrating metagenomic and computational approaches, we aim to uncover novel antimicrobial molecules that may be effective against MDR pathogens, particularly those responsible for skin infections. This study fills a major gap in skin phageome research and provides a foundation for future development of targeted, phage‐derived therapeutics that address the limitations of conventional antibiotics, such as biofilm resistance, off‐target effects, and the rising threat of AMR.

## 2 Methods

### 2.1 Skin Metagenome Sequence Selection

To elucidate the assembly sequence of the human skin microbiome, the EMBL‐EBI MGnify database (https://www.ebi.ac.uk/metagenomics) was employed as a pivotal resource. A comprehensive investigation was initiated by utilizing the search term “human skin microbiome” to filter “Processed Contigs” datasets within the database. Particular emphasis was placed on selecting samples derived from human hosts to ensure the biological relevance of the data. Following this, a meticulous collection of all associated European Nucleotide Archive (ENA) files linked to the human skin metagenome was performed.

### 2.2 Metagenome‐Assembled Viral Genome

To predict metagenome‐assembled viral genomes CheckV (Nayfach et al. 2021) was used, which was compiled by removing host contamination, estimating genome completeness, predicting closed genomes (using DTRs and ITRs), and summarizing quality into five tiers (complete, high‐, medium‐, low‐quality, or undetermined) based on MIUViG standards.

### 2.3 Genome Sequence and Annotation

The genomes were comprehensively annotated to identify various genetic features, including coding DNA sequences (CDS), tRNAs, tmRNAs, CRISPR arrays, virulence factors, toxins, and antimicrobial resistance genes detected with Pharokka v1 (Bouras et al. 2023). For the prediction of CDS within the nonredundant phage genomes, Prodigal, integrated within Pharokka version 1.3.2, was employed in its meta mode to ensure accurate and efficient analysis.

### 2.4 Genome Clustering and Taxonomy

Proteins from all genomes were classified into phage protein families or phams using PhaMMseqs, with parameters set to 35% amino acid identity and 80% coverage, and the pangenome option (‐p) enabled. Subsequently, phages were clustered based on shared phams using PhamClust, applying a clustering threshold of 0.3. (Gauthier and Hatfull 2023). ViPTree (Nishimura et al. 2017) was utilized to construct a proteomic tree of viral sequences based on genome‐wide sequence similarities, incorporating all current members of the queries as well as genomes related to the queries.

### 2.5 Extraction of Viral Contigs

The contigs derived from the viral fraction were rigorously screened using two gene enrichment‐based methodologies: VirSorter 2 version 2.2.4 (Guo 2021) and VirFinder v1.1 (Ren et al. 2017). These approaches were employed to identify and exclude contigs exhibiting similarities to bacterial, archaeal, and fungal genomes. VirSorter was utilized to extract viral contigs from both the RefSeqABVir (using the parameter –db 1) and Viromes (using the parameter –db 2) databases. To ensure optimal accuracy, the “virome decontamination” mode (with the parameter –virome 1) was applied. In parallel, VirFinder analysis was conducted with the default prediction model, applying a significance threshold of *p* < 0.05 to enhance the detection specificity. Only viral contigs identified by one or both methodologies were retained for subsequent analyses. Residual contigs, following this dual‐screening process, were confidently classified as viral and proceeded to further analysis. During the contig pooling and redundancy elimination stages, the identified viral contigs were subjected to CD‐HIT‐EST (Fu et al. 2012) to reduce redundancy and streamline downstream analyses.

### 2.6 Identifying Protein‐Like Sequence

The viral contigs identified by VirSorter2 and VirFinder were further analyzed using MetaProdigal (Hyatt et al. 2012), a tool designed for gene prediction in metagenomic data. These nucleotide sequences were then translated into protein sequences. To remove redundancy, the resulting protein sequences were clustered using CD‐HIT.

### 2.7 Probable Endolysin: Sequences Identification and Characterization

For the identification and characterization of probable endolysin sequences, a reference data set compiled by Fernández‐Ruiz et al. (2018) was utilized, encompassing a wide range of endolysin sequences from various domains. Protein‐like sequences derived from the viral contigs were compared to this reference data set using *blastp*, implemented through DIAMOND (Buchfink et al. 2021), a tool optimized for high‐speed, high‐precision sequence comparisons. Putative endolysins were identified based on stringent criteria, including an e‐value threshold of ≤ 0.00001(1e^−05^), query coverage of ≥ 30%, sequence identity of ≥ 50%, and an alignment length exceeding 50 amino acids.

Further characterization of the identified sequences included the prediction of protein solubility and subcellular localization using the SOSUI tool (https://bio.tools/sosui) (Hirokawa et al. 1998), with a focus on distinguishing between soluble and membrane proteins, including single‐spanning membrane proteins. The isoelectric point (pI) and molecular weight (Mw) of the proteins were calculated using IPC2.0 (https://ipc2.mimuw.edu.pl/) (Kozlowski 2021), while the Grand Average of Hydropathy (GRAVY) Index and the Instability Index versus Aliphatic Index were determined using the ProtParam tool (https://web.expasy.org/protparam/) (Gasteiger 2003). Domain architecture of the potential endolysins was analyzed using HMMER (https://hmmer.org/) (Finn et al. 2011), querying the Pfam database (http://pfam.xfam.org/) and the NCBI Conserved Domain Database (CDD) (https://www.ncbi.nlm.nih.gov/Structure/cdd/wrpsb.cgi) (Marchler‐Bauer et al. 2015). Signal peptide prediction was carried out using a consensus‐based approach involving multiple computational tools. Specifically, SignalP6.0 (Teufel et al. 2022), Phobius (Kall et al. 2007), and TOPCONS (Tsirigos et al. 2015), were employed to assess the presence of signal peptides and transmembrane domains in the endolysin protein sequences. Multiple sequence alignments were generated using MEGA11 software (Tamura et al. 2021), and the resulting alignments were utilized for constructing a phylogenetic tree. Visualization of the phylogenetic tree was achieved through the Interactive Tree of Life (iTOL) platform (https://itol.embl.de/) (Letunic and Bork 2021), facilitating detailed analysis of evolutionary relationships between the identified endolysin sequences.

### 2.8 Signal–Arrest–Release (SAR) Endolysin Identification

Signal–arrest–release (SAR) endolysins are a class of phage‐encoded lytic enzymes that utilize N‐terminal signal peptides to initiate membrane translocation. These endolysins remain anchored in the membrane and are released in a regulated manner to degrade the peptidoglycan layer during phage‐induced lysis (Xu et al. 2004). The SOSUI, TMHMM, Phobius, and Octopus databases were utilized to detect transmembrane domains located at the N terminus of all potential endolysins that were found. The software identifies endolysins that possess a transmembrane domain and exhibit a significant proportion of Gly/Ala (40%–60%). Additionally, these endolysins are annotated as SAR endolysins and contain 0 to 2 basic residues (Bai et al. 2020).

### 2.9 Endolysins Host Prediction and Orthologous Group Identification

Host prediction was conducted using the jackhmmer program within the HMMER software suite to search for endolysin sequences in the PhaLP database (Criel et al. 2021). Additionally, the UniProt BLAST program (https://www.uniprot.org/blast) was used to identify potential host organisms by analyzing the endolysin sequences based on the highest bit scores and lowest *E* values, along with identifying orthologous groups (OGs) through mmSeq. 2 (Hernández‐Salmerón and Moreno‐Hagelsieb 2020).

### 2.10 Taxonomic Classification of Viral Contigs

Taxonomic classification of the viral contigs was conducted using vConTACT v.2.0 (Jang et al. 2019), a network‐based tool designed to improve the initial categorization of viral sequences using “ProkaryoticViralRefSeq. 211‐Merged” as a reference database. This tool utilizes whole‐genome gene‐sharing networks to classify viruses by integrating gene content similarity with hierarchical clustering algorithms. vConTACT v.2.0 offers a robust framework for predicting viral taxonomy by grouping contigs based on shared gene content, supported by confidence scores to enhance the reliability of taxonomic predictions. A “genome_by_genome_overview. csv,” which contains clustering and taxonomic information, and “c1.ntw,” useful for visualizing genome relationships in tools like Cytoscape (Paul Shannon 1971).

### 2.11 Identification and Characterization of Antimicrobial Peptides

A sliding window approach was used to fragment each endolysin into peptides, applied iteratively over the entire amino acid sequence. The window size was set at 20 amino acids, shifting by one amino acid with each step. The resulting peptides were then screened for antimicrobial properties using AMP prediction tools, including AxPEP, AmpGram, CAMPR4, and Antimicrobial Peptide Scanner version 2 (Veltri et al. 2018; Burdukiewicz et al. 2020; Yan et al. 2020; Gawde et al. 2023). Peptides predicted to possess antimicrobial properties by all the tools were selected for further analysis. Antifp (Agrawal et al. 2018) and iAMPred (Meher et al. 2017) were employed to predict their antifungal properties and iAMPred was utilized to predict antiviral activity. Toxin Pred (Rathore et al. 2024) was employed to assess the toxicity of the selected AMPs.

### 2.12 Structural Modeling and Stability Assessment of Peptides

The 3D structure of the putative antimicrobial peptides (AMPs) was generated using AlphaFold2 (Jumper et al. 2021). To evaluate the structural stability of the AMPs, molecular dynamics (MD) simulations were conducted using GROMACS (version 2023.3) with the CHARMM36 all‐atom force field (Abraham et al. 2015). Each AMP was solvated using the CHARMM‐modified TIP3P water model, with Na/Cl ions added to neutralize the system. Energy minimization was performed using the steepest descent algorithm over 50,000 steps. Subsequently, a two‐phase equilibration process was carried out. The simulation process began with an initial phase using the NVT ensemble, in which the number of particles, volume, and temperature were held constant. This was followed by a second phase using the NPT ensemble, where the number of particles, pressure, and temperature were maintained for 100 picoseconds (equivalent to 50,000 steps) utilizing the Particle Mesh Ewald (PME) method. After these equilibration phases, MD simulations were conducted for each AMP over a duration of 100 ns. Root mean square deviation (RMSD) values were calculated from the resulting simulation trajectories using the gmx rms tool available in GROMACS.

### 2.13 Molecular Docking Analysis

Based on the lowest average RMSD values, the top antimicrobial peptides (AMPs) were chosen for molecular docking analysis. To explore the binding interactions between these predicted AMPs and were then docked with several key targets, including the *Staphylococcus epidermidis* virulence factor autolysin (PDB: 4EPC) (Zoll et al. 2012), Beta‐lactamase VIM‐2 in complex with (2 R)‐1‐(2‐Benzyl‐3‐mercaptopropanoyl)piperidine‐2‐carboxylic acid (PDB: 5O7N) (Büttner et al. 2018), and the crystal structure of AHL Synthase LasI (PDB: 1RO5) (Gould et al. 2004), which was selected as the specific protein target. Potential binding sites on the target protein were predicted using the CASTp server. Following this, molecular docking of the seven most stable AMPs with the selected protein target was performed using the HDOCK (http://hdock.phys.hust.edu.cn/) server.

### 2.14 Molecular Dynamics Simulation

MD simulations were carried out for 100 ns to evaluate the autolysin–AMP complexes, selected based on their HDOCK scores. The analysis involved using built‐in GROMACS programs to assess parameters such as RMSD, RMSF, radius of gyration (Rg), and solvent‐accessible surface area (SASA) from the MD simulation trajectories.Graphical representations of the resulting analyses were created using xmgrace.

## 3 Results

### 3.1 Data Retrieval

The EMBL‐EBI MGnify database was utilized to obtain metagenomic data for the assembly of the human skin microbiome. Processed contigs were selected for further analysis (Table 1). The data set comprised 1564 human skin microbiome samples obtained from 10 MGnify projects. Across these, a total of 3122 distinct analyses were conducted, as some projects included multiple analysis runs per sample. The retrieved metagenomic data served as the foundation for subsequent analyses aimed at elucidating the complex genomic structure of the human skin microbiome.

**Table 1:** Overview of metagenomic data sets retrieved from the MGnify database for human skin microbiome.

Mgnify_ID	ENA_Accession	Biome	Samples	Analyses
MGYS00005386	PRJEB33666	Skin	175	175
MGYS00005109	PRJEB33912	Skin	16	16
MGYS00006009	PRJEB51311	Skin	12	12
MGYS00005090	PRJEB33173	Naris	120	120
MGYS00005034	PRJEB29894	Skin	33	66
MGYS00005423	PRJEB32759	Skin	47	47
MGYS00005383	PRJEB33718	Skin	504	504
MGYS00005573	PRJEB39283	Skin	143	143
MGYS00005379	PRJEB33701	Skin	420	1884
MGYS00003598	PRJEB25958	Skin	94	188

### 3.2 Viral Sequence Identification

The assembled metagenomic contigs were subjected to simultaneous analysis using VirSorter and VirFinder to identify virus‐like sequences. This combined approach resulted in the detection of 1,669,231 viral sequences. To ensure data quality and eliminate redundancy, the CD‐HIT‐EST method was employed, reducing the data set to 853,972 nonredundant viral sequences. This refinement process provided a robust and streamlined data set, facilitating more accurate and comprehensive subsequent analyses of the viral content within the human skin microbiome.

### 3.3 Genomic Insights

**Figure 1:**
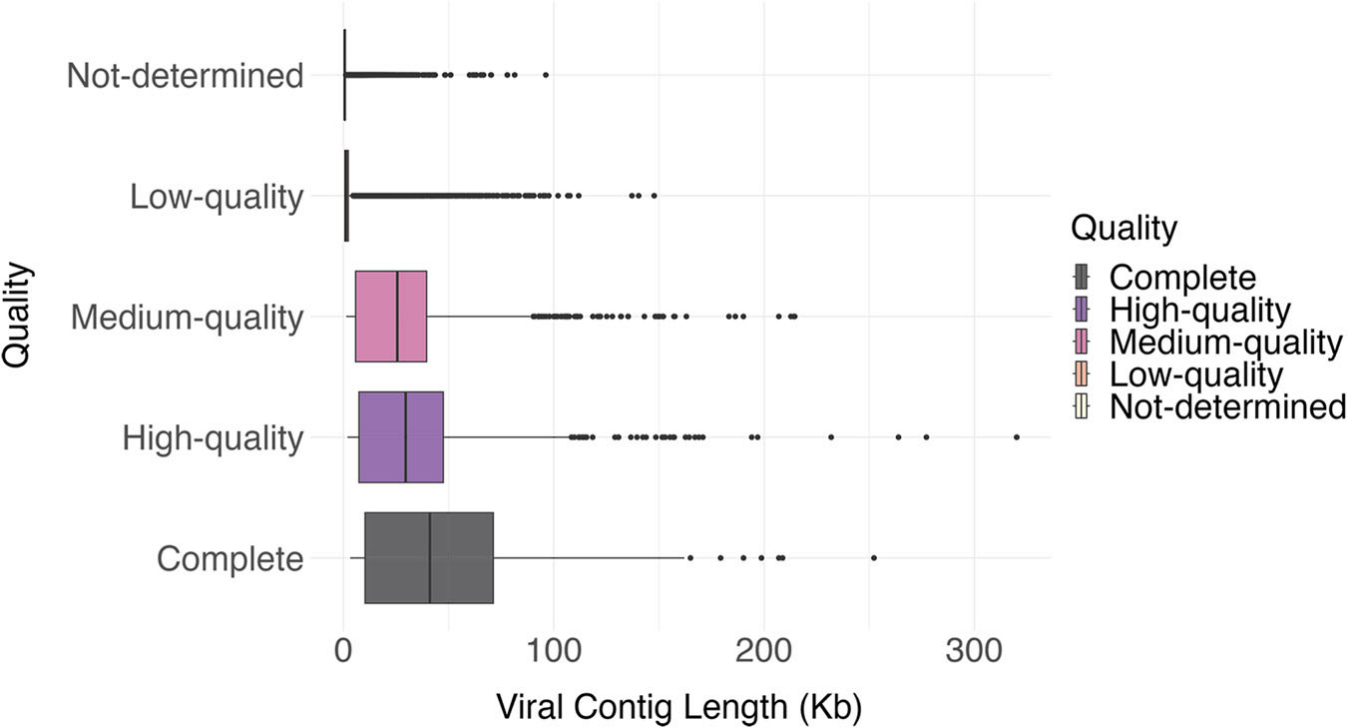
Distribution of genomes across various quality categories: “Not‐determined,” “Low‐quality,” “Medium‐quality,” “High‐quality,” and “Complete.” The *x*‐axis represents the number of genomes, while the *y*‐axis categorizes their quality. No genomes are categorized as “Not‐determined,” and “Low‐quality” genomes are either minimally present or absent, indicating a robust data set. The “Medium‐quality” category has the highest representation, closely followed by the “High‐quality” category, signifying a strong data set for reliable analysis. A smaller but significant subset of genomes is classified as “Complete,” representing the highest‐quality genomes. This distribution reflects the data set's overall reliability and emphasizes the high prevalence of usable and high‐quality genomic data.

The metagenome‐assembled viral genomes (MAVGs) were processed using CheckV with miuvig quality assessment, yielding a high‐quality data set (Figure 1). Among these, 299 genomes were identified as 100% complete with no genome fragments, while an additional 638 genomes were classified as high quality (> 90% completeness). After excluding genomes with contamination levels exceeding 5%, those lacking viral genes, and identified proviruses, the final set of high‐quality (> 90%) and complete genomes increased to 696. A detailed summary of genome quality categories for all viral sequences, including low‐ and medium‐quality groups, is presented (Table S1).

**Figure 2:**
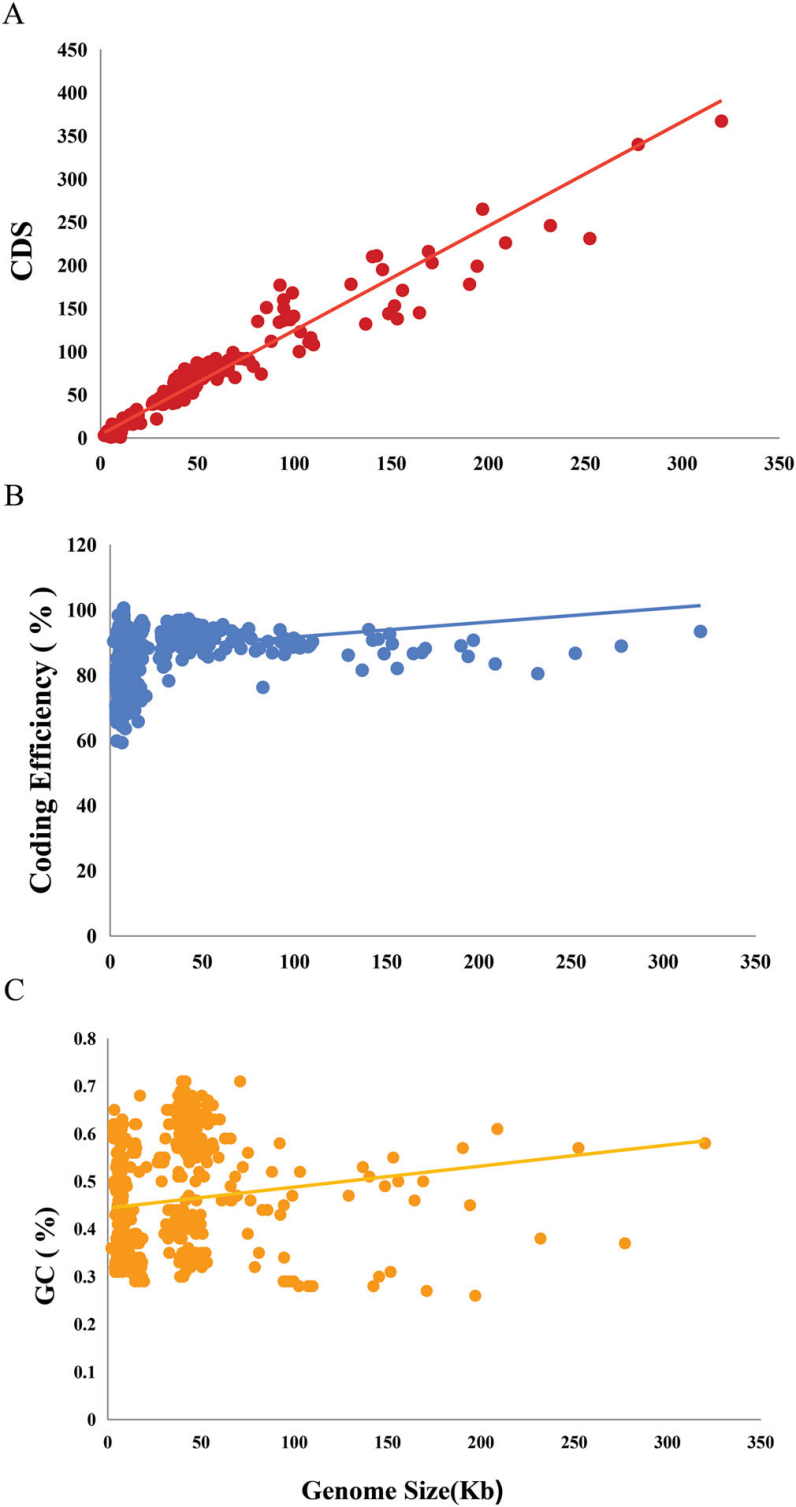
The correlation between phage genome size (*x*‐axis) and GC content, coding efficiency, and the number of coding sequences (CDS) (each represented on the *y*‐axis) was examined. These results highlight the genomic adaptability and functional complexity of the organisms analyzed. The correlation between (A) CDS number, (B) coding efficiency, and (C) GC content suggests that the genomes are structured for optimal protein production, which may enhance the organisms' ability to thrive in diverse ecological environments.

The analysis of 696 high‐quality genomes revealed key genomic characteristics that offer insights into the structural and functional attributes of the organisms. The average GC content across the genomes was 56%. Additionally, the coding efficiency of the genomes averaged 72%, signifying that a substantial portion of the genomic sequence is devoted to protein‐coding regions (Figure 2). This high coding efficiency reflects the organisms' reliance on functional genes for metabolic and cellular processes.

### 3.4 Cluster Assignment and Taxonomic Classification of Phages

To determine the taxonomy of 696 human skin phage genomes, clustering was conducted using protein sharing as the primary criterion. PhamClust calculated the Proteomic Equivalence Quotient (PEQ) between pairs of phages based on shared gene identity, with PEQ values ranging from 0% (indicating no shared genes) to 100% (indicating complete gene identity). This analysis resulted in the identification of 81 distinct clusters (Clusters 1–81) and 172 singletons (Figure S1).

**Figure 3:**
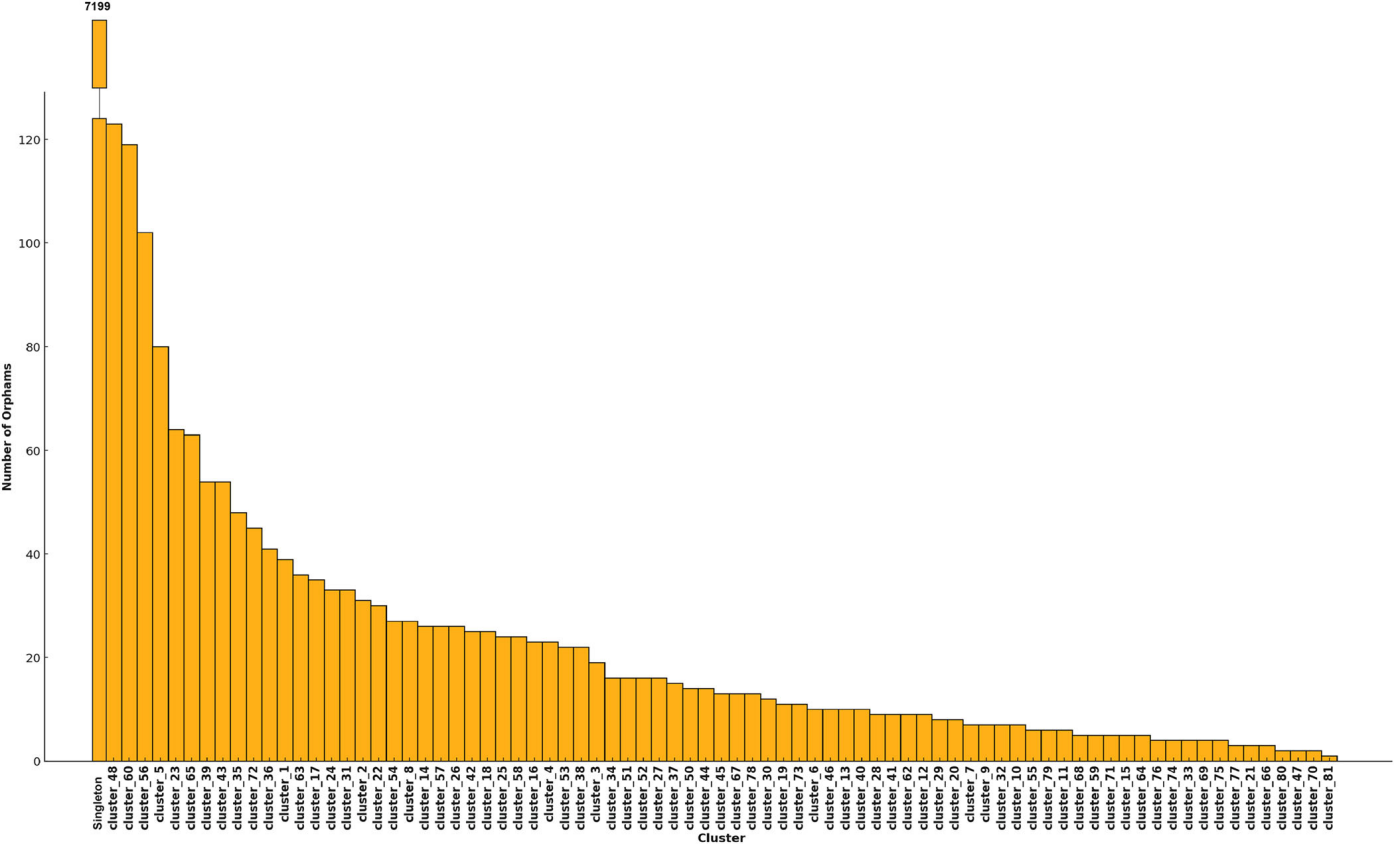
A bar plot illustrating the number of orphams per cluster. The clusters are represented along the *x*‐axis and are arranged in ascending order based on the number of orphams, which is shown on the *y*‐axis.

Orphams, genes that lack detectable homologs in current databases, represent unique, potentially novel functional elements of the virome. These sequences are often indicative of unexplored viral diversity and may encode lineage‐specific adaptations. These proteins, predominantly associated with singleton phages, represent a distinct dimension of viral diversity, often linked to functions that remain largely unexplored or undefined. In total, this study identified 7199 orphams within the singleton phages, which are unique and isolated viral genomes that do not belong to any cluster of genetically similar genomes. Additionally, 1748 orphams were detected across the 81 defined clusters (Figure 3). The presence of orphams, particularly in genetically distinct singleton phages that lack close relatives in current genomic databases, underscores their significant role in driving viral genetic innovation.

**Figure 4:**
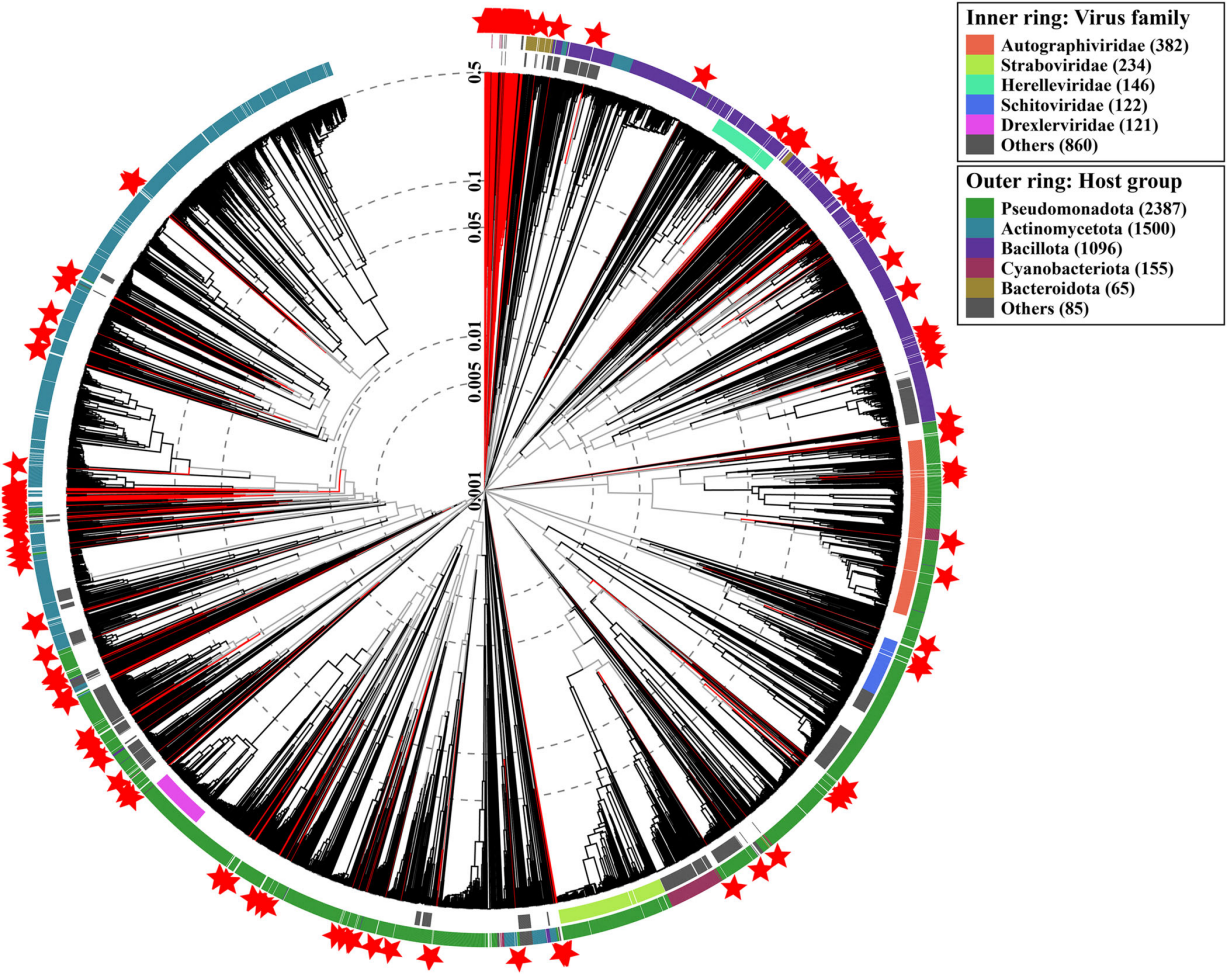
Circular clustered tree representing the evolutionary relationships among 253 viral genomes, constructed using VipTree based on pairwise tBLASTx scores. The tree incorporates all current reference members, with branch lengths reflecting genomic similarity scores (SG). Distinct clusters correspond to host‐specific subsets, including prokaryotic and eukaryotic viruses, offering insights into their evolutionary relationships. The color coding represents different viral categories or clusters, enhancing the visualization of phylogenetic diversity.

A circular clustered tree was constructed using VipTree, leveraging pairwise tBLASTx scores processed through VipTreeGen to analyze 253 genomes alongside current reference members. This approach generates “reference trees” based on all‐against‐all genomic similarity scores (SG) derived from tBLASTx alignments. Additionally, VipTree provides tailored proteomic trees for host‐specific subsets, such as eukaryotic and prokaryotic categories, offering a detailed view of viral evolutionary relationships across diverse hosts (Figure 4).

The analysis, conducted using ViPTree version 4.0, focused on double‐stranded DNA (dsDNA) sequences from prokaryotic hosts. Gene prediction was performed using Prodigal with Genetic Code 11, corresponding to the Bacterial, Archaeal, and Plant Plastid Code, providing valuable insights into the genomic architecture and evolutionary dynamics of the analyzed sequences.

At the host group and family levels, taxonomic assignment of human skin microbiome phages was carried out using the ICTV classification system. A total of 37 host groups were identified, including a diverse range of bacterial genera such as *Acinetobacter*, *Aeromonas*, *Bacillus*, *Corynebacterium*, *Pseudomonas*, *Staphylococcus*, *Vibrio*, *Xanthomonas*, and *Xylella* (Figure S2). However, despite the identification of these host groups, many phages remain unassigned at the family level. Only 8 viral families were confidently recognized, including *Autographiviridae*, *Herelleviridae*, *Peduoviridae*, *Rountreeviridae*, *Saffermanviridae*, *Schitoviridae*, and *Zierdtviridae*. However, a significant portion of the phages still lacks classification within these families, highlighting the need for further refinement in taxonomic resolution to better understand the diversity of phages associated with the human skin microbiome.

### 3.5 Protein‐Like Sequence Identification and Putative Endolysin Sequences Identification

The protein sequences were obtained through MetaProdigal. A comprehensive collection comprising 1,363,756 protein sequences was identified from the viral contigs. Subsequently, redundancy was mitigated utilizing CD‐HIT, resulting in a refined set of 1,010,616 proteins selected for further investigation. Diamond was then employed to identify 968 endolysin sequences, utilizing an extensive reference data set encompassing a diverse array of endolysins. This systematic approach ensured the acquisition of a robust data set conducive to subsequent in‐depth analysis and interpretation.

### 3.6 Endolysin Characterization

**Figure 5:**
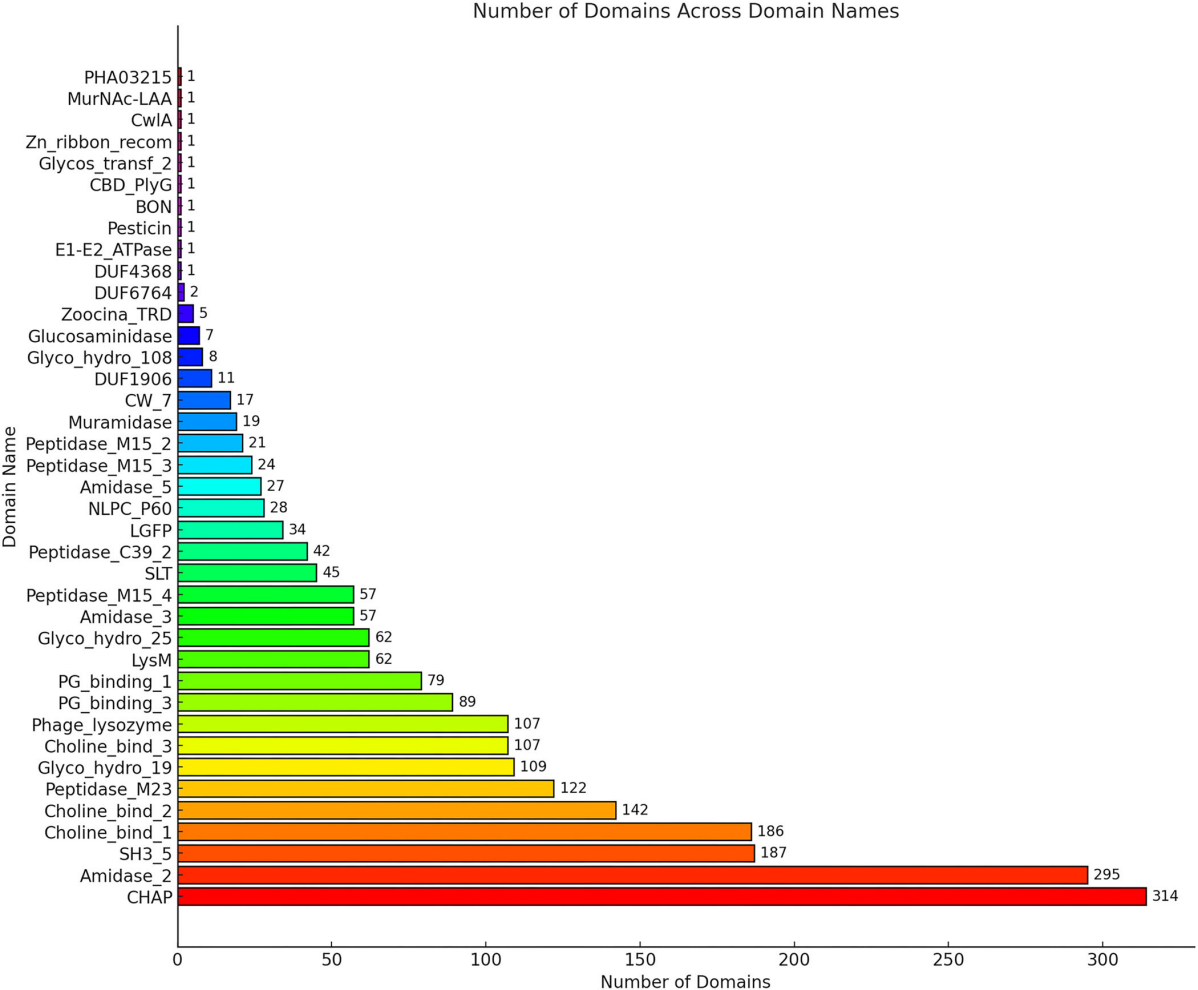
Domain distribution of the endolysins. The numbers on the *y*‐axis represent endolysins' frequency, and the *x*‐axis represents their domain name. A total of 36 domains were found in the endolysin, where the most abundant domain was CHAP an amount with 314, and the second most abundant domain was Amidase_2, with 295.

The Hmmsearch program, part of the HMMER software suite, was used to compare newly identified endolysins against domain profile databases, specifically the Pfam database and the NCBI Conserved Domain Database (CDD), to determine their functional domains. The domain architecture analysis of endolysins revealed that cysteine–histidine–dependent amidohydrolase/peptidase (CHAP) domains were the most prevalent, representing approximately 13.81% of the total data set. These domains are known for their catalytic activity, cleaving peptidoglycan cross‐links in bacterial cell walls. Amidase_2 domains, which account for 12.96%, function by hydrolyzing the amide bond between *N*‐acetylmuramic acid and l‐alanine, contributing to bacterial cell wall degradation. Notably, Choline_bind_1 domains (8.18%) serve as cell wall binding domains (CBDs) that target choline‐containing teichoic acids, particularly in Gram‐positive bacteria, enhancing substrate specificity. Similarly, SH3_5 domains (8.22%) facilitate noncovalent binding to peptidoglycan, playing a crucial role in substrate recognition and localization (Figure 5). In contrast, some domains were relatively rare. For instance, DUF6764 and Zn_ribbon_recom represented only about 0.09% and 0.04% of the data set, respectively. Overall, this broad distribution of protein domains emphasizes the diverse functional roles and evolutionary significance of the endolysins analyzed, offering valuable insights into their structural complexity and potential applications.

A thorough analysis identified a total of 36 distinct domain types within the sequence. Among these, 19 domain types were associated with enzymatic activity, 10 domain types were linked to cell wall binding, and 7 domain types corresponded to unknown functions (Table 2). Additionally, the assessment included evaluating the conditional E‐value with the inclusion and reporting thresholds applied, ensuring that domain identifications met stringent *E*‐value‐based cut‐off criteria for reliable classification.

**Table 2:** Protein domain analysis of endolysins identified through the HMMER program (domain, domain type, Pfam domain, and their activity).

Domain	Domain type^a^	Pfam domain	Activity
Amidase_2	EAD	PF01510.28	*N*‐acetylmuramoyl‐l‐alanine amidase
Amidase_3	EAD	PF01520.21	*N*‐acetylmuramoyl‐l‐alanine amidase
Amidase_5	EAD	PF05382.16	Bacteriophage peptidoglycan hydrolase
Glyco_hydro_19	EAD	PF00182.22	Chitinase class I
Glyco_hydro_25	EAD	PF01183.23	Glycosyl hydrolases family 25
Glyco_hydro_108	EAD	PF05838.15	Glycosyl hydrolase 108
Peptidase_M15_3	EAD	PF08291.14	Peptidase M15
Peptidase_M15_4	EAD	PF13539.9	d‐alanyl‐d‐alanine carboxypeptidase
Peptidase_M23	EAD	PF01551.25	Peptidase family M23
SLT	EAD	PF01464.23	Transglycosylase SLT domain
Glucosaminidase	EAD	PF01832.23	Mannosyl‐glycoproteinendo‐beta‐*N*‐acetylglucosaminidase
Phage_lysozyme	EAD	PF00959.22	Phage lysozyme
CHAP	EAD	PF05257.19	CHAP domain
NLPC_P60	EAD	PF00877.22	NlpC/P60 family
Peptidase_C39_2	EAD	PF13529.9	Peptidase_C39 like family
Muramidase	EAD	PF11860.11	*N*‐acetylmuramidase
E1‐E2_ATPase	EAD	PF00122.23	E1‐E2_ATPase
Pesticin	EAD	PF16754.8	Bacterial toxin homologue of phage lysozyme, C‐term
Glycos_transf_2	EAD	PF00535.29	Glycosyl transferase family 2
Choline_bind_1	CBD	PF01473.23	Putative cell wall binding repeat
Choline_bind_2	CBD	PF19085.3	Choline‐binding repeat
Choline_bind_3	CBD	PF19127.3	Choline‐binding repeat
PG_binding_1	CBD	PF01471.21	Putative peptidoglycan binding domain
PG_binding_3	CBD	PF09374.13	Predicted Peptidoglycan domain
Zoocina_TRD	CBD	PF16775.8	Target recognition domain of lytic exoenzyme
CW_7	CBD	PF08230.14	CW_7 repeat
LysM	CBD	PF01476.23	LysM domain
CBD_PlyG	CBD	PF12123.11	PlyG Cell wall binding domain
SH3_5	CBD	PF08460.13	Bacterial SH3 domain
DUF1906	Unknown	PF08924.14	Domain of unknown function (DUF1906)
DUF6764	Unknown	PF20550.1	Family of unknown function (DUF6764)
DUF4368	Unknown	PF14287.9	Domain of unknown function (DUF4368)
Peptidase_M15_2	Unknown	PF05951.16	Bacterial protein of unknown function (DUF882)
Zn_ribbon_recom	Unknown	PF13408.9	Recombinase zinc beta ribbon domain
LGFP	Unknown	PF08310.14	LGFP repeat
BON	Unknown	PF04972.20	BON domain

*Note:* Three different colours are used to represent three distinct domain types.

^a^ Enzymatically active domain (EAD), cell wall binding domain (CBD).

Approximately 6% of the total endolysin proteins were identified as containing signal peptides, based on concordant predictions from multiple independent computational analyses. This consensus approach enhances the reliability of signal peptide annotation by minimizing tool‐specific biases (Figure S3A).

To assess subcellular localization, the SOSUI tool revealed a diverse distribution: 428 endolysins were predicted in the cytoplasm, 107 in the periplasmic space, 202 extracellularly, 50 at the outer membrane, 18 at the inner membrane, and 163 with unknown localization (Figure S3B). The isoelectric point (pI), calculated using IPC 2.0, ranged from 3 to 13, with molecular weights reaching up to 6.3 kDa among the 968 sequences. The GRAVY index (hydrophobicity) spanned from ‐1.3186 to 0.820, where positive values were linked to membrane proteins and negative values to soluble proteins (Figure S3B). Stability and thermostability were assessed using the Instability Index and Aliphatic Index: values below 40 indicated stability, while values above 55 suggested potential instability (Boro et al. 2024; Figure S3C). Most endolysins were stable (Instability Index < 50) and thermostable (Aliphatic Index > 70). Further analysis with SOSUI identified 56 membrane proteins (6%) and 912 soluble proteins (94%) (Figure S3D). TMHMM analysis showed 49 endolysins contained one transmembrane helix, three had two helices, and 916 sequences lacked transmembrane helices (Figure S3E). Together, these findings highlight the structural and functional diversity of endolysins, underlining their potential as robust agents for targeting bacterial pathogens.

### 3.7 Endolysin Host Prediction

Sequence homology analysis predicted the potential bacterial hosts of the identified endolysins. The results revealed that *Staphylococcus* phages were the most abundant, accounting for 229 endolysins (38.3%), followed by Corynebacterium phages with 107 (17.9%), Streptococcus phages with 68 (11.3%), Pseudomonas phages with 60 (10%), and Propionibacterium phages with 49 (8.2%) (Table S2).

### 3.8 Phylogenetic Analysis

**Figure 6:**
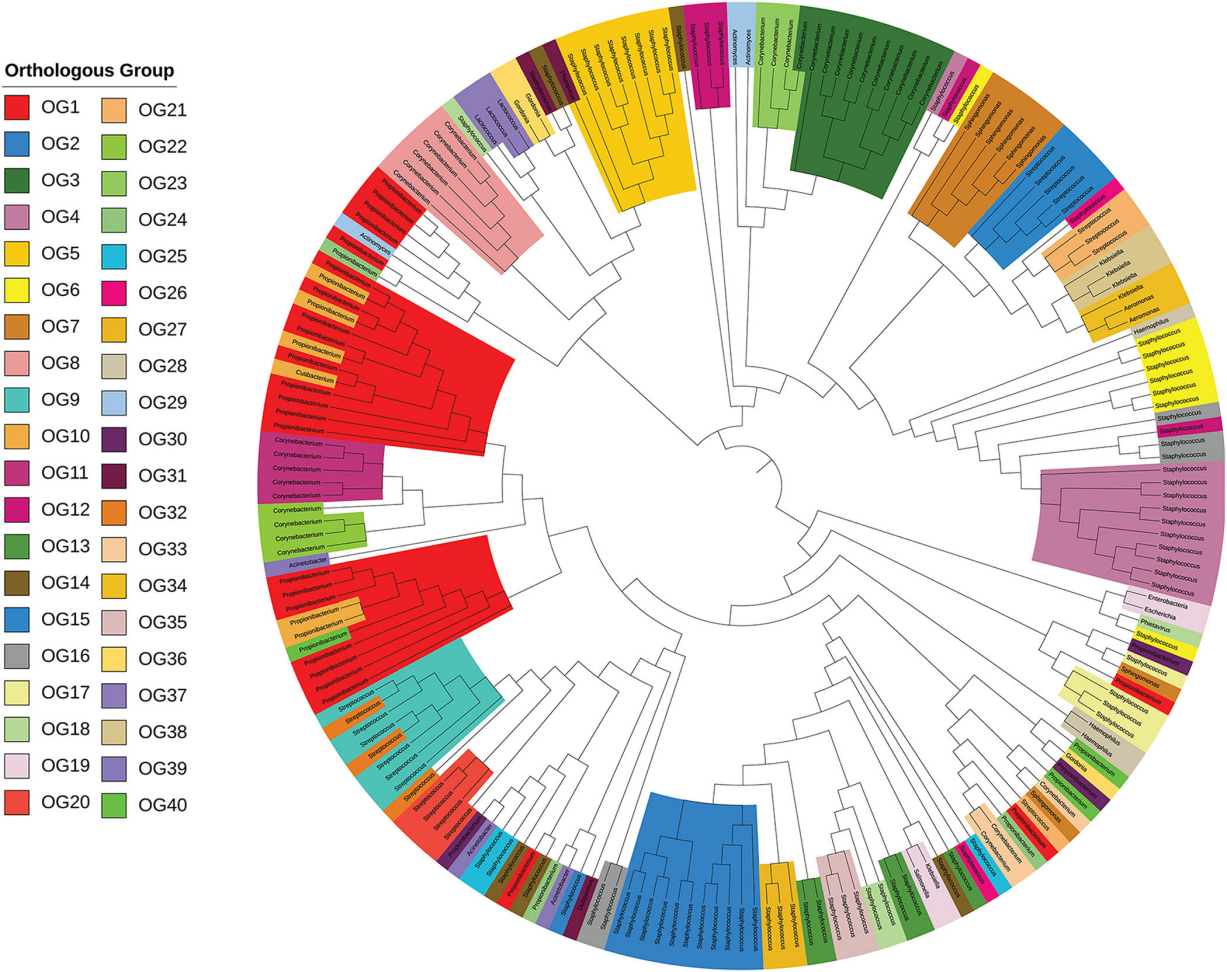
Phylogenetic tree of 968 endolysins categorized into 40 orthologous groups (OGs). The branches are color‐coded according to their respective OG assignments. Orthologous genes are grouped tightly, indicating high sequence similarity and preserved functional roles. However, several notable discrepancies are observed in the present phylogeny, where some orthologues fail to cluster within their anticipated clades. These anomalies may arise due to partial matches in conserved domains, particularly in multidomain endolysin proteins. Unlike single‐domain proteins, multidomain proteins exhibit a complex structural composition where individual domains may vary in evolutionary conservation. In cases where only a portion of a conserved domain aligns due to partial sequence homology, it is likely to influence the tree topology, leading to deviations from expected clustering patterns.

The phylogenetic analysis of 968 endolysins revealed substantial diversity, identifying 723 OGs based on homologous sequence relationships (Figure 6). Notably, 40 OGs contained at least three endolysins each, while the remaining 683 OGs consisted of fewer than three, reflecting the extensive structural diversity and adaptive evolution of endolysins across bacterial environments. These OGs include endolysins predicted to target key pathogenic genera such as *Staphylococcus*, *Corynebacterium*, and *Streptococcus*, highlighting their potential relevance in antimicrobial applications.

**Figure 7:**
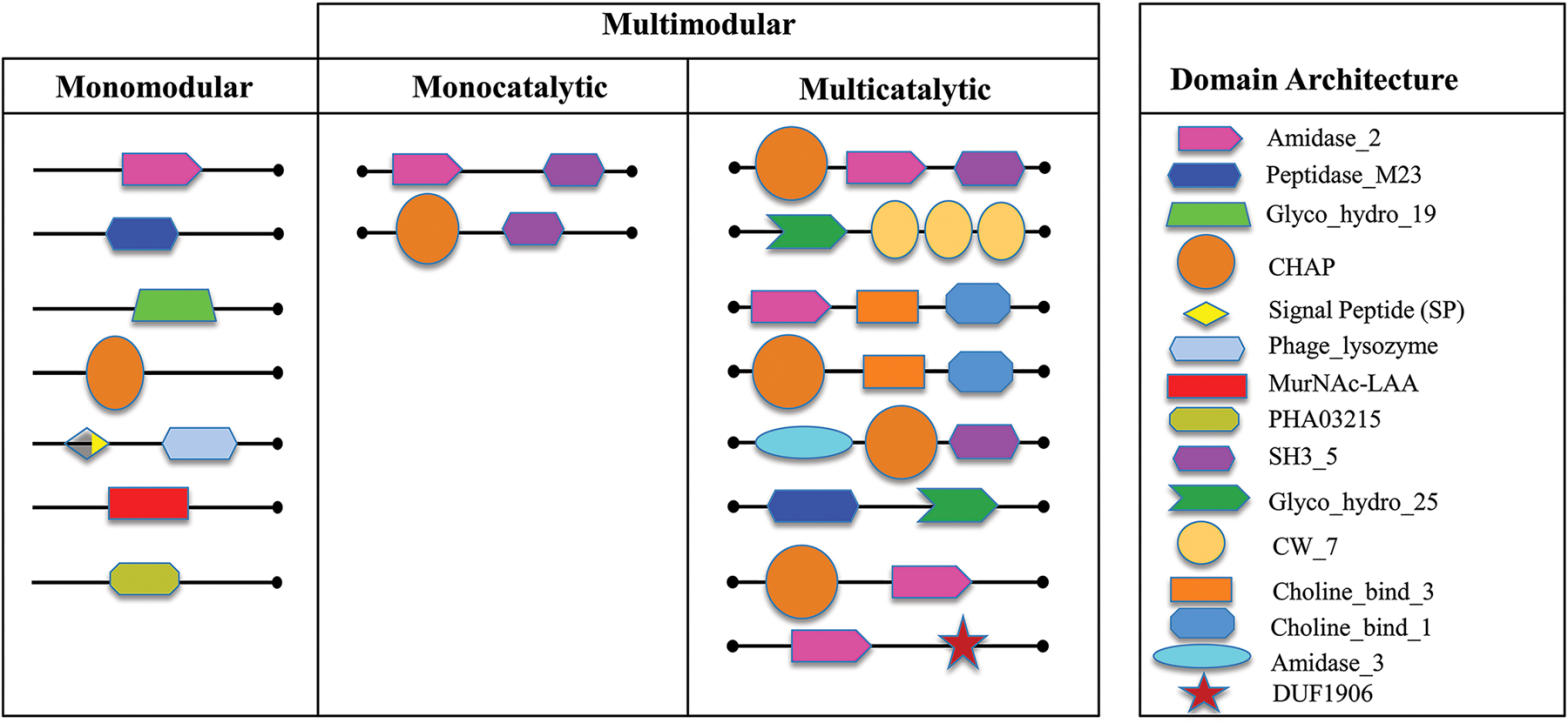
Domain architectures of Bacteriophage proteins across orthologous groups (OGs). This modular classification highlights the diversity and adaptability of bacteriophage proteins, showcasing how combinations of enzymatic and binding domains contribute to targeting and degrading bacterial cell walls effectively.

The protein domain architecture (PDA) analysis revealed a wide range of configurations, including monomodular, monocatalytic, and multicatalytic types (Figure 7). The modular nature of endolysins, particularly the combination of catalytic and binding domains, plays a pivotal role in determining substrate specificity and lytic activity. For instance, variations in SH3 or LysM domains may influence binding affinity to distinct Gram‐positive cell wall structures, while CHAP or Amidase domains affect cleavage mechanisms (Vermassen et al. 2019). Monomodular enzymes carried single enzymatic domains, such as Amidase_2, Peptidase_M23, or CHAP, while monocatalytic multimodular enzymes combined enzymatic domains with cell‐binding domains (CBDs), for example, Amidase_2 + SH3_5 or CHAP + SH3_5. Multicatalytic enzymes showed complex architectures, such as CHAP + Amidase_2 + SH3_5 or Amidase_2 + multiple choline‐binding domains, supporting enhanced catalytic efficiency and substrate targeting.

Unique domain architectures were also identified, with some OGs containing standalone domains like CwlA (OG 30), Choline_bind_3 (OG 9), or SH3_5 (OG 14), illustrating the diverse functional strategies of these enzymes. Overall, the comprehensive domain analysis underscores the evolutionary and functional versatility of skin metagenome‐derived endolysins, reinforcing their potential as candidates for novel antibacterial therapies.

### 3.9 Orthologs Domain Association

**Figure 8:**
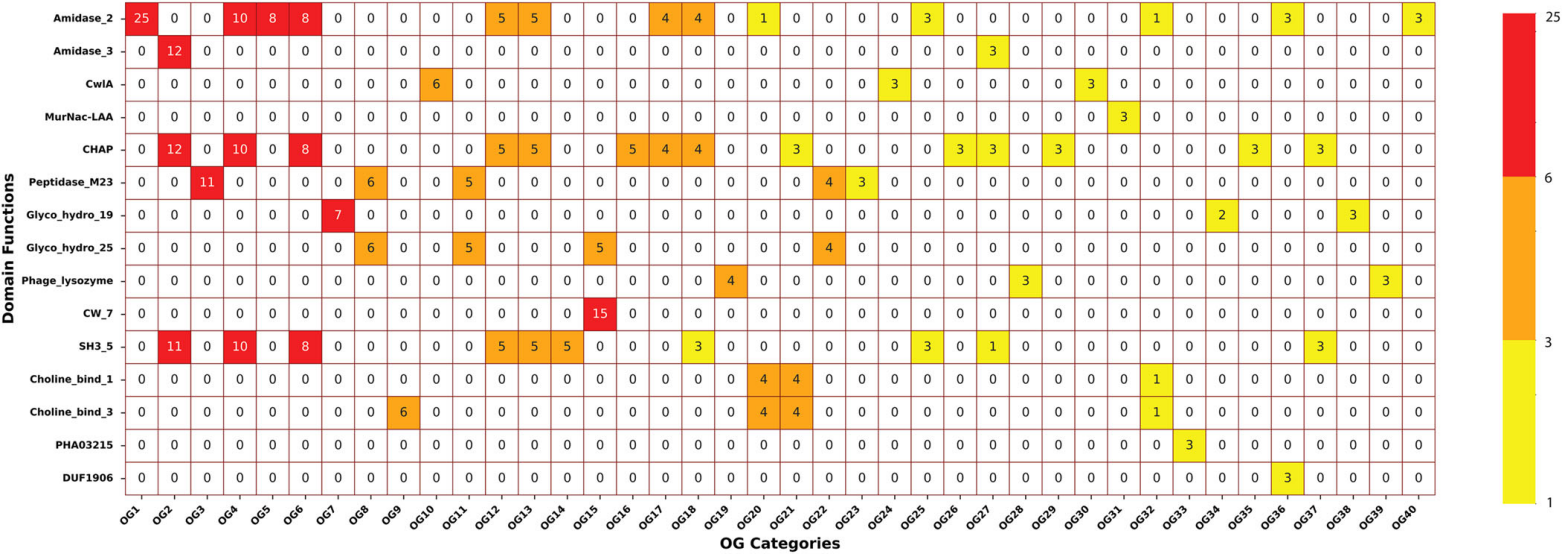
This figure summarizes the distribution of specific protein domains across various orthologous groups (OGs), showing the diversity in enzymatic and binding functions. This heatmap provides insight into the diversity and modularity of bacteriophage proteins by illustrating how domain functions are distributed across different OGs. The frequency of certain domains, such as Amidase_2 and CHAP, highlights their widespread use in bacterial cell wall degradation. Meanwhile, specialized domains with lower occurrences may serve unique functions in fewer bacteriophage proteins, contributing to specific aspects of bacterial host targeting or lysis. This visualization underscores the variability of domain architectures and helps identify domains that are crucial for phage functionality.

The analysis of 40 OGs revealed 15 distinct domains, including 9 enzymatically active domains (EADs), 4 CBDs, and 2 domains of unknown or unrelated functions (Figure 8). Among these, 19 OGs contained only a single catalytic domain, 12 OGs combined EADs with CBDs, and 2 OGs contained only a single CBD. Notably, signal peptide sequences were detected in just 2 OGs (OG 19 and OG 28), suggesting that most endolysins likely rely on alternative, holin‐independent transport mechanisms for membrane translocation.

Among the identified domains, the most prevalent was Amidase_2 (PF01510.28), which hydrolyzes the bond between *N*‐acetylmuramic acid and l‐alanine in the bacterial cell wall, followed by CHAP (PF05257.19), which cleaves peptide bonds in peptidoglycan to facilitate bacterial lysis, and SH3_5 (PF08460.13), a cell wall‐binding domain that contributes to substrate specificity. These conserved domain patterns among orthologs reflect evolutionary pressures to maintain functional motifs essential for enzymatic activity and bacterial cell wall degradation. Overall, the orthologous relationships and domain associations provide key insights into endolysin function and specificity, supporting their potential as targeted antimicrobial agents and guiding the rational selection or engineering of phage‐derived therapeutics (Fernández‐Ruiz et al. 2018).

### 3.10 Host–Orthologs Interaction

**Figure 9:**
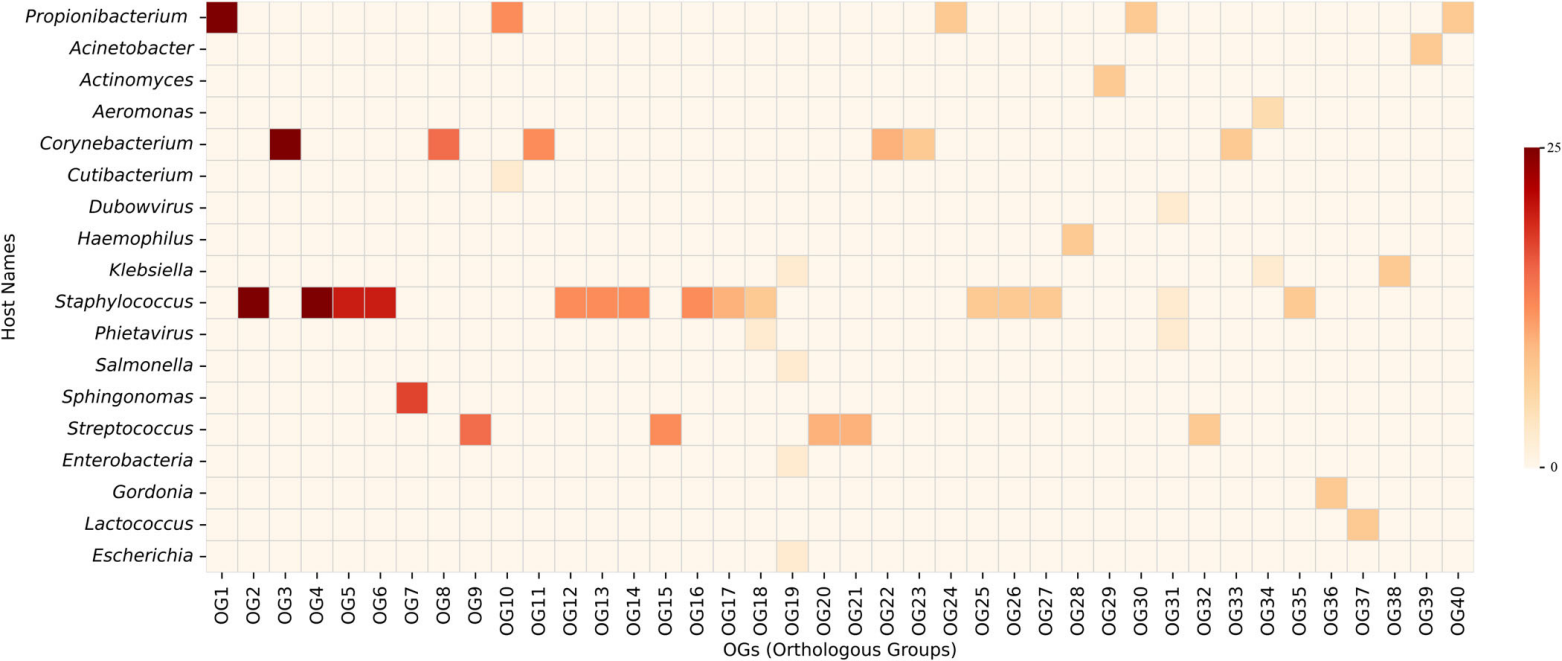
A heatmap depicting associations between targeted host and endolysin orthologous groups. The *x*‐axis represents OGs (OG1 to OG40), while the *y*‐axis lists various host names. The color intensity indicates the frequency of endolysins associated with each host‐OG pair, ranging from light beige (low frequency) to dark red (high frequency).

The associations between OGs and predicted bacterial hosts of endolysins revealed distinct patterns related to host type and domain composition (Figure 9). Endolysins targeting Gram‐negative bacteria were typically composed of a single enzymatic catalytic domain (ECD), whereas those targeting Gram‐positive bacteria often included both an ECD and a CBD, reflecting differences in cell wall architecture.

Notably, several endolysins targeted multiple hosts across different OGs. For example, endolysins active against *Staphylococcus* were distributed across 15 OGs, while those targeting Corynebacterium were found in 6 OGs. Although some OGs showed broad host specificity, others were more restricted. Specific OGs, such as OGs 2, 4, 5, 6, 12, 13, 14, 16, 17, 25, 26, 27, and 35, were predominantly linked to *Staphylococcus*, while OGs 6, 15, 20, 21, and 32 were associated with *Streptococcus*.

Overall, OGs with predicted multi‐taxa targeting were generally confined to bacteria sharing similar Gram‐type features, underscoring the influence of cell wall composition on host specificity. Additionally, unique associations were observed, such as endolysins from phages infecting *Acinetobacter* (OG39), *Actinomyces* (OG29), and *Sphingomonas* (OG7), suggesting adaptations to their distinct cell wall structures. These findings highlight how the domain architecture within OGs shapes the host range and informs our understanding of endolysin‐bacteria interactions.

### 3.11 Taxonomic Analysis of Viral Contigs

Using the vConTACT2 tool, we identified 128 distinct phage variants, each comprising multiple strains, from the analyzed viral contigs. The analysis revealed 22 viral families, which were further classified into 265 distinct genera. These phage variants were classified into 22 viral families and further grouped into 265 distinct genera, highlighting the extensive taxonomic diversity present within the metagenomic data set.

### 3.12 SAR Endolysin Identification

From the 968 endolysins identified in skin metagenomic samples, 75 (7.76%) were predicted to be SAR‐endolysins containing N‐terminal transmembrane domains, as determined by tools such as SOSUI, TMHMM, Phobius, and Topcons. These SAR‐endolysins exhibited a high glycine/alanine content (40%–60%) and a maximum of two basic amino acid residues, indicating structural features that enhance flexibility and facilitate precise activity during phage‐mediated host lysis (Table S3).

### 3.13 Antimicrobial Peptide Identification and Characterization

The segmentation of 968 endolysins into 20‐mer peptides generated 201,303 fragments, which, after removal of duplicates, resulted in 30,419 unique peptide sequences. These distinct peptides were evaluated for antimicrobial potential using AxPEP, AmpGram, and Antimicrobial Peptide Scanner v2, collectively identifying 1517 endolysin‐derived peptides (EPs) as antimicrobials (Table S4).

Additionally, antifungal predictions revealed 307 peptides identified by Antifp and 66 by iAMPpred (with a threshold cutoff of 0.8), of which seven peptides were consistently predicted as antifungal by both tools. iAMPpred further predicted 20 peptides to have antiviral activity, while ToxPred analysis indicated that the majority of peptides (*n* = 37) were nontoxic (Table S5).

### 3.14 Antimicrobial Peptide Stability and Molecular Docking

The three‐dimensional (3D) structures of 37 antimicrobial peptides (AMPs) were predicted using AlphaFold2, leveraging its deep learning capabilities for highly accurate structure modeling based on amino acid sequences. To assess binding interactions, molecular docking of the seven most stable AMPs was performed against selected bacterial protein targets using the HDOCK server (Table 3). The target proteins included the *Staphylococcus epidermidis* virulence factor autolysin (PDB: 4EPC) (Zoll et al. 2012), Beta‐lactamase VIM‐2 (PDB: 5O7N), and the AHL Synthase LasI (PDB: 1RO5) (Gould et al. 2004).

**Table 3:** Docking result of stable antimicrobial peptide with targeted protein.

Antimicrobial peptide^a^	Protein targeted		
	Docking result with(4EPC) kcal/mol	Docking result with (1RO5) kcal/mol	Docking result with (5O7N) kcal/mol
EP‐125	−287.17	−249.47	−188.14
EP‐404	−226.97	−229.47	−179.48
EP‐464	−256.02	−247.34	−189.17
EP‐519	−271.79	−245.47	−178.74
EP‐783	−245.93	−222.57	−194.27
EP‐1270	−262.82	−230.17	−216.43
EP‐1284	−250.45	−229.34	−194.30

^a^ Endolysin‐derived peptide (EP).

### 3.15 MD Simulation of AMP–Protein Complex

GROMACS (version 2023.3) was employed to assess the structural stability and dynamics of the AMP–protein complexes under physiological conditions. Key simulation parameters, including RMSD, RMSF, SASA, and radius of gyration (Rg), were analyzed to evaluate the stability, flexibility, and overall structural integrity of the peptides throughout the simulation. These analyses provided insights into critical regions of the AMPs that may play essential roles in their antimicrobial activity.

### 3.16 RMSD Analysis

**Figure 10:**
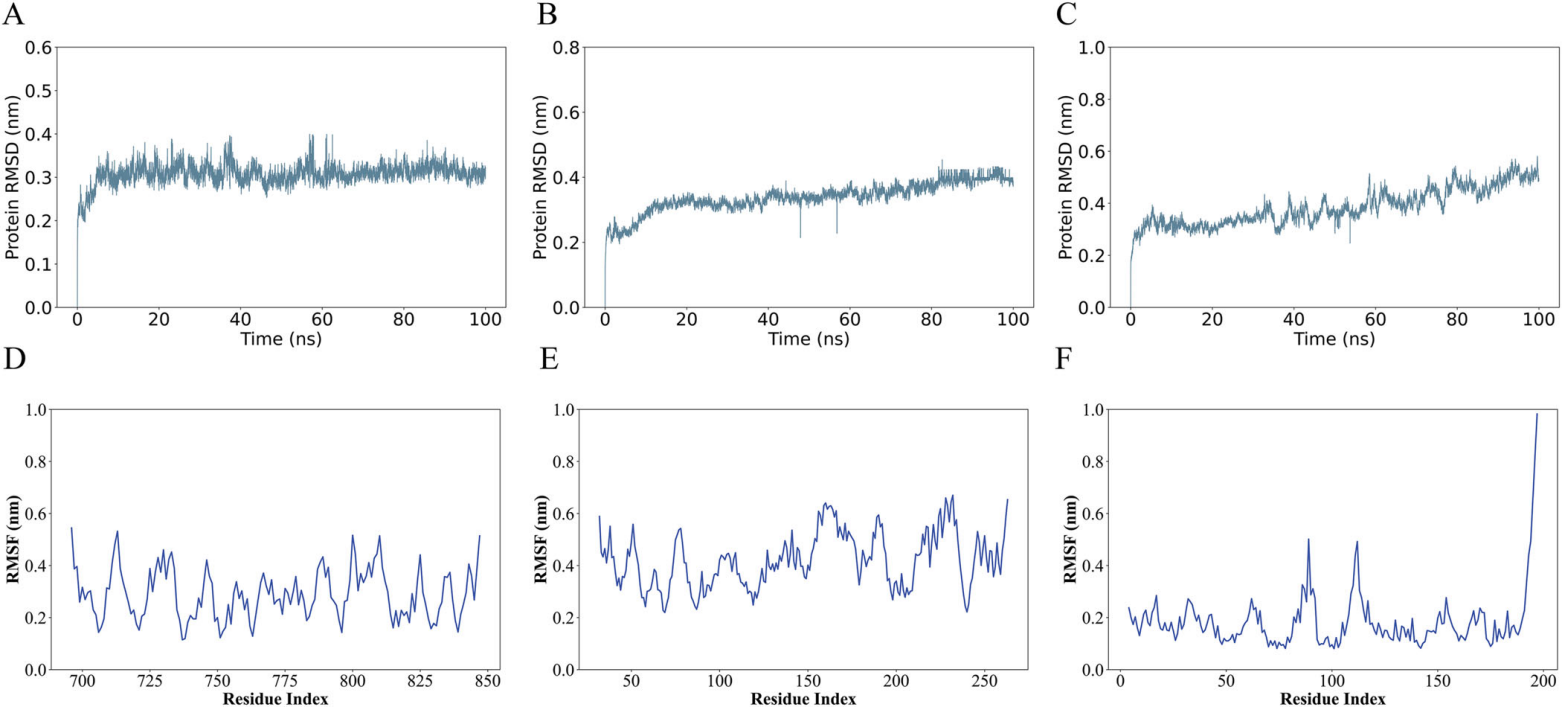
Trajectory analysis of protein–peptide complex over a 100‐ns molecular dynamics simulation. (A) RMSD of autolysin‐peptide (4EPC‐EP519) complex, (B) crystal structure of the AHL synthase lasi and peptides (1RO5‐EP464) complex, (C) RMSD of beta‐lactamase VIM‐2 in complex with (2R)‐1‐(2‐benzyl‐3‐mercaptopropanoyl)piperidine‐2‐carboxylic acid‐peptide (5O7N‐EP464) complex, (D) RMSF of (4EPC‐EP519), (E) RMSF of (1RO5‐EP464), and (F) RMSF of (5O7N‐EP464).

The RMSD analysis was conducted to evaluate the structural stability and conformational changes of the protein–peptide complexes over a 100‐ns MD simulation. Panel A shows early stabilization with RMSD plateauing at 0.3–0.4 nm, indicating a stable protein–peptide complex with minimal conformational changes. Panel B exhibits moderate stability, with RMSD around 0.4 nm and occasional spikes suggesting transient flexibility. Panel C displays the highest fluctuations (0.4–0.6 nm), reflecting notable conformational dynamics and possible weak ligand binding or structural instability (Figure 10A–C).

### 3.17 RMSF Analysis

RMSF analysis was conducted over the 100 ns simulation to assess the local flexibility of protein residues. Panel D showed predominantly low RMSF values (0.2–0.4 nm), indicating stable residues with limited mobility, except for modest peaks (~0.6 nm) likely corresponding to flexible loop or terminal regions. Panel E revealed increased flexibility (0.3–0.6 nm) with multiple peaks, suggesting dynamic segments influenced by peptide interactions. Panel F exhibited the highest flexibility, with a major peak exceeding 0.8 nm near the C‐terminus, indicating a highly dynamic or unstructured region, while the remaining protein regions displayed moderate fluctuations (Figure 10D–F).

### 3.18 SASA Analysis

**Figure 11:**
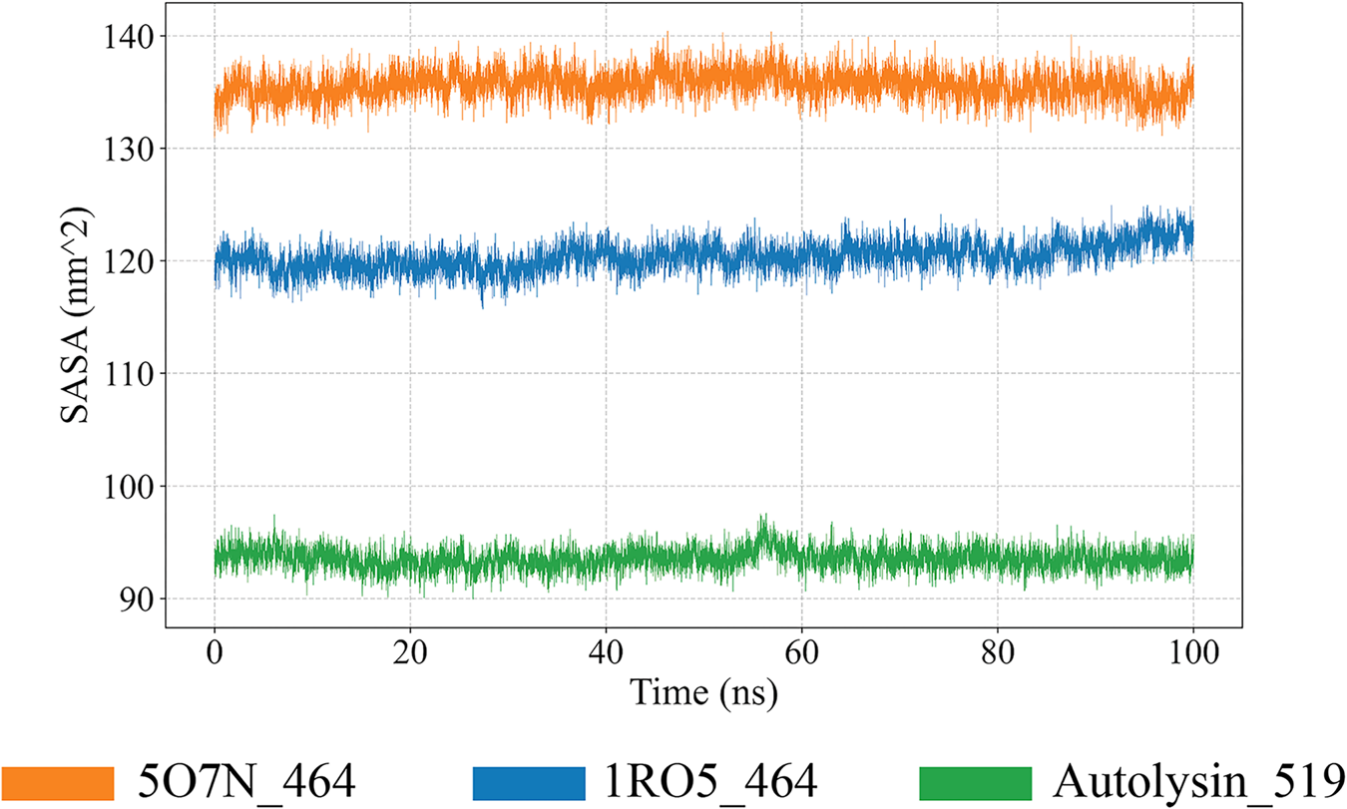
Trajectory analysis of protein–peptide complex over a 100‐ns molecular dynamics simulation. Solvent accessible surface area (SASA) over time, the *x*‐axis represents the simulation time in nanoseconds (ns) and the *y*‐axis displays the SASA in nanometers squared (nm²).

SASA analysis revealed distinct conformational differences among the protein–peptide complexes. The 5O7N_464 complex exhibited the highest SASA values, fluctuating between 135 and 140 nm², indicating a more solvent‐exposed or open conformation. The 1RO5_464 complex showed moderate SASA values (120–125 nm²), suggesting an intermediate level of solvent accessibility and stability. In contrast, Autolysin_519 had the lowest SASA, around 90–95 nm², reflecting a more compact structure with minimal solvent exposure (Figure 11). The consistent fluctuations in SASA values across all systems suggest stable dynamics, with variations highlighting differences in structural compactness and potential functional states.

### 3.19 Radius of Gyration (Rg) Analysis

**Figure 12:**
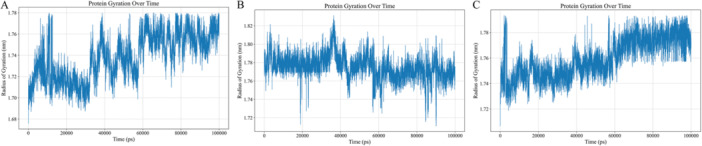
Trajectory analysis of protein–peptide complex over a 100‐ns molecular dynamics simulation. Radius of gyration (Rg) over time. (A) Rg of (4EPC‐EP519), (B) Rg of (1RO5‐EP464), (C) Rg of (5O7N‐EP464). The *x*‐axis represents simulation time in picoseconds (ps), while the *y*‐axis indicates the radius of gyration in nanometers (nm).

The radius of gyration (Rg) analysis (Figure 12), reveals distinct compactness and structural stability profiles for the three simulated protein systems (A, B, and C) over time. System A exhibits a consistently compact structure with Rg fluctuating minimally between 1.70 and 1.76 nm. System B displays a slightly more dynamic structure, indicated by a wider Rg range of 1.72 to 1.82 nm, suggesting larger conformational changes. In contrast, System C shows a progressive increase in Rg throughout the simulation, indicating a gradual expansion or loosening of its structure. These Rg profiles highlight significant differences in the flexibility and overall folding behavior of the three protein systems.

## 4 Discussion

Human skin diseases, driven by diverse microbial species, range from common throat infections to life‐threatening conditions like toxic epidermal necrolysis, necrotizing fasciitis, severe burns, melanoma, gangrene, and severe psoriasis. If left untreated, this infection can lead to organ failure or even death. The growing prevalence of antibiotic‐resistant bacteria in the human skin microbiome highlights the urgent need for alternative therapeutics. Endolysins and endolysin‐derived antimicrobial peptides present a promising alternative to conventional antibiotics (Schmelcher et al. 2012; Lin et al. 2017; Mondal et al. 2020; Gontijo et al. 2021; Rahman et al. 2021). This study provides a large‐scale metagenomic analysis of the human skin phageome with an emphasis on endolysins and their encoded AMPs. By mining over 1500 skin‐derived metagenomes, this study identified nearly a thousand novel endolysins and 37 endolysin‐derived AMPs with potential therapeutic value. These findings establish a critical foundation for the development of targeted alternatives to antibiotics for skin infections, particularly those involving MDR pathogens.

The identified phages displayed significant genomic variability, with average GC content at 56% and coding efficiency around 72%, indicative of compact, host‐adapted genomes. Using protein‐sharing genome clustering approaches (Versoza et al. 2022; Oliveira et al. 2019), we identified 81 unique human skin microbiome phage clusters alongside 172 singletons, a result highlighting a promising level of phage diversity within this environment compared to previous investigations (Smith et al. 2013). Subsequent phylogenetic evaluation corroborated these cluster assignments, demonstrating that the identified groups typically formed distinct and well‐defined monophyletic clades, further supporting the robustness of our clustering approach.

Further analysis of the identified phages revealed notable inconsistencies in their taxonomic assignment at both the host group and family levels when compared to the current ICTV classification. While our study identified 37 distinct host groups and 8 families based on sequence analysis, a significant proportion of these remained unassigned according to established taxonomic frameworks. Notably, proteomic tree analysis indicated that the currently assigned phages did not consistently form clear monophyletic clades, a fundamental requirement for single‐family classification by the ICTV (Turner et al. 2021; Zhang et al. 2023). This finding underscores the considerable complexity and diversity inherent within the human skin microbiome phage population, particularly evident in the significant variation observed in their endolysin domain architectures. This structural diversity not only reflects the intricate evolutionary history of these phages but also hints at a broad range of potential therapeutic applications. Interestingly, we observed associations between specific endolysin domain variations and the microbial hosts present in the human skin, suggesting that environmental factors and the selective pressures imposed by host specificity play a crucial role in shaping phage evolution within this unique ecosystem.

Interestingly, despite the presence of observable similarities in their domain architectures and sequence homology, phylogenetic analysis of endolysins specifically targeting particular human skin microbiome hosts did not consistently resolve into distinct monophyletic clades. This suggests that the evolutionary relationships among these enzymes are more intricate than initially hypothesized, implying that factors beyond the primary domain structure significantly contribute to their phylogenetic classification (Davison et al. 2016). The observed inconsistency in the formation of clear monophyletic clades further indicates that the evolutionary trajectory of these endolysins is not solely dictated by their structural domains but is also influenced by other complex genetic and ecological dynamics within the skin environment (Knight et al. 2021; Neri et al. 2022).

A key observation contributing to this complex evolutionary landscape is the significant exchange, acquisition, or deletion of functional domains among bacteriophages infecting diverse hosts within the skin microbiome. These dynamic genomic rearrangements, which complicate straightforward phylogenetic classification, are likely driven by mechanisms such as homologous recombination during co‐infections and adaptive genetic changes selected by environmental pressures (Brüssow et al. 2004). These evolutionary processes highlight the inherent fluidity and remarkable adaptability characteristic of phage evolution (Jia et al. 2023; Smug et al. 2023). Moreover, horizontal gene transfer events and the potential for phages to infect across species boundaries likely contribute significantly to the extensive genetic diversity observed within endolysins targeting bacteria colonizing the human skin (Moller et al. 2019).

This substantial diversity within the human skin microbial community, coupled with the intricate evolutionary dynamics of their associated phages and endolysins, paradoxically enhances the therapeutic potential of these enzymes. The broad range of bacterial targets present on the skin suggests a rich source of endolysins with varying specificities, making them particularly valuable in the fight against MDR bacterial strains. Moreover, the inherent specificity of endolysins for their bacterial targets, combined with their relatively lower propensity for inducing rapid resistance development compared to traditional antibiotics (F. M. Khan, Rasheed, et al. 2024), positions them as promising candidates in addressing the escalating global public health threat posed by MDR infections. The very complexities observed in the evolutionary dynamics of these endolysins underscore the remarkable adaptability of bacteriophages, a characteristic that can be strategically harnessed for the development of innovative and targeted therapeutic strategies against challenging microbial infections prevalent on the human skin.

The domain architecture analysis of the identified endolysins revealed a high degree of diversity, with many displaying multicatalytic configurations, particularly within phages associated with the human skin microbiome. Among these, CHAP domains were the most prevalent, suggesting a potentially significant role in host cell wall degradation. The frequent occurrence of CHAP domains aligns with their known functional versatility and effectiveness. Notably, one CHAP‐containing endolysin identified from an uncultured phage data set in this study corresponds to a protein previously reported to exhibit superior lytic activity compared to conventional lysozymes (Sanz‐Gaitero et al. 2013), highlighting its potential relevance in therapeutic or biotechnological applications. Indeed, CHAP endolysins, as a class, can demonstrate superior activity against bacterial cell walls compared to other muramidases or lysozyme‐like enzymes, which often possess more limited efficacy (Alreja et al. 2024). Similar to findings in gut and respiratory tract phageomes (Rybicka and Kaźmierczak 2025), the skin phageome exhibits rich domain diversity and host‐adapted architectures. However, the predominance of CHAP domains and the unique occurrence of SAR endolysins suggest that skin‐specific phages have evolved distinct lytic strategies, likely influenced by surface‐associated bacterial communities and host immune interactions. This discovery of prevalent and highly active CHAP domains within the skin phageome significantly broadens our understanding of endolysin diversity and opens exciting new avenues for the creation of chimeric endolysins. By strategically combining the strengths of multiple functional domains, such as in the example of CHAPk‐SH3bk (C. Wang, Zhao, et al. 2024; Haddad Kashani et al. 2018), it may be possible to engineer endolysins with enhanced therapeutic outcomes. Ultimately, a comprehensive classification of these diverse endolysins contributes significantly to our understanding of viral ecology, intricate phage‐host dynamics within the skin microbiome, and the vast potential applications of phages and their enzymes in therapeutic contexts.

In recent years, AMPs have gained significant attention due to their potential to combat antibiotic‐resistant pathogens (Alaoui Mdarhri et al. 2022; Xuan et al. 2023; Cresti et al. 2024). Several studies have also investigated synergistic interactions between AMPs, endolysins, and conventional antibiotics, suggesting enhanced efficacy when used in combination (Yang et al. 2015; Gontijo et al. 2021). In this study, we identified 37 novel AMPs derived from human skin microbiome phage endolysins. Notably, several of these peptides exhibited promising antifungal, antiviral, and nontoxic properties (Islam 2024; Tang et al. 2024; Gallardo‐Becerra et al. 2024). To assess their stability, MD simulations were performed, revealing that seven peptides maintained structural stability over time. These seven peptides were subsequently subjected to molecular docking studies with three therapeutically relevant targets, including *Staphylococcus epidermidis* autolysin, Beta‐lactamase VIM‐2, and AHL Synthase LasI. Among the docked complexes, peptide EP‐519 exhibited the lowest RMSD when bound to autolysin, indicating high structural stability. Meanwhile, EP‐464 demonstrated superior binding affinity and stability in complexes with both VIM‐2 and LasI. Autolysin, a key virulence factor in *S. epidermidis*, plays a central role in bacterial colonization and pathogenicity. Prior studies have shown that autolysin‐deficient mutants of *Staphylococcus* exhibit significantly reduced virulence, making it a compelling therapeutic target (Ito and Amagai 2022; M. Wang, Li, et al. 2024). Within the autolysin‐AMP complexes, peptide EP‐519 demonstrated the highest stability (minimal RMSD). Conversely, EP‐464 exhibited strong and stable binding to both Beta‐lactamase VIM‐2 and AHL Synthase LasI, suggesting its potential to inhibit antibiotic resistance and quorum sensing mechanisms. The identification of novel antimicrobial peptides such as EP‐464 and EP‐519, with dual antifungal and quorum‐sensing inhibitory properties, highlights the untapped potential of the skin virome for next‐generation antimicrobials. These findings lay the groundwork for future synthetic biology approaches aimed at engineering targeted endolysins or AMPs for topical applications, with reduced resistance risk and broad‐spectrum activity. Overall, these findings not only deepen our understanding of the endolysin‐derived AMP repertoire within the human skin phageome but also highlight their significant potential as candidates for next‐generation antimicrobial therapies, offering a viable strategy to address the growing global threat of antibiotic‐resistant skin infections.

While these findings offer compelling insights, the study is inherently limited by its in silico nature. Tools like HMMER, Pfam, and AMP predictors may miss novel domains or generate false positives due to sequence‐based constraints. Likewise, docking simulations, while informative, do not guarantee in vivo efficacy or stability. Therefore, future work should focus on experimental validation, including antimicrobial assays, cytotoxicity tests, and stability studies under physiological conditions. Long‐term goals may include synthetic biology approaches to engineer chimeric endolysins and optimized AMPs for topical applications, especially targeting MDR skin infections. By harnessing the specificity, potency, and evolutionary adaptability of skin phage‐derived proteins, we can pave the way toward innovative and precision‐targeted antimicrobials in the post‐antibiotic era.

## 5 Conclusion

This study presents a comprehensive metagenomic analysis of the human skin microbiome, identifying 968 diverse phage‐derived endolysins, including SAR variants, and uncovering novel antimicrobial peptides with therapeutic potential. The findings reveal significant genomic and structural diversity among skin phages, with CHAP domains being predominant and many endolysins exhibiting complex, host‐adapted architectures. Notably, 37 novel AMPs were identified, and *in silico* analyses demonstrated that several, such as EP‐519 and EP‐464, exhibit strong stability and binding affinity to key pathogenic targets. These results underscore the promise of skin microbiome‐derived endolysins and their encoded peptides as next‐generation antimicrobial agents, particularly in the fight against MDR pathogens.

## Author Contributions

**Jibon Kumar Paul:** methodology, validation, visualization, writing – original draft. **Arzuba Akter:** conceptualization, investigation, funding acquisition, methodology, validation, visualization, writing – review and editing, writing – original draft, software, formal analysis, project administration, data curation, supervision, resources. **Nurnabi Azad Jewel:** investigation, methodology, validation, writing – review and editing, supervision, formal analysis. **Mohimenul Haque Rolin:** methodology, validation, visualization. **Daniyal Karim:** methodology, validation, formal analysis. **Raphael Kabir Niloy:** methodology, visualization, validation. **Shakhinur Islam Mondal:** conceptualization, funding acquisition, investigation, writing – original draft, methodology, validation, visualization, writing – review and editing, software, formal analysis, project administration, data curation, supervision, resources.

## Ethics Statement

The authors have nothing to report.

## Conflicts of Interest

The authors declare no conflicts of interest.

## Supporting information


**Figure S1:** A heatmap to visualize the PEQ values among the phage genomes. Based on these PEQ values, 696 phage genomes were grouped into 81 clusters and 171 singletons. The phage genome accessions are color‐coded according to their respective cluster identities, with distinct colors representing different clusters.

**Figure S2:** The taxonomic assignment of human skin microbiome phages at both the host group and family levels. Using the ICTV taxonomic classification system, a total of 37 host groups were identified, including a diverse range of bacterial genera.

**Figure S3:** Endolysin Biochemical Characterization. A) 6% of endolysins have signal peptides, a feature crucial for targeting extracellular activity, enhancing their ability to interact with bacterial cell walls effectively. B) Shows the GRAVY index, where hydrophobic proteins (positive values) are associated with membranes, and hydrophilic proteins (negative values) are soluble. This is critical for understanding protein localization and interactions, which influence endolysin design for therapeutic applications. C) Illustrates stability metrics, with most endolysins being stable (Instability Index < 50) and thermostable (Aliphatic Index > 70). This stability is vital for their functionality in diverse environments. D) 94% of endolysins are soluble proteins and 6% are membrane‐bound. This distinction is significant because soluble proteins are often involved in enzymatic activity, while membrane proteins may play roles in interactions with bacterial cell walls or membranes. E) Indicates transmembrane helix predictions, where most endolysins lack helices, suggesting they are primarily soluble and not embedded in membranes. This supports their role in extracellular or cytoplasmic activity.

**Table S1:** Quality Assessment of Viral Genomes Based on CheckV Classification.

**Table S2:** Endolysin host predition.

**Table S3:** Sequence of SAR (Signal Arrest Release) Endolysins.

**Table S4:** Endolysin‐derived peptides.

**Table S5:** List of non‐toxic peptides.

## Data Availability

The data that supports the findings of this study are available in the supplementary material of this article. The raw data supporting the findings of this study are available from the corresponding author upon reasonable request.
