# Supplementary figures and images for: Exploration of Human Skin Phageome to Reveal Endolysins and Novel Antimicrobial Peptides for Therapeutic Applications
Source: Microbiologyopen. 2025 Nov 9;14(6):e70115. doi: 10.1002/mbo3.70115 (PMC12597775; doi:10.1002/mbo3.70115)

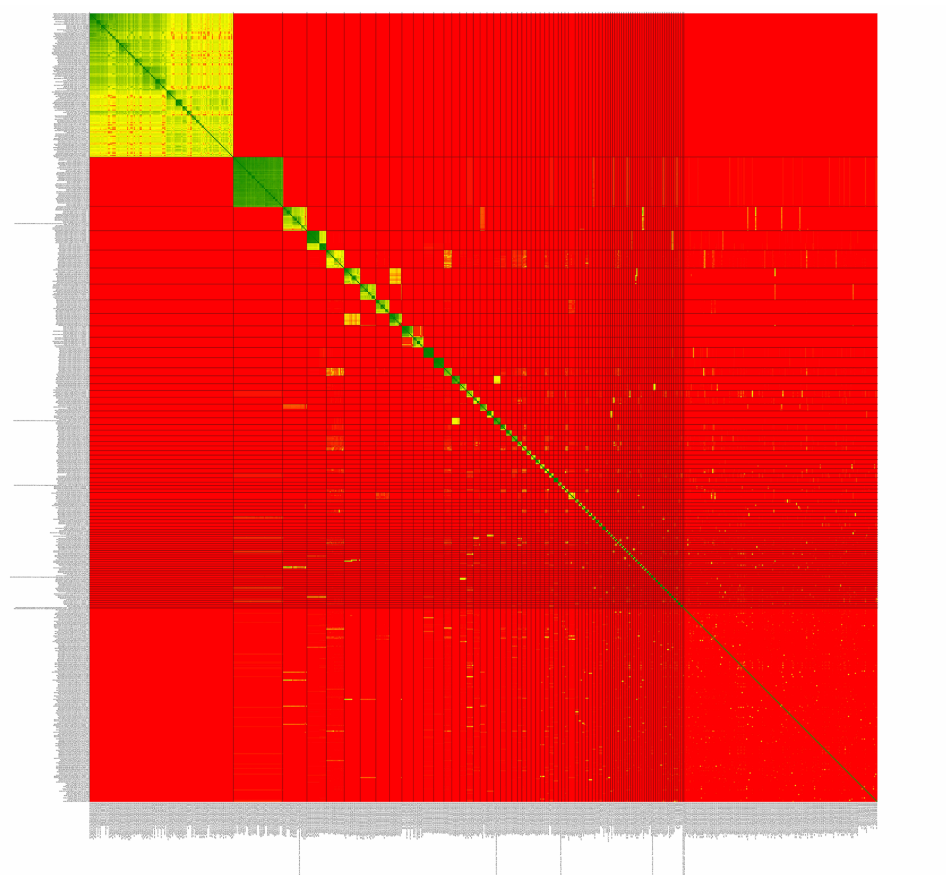

Supplement: Supplementary file 1 — Figure S1: A heatmap to visualize the PEQ values among the phage genomes. Based on these PEQ values, 696 phage genomes were grouped into 81 clusters and 171 singletons. The phage genome accessions are color‐coded according to their respective cluster identities, with distinct colors representing different clusters. [file MBO3-14-e70115-s003.pdf]

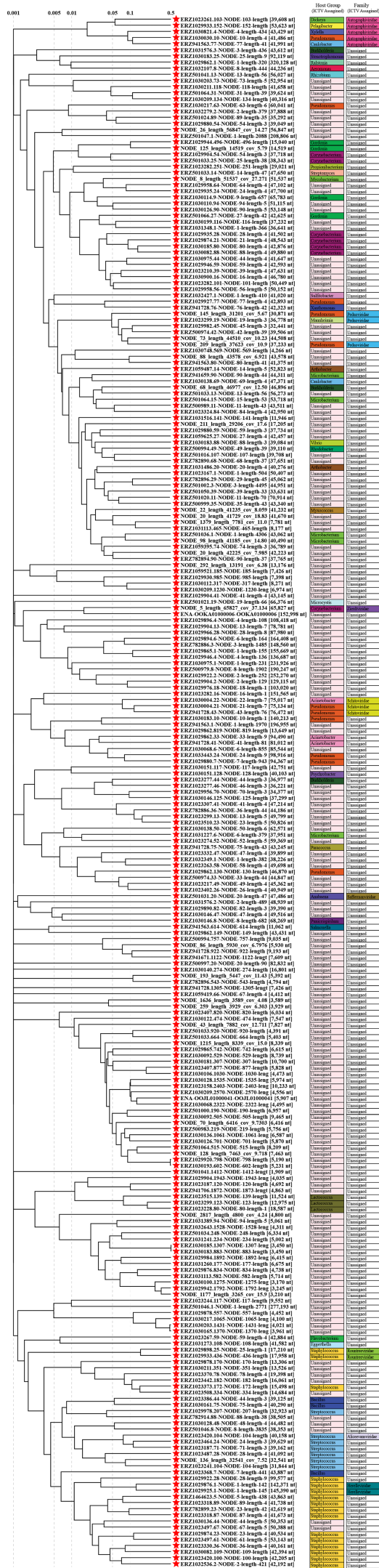

Supplement: Supplementary file 2 — Figure S2: The taxonomic assignment of human skin microbiome phages at both the host group and family levels. Using the ICTV taxonomic classification system, a total of 37 host groups were identified, including a diverse range of bacterial genera. [file MBO3-14-e70115-s004.pdf]
