# Supplementary material for: Exploration of Human Skin Phageome to Reveal Endolysins and Novel Antimicrobial Peptides for Therapeutic Applications
Source: Microbiologyopen. 2025 Nov 9;14(6):e70115. doi: 10.1002/mbo3.70115 (PMC12597775; doi:10.1002/mbo3.70115)
Supplement: Supplementary file 4 — Table S1: Quality Assessment of Viral Genomes Based on CheckV Classification. [file MBO3-14-e70115-s008.docx]

**Table S1. Quality Assessment of Viral Genomes Based on CheckV Classification**

| **Quality Category** | **Number of Genomes (n)** | **Description** |
| --- | --- | --- |
| Complete | 299 | Genomes estimated to be ≥90% complete and circular/linear with defined termini. |
| High-quality | 638 | Genomes estimated to be ≥90% complete but not closed. |
| Medium-quality | 1,039 | Genomes estimated to be 50–90% complete. |
| Low-quality | 88,713 | Genomes estimated to be <50% complete. |
| Not-determined | 763,282 | Completeness could not be determined due to lack of reference or fragmented input. |

**Note:** Genome completeness was predicted using CheckV, a quality estimation tool designed for viral metagenomes. Categories are based on genome length, terminal features, and comparison with reference viral databases.
