# Supplementary material for: Exploration of Human Skin Phageome to Reveal Endolysins and Novel Antimicrobial Peptides for Therapeutic Applications
Source: Microbiologyopen. 2025 Nov 9;14(6):e70115. doi: 10.1002/mbo3.70115 (PMC12597775; doi:10.1002/mbo3.70115)
Supplement: Supplementary file 6 — Table S3: Sequence of SAR (Signal Arrest Release) Endolysins. [file MBO3-14-e70115-s005.docx]

**Table S3. Sequence of SAR (Signal Arrest Release) Endolysins**

>ERZ1030108.2705-NODE-2705-length-3393-cov-3.977232_6 # 3147 # 3392 # 1 # ID=10881_6;partial=01;start_type=GTG;rbs_motif=None;rbs_spacer=None;gc_cont=0.667

MNRLKRWLAEAGNDLAIIWMVEPRLFLWPLILLVFAIGIFIVPKADAASIPTAAEQHRRT

LVRAAHAEWGLGAPVATFAAQV

>ERZ941728.19290-NODE-19291-length-1375-cov-1.618939_2 # 140 # 589 # 1 # ID=12665_2;partial=00;start_type=ATG;rbs_motif=GGAG/GAGG;rbs_spacer=5-10bp;gc_cont=0.376

MPKKNTVLALGKDGLELIKNFEGCVLKVYLDAIGLPTIGYGHLIKPGESFTKITQKEAED

LLKSDAQIFVDGVNKLLEVNVTQNQFDALVSIAFNIGLGNLKSSTLLRLVNAGDYKGAAD

QFPRWNKAGGKVLNGLTKRRNAERDLFLK

>ERZ1023433.3-NODE-3-length-570993-cov-253.149037||0_partial_26 # 20451 # 20966 # 1 # ID=40373_26;partial=00;start_type=ATG;rbs_motif=GGxGG;rbs_spacer=3-4bp;gc_cont=0.562

MNPSIVKRCLVGAVLAIATTLPGFQSLHTSVEGLKLIADYEGCRLQPYQCSAGVWTDGIG

NTSGVVPGKTITERQAAQGLINNVLLTEKRLDACLTVKPPQHVYDALVSIGFNVGTGAIC

RSTMVSYINRQQWWLACNQLPRWIYVNGVKNKGLENRRARELAWCLKGTGA

>ENA-OOMU01000108-OOMU01000108.1-human-skin-metagenome-genome-assembly--contig:-NODE-108-length-4710-cov-5.147583||full_1 # 1 # 477 # -1 # ID=41267_1;partial=10;start_type=ATG;rbs_motif=GGAG/GAGG;rbs_spacer=5-10bp;gc_cont=0.488

MSAKIKTGIAGGICSVAIIIGLVLDNGHVRTNQRGLELIGNAEGCRRDPYNCPAGILTDG

IGNTYGVKPGNRKTDEQIAADWEKNILDAEQCVNRYANGANLPDNTFSSAVEITFNAGCP

TMQKSTMFRLFRQGKLVAACNELPRWVYANGAKLNGLVT

>ERZ500974.1561-NODE-1561-length-2618-cov-3.923527||full_4 # 2228 # 2617 # -1 # ID=53661_4;partial=01;start_type=Edge;rbs_motif=None;rbs_spacer=None;gc_cont=0.767

VVGGWVARTPVARVLVAAGALTAWDVFLGYVTGTVPPSSGGGGGSDGSTAYAGPYRNGSK

DPGAPGPTGGPVWRIQDRLKRAYASYAGHLATDGWFGNATEGAVREFQRRSRLTDDGVVG

PITWAALRL

>ERZ1023300.8907-NODE-8909-length-1568-cov-1.923331||full_1 # 1 # 1092 # -1 # ID=97184_1;partial=10;start_type=ATG;rbs_motif=GGA/GAG/AGG;rbs_spacer=11-12bp;gc_cont=0.700

MRSAKLVVGIVIGIILLSLVVVVILGDGSDDRCRPNGAITGGGGVPAGELSLPIAEGDYV

VSSTYKSADRPGHRGIDLAASGGTPMYAMAPGVVTAAGPASGFGNWIVIDHTIDGAPYST

VYGHMWDDGVAVKVGDTVTAGQLIGAVGSNGQSTGAHLHLEVWQGGRLTGGSETDPQPWL

DRAANPGSGGGAPPTDESTTSRQPSPPSSGEMPDSDKIASKENLQVDSIRVARAVAQRFP

DVKTIGGWRPVDAYPDHPSGRAVDIMIPDYSSSEGRELGNRIKNYLLTHREFFHLEYLIW

RQQYIPATGQPNQMEDRGDPTQNHFDHVHVTVAGGGKPTPNQRYGAAPTDGADDSSETTG

AAGG

>NODE_70_length_64725_cov_8.237560||full_27 # 23797 # 24309 # -1 # ID=132335_27;partial=00;start_type=GTG;rbs_motif=AGxAGG/AGGxGG;rbs_spacer=5-10bp;gc_cont=0.587

MKSIVKRCSVAAVLALAALMPDFRLLNTSPEGLALIADLEGCRLTPYQCSAGVWTSGIGH

TAGVVPKGEITERQAAENLVADVMNVEKRLAVCVPVEMPQHIYDALVSFSFNVGTGAACR

STLVSYIKRHQWWQACDQLTRWVYVNGSINKGLENRRARERAYCLKGVSQ

>NODE_7106_length_530_cov_2.688421_1 # 73 # 528 # 1 # ID=135672_1;partial=01;start_type=ATG;rbs_motif=AGGAG/GGAGG;rbs_spacer=11-12bp;gc_cont=0.414

MKSTTKKIKTTLAGVAALFAVFAPSFVSAQESSTYTVKEGDTLSEIAETHNTTVEKLAEN

NHIDNIHMIYVGQELVIDGPAVPVAPASTTYEAPAAQDEAVSATVTETTEVAEETPVVSE

TVSEETVASTVSGSEAEAKEWIAQKESGGSYT

>ERZ1030171.2250-NODE-2250-length-2659-cov-5.438556||full_4 # 2359 # 2658 # 1 # ID=141530_4;partial=01;start_type=ATG;rbs_motif=AGGAGG;rbs_spacer=5-10bp;gc_cont=0.580

MKKLGLILAAILCLVVLIVVLLDDSDEAGQCTPLGAGGGSATAVIDGDYAYPTDKSKIST

SSAFGLREGGEFHQGMDIAGPAGTPVFAFADGTVVNAQDS

>ERZ1023189.49-NODE-49-length-33275-cov-25.355087||full_28 # 16068 # 16613 # 1 # ID=141611_28;partial=00;start_type=ATG;rbs_motif=None;rbs_spacer=None;gc_cont=0.407

MMILTKTRKALGVCSVITVMGLMYAQFGGELRLSPVGAEIIGNAEGCRRDPYQCPADVLT

VGIGSTEYGGKKINPKHRYTDLEIAERWKNDIVIAERCVNKYGNGEMLPQSVFDSAVSIT

FNVGCGAVSKSTMFKYLRAKQYEKACGEFPRWVYASGKKLAGLVVRREKEKALCLADLKL

P

>ERZ1030120.1934-NODE-1934-length-6610-cov-24.252326_9 # 6463 # 6609 # 1 # ID=150442_9;partial=01;start_type=ATG;rbs_motif=AGGAGG;rbs_spacer=5-10bp;gc_cont=0.599

MSALGAAARKVTAPVVLRGAMIVSGAGIAAITGHEGLKYVGYLDSVGVP

>ERZ1030068.402-NODE-402-length-17889-cov-5.129920||full_1 # 1 # 1179 # 1 # ID=171681_1;partial=10;start_type=Edge;rbs_motif=None;rbs_spacer=None;gc_cont=0.702

QALRRERGEGLPEAVAAHLQLGRERDLGAWAGLAWTLAEAWCDTDPTVAGDLRGCADLAE

AARSFFRELTGTRVRYRGDSDPRADGDLAQLVQRSAYPDRYQRHAPYVAEVLARVIEPST

PGGNPVAWTGDPVWLETVLREALGDRLRTLPGWQDRGHGDFKDIRGVMWHHTGNSAEKPE

SIAKGRPDLAGPLANIHIAPNGIVTIVAVGVCWHAGEGSYPWLPTNNANWHMIGVECAWP

DIAADGSYDPGQRWPDAQIISMRDTAAALSLKLGVPADHNIGHKDYAGAAQGKWDPGNLD

MKWFQGEVDKDMRGLFDDHTEPPVVLPPANPPILKPPPLPFTDRELWEEILRQQRGPALR

GWEQLDNMTVVDYLAQLGRKVDAVELALAAKR

>ERZ1030112.3-NODE-3-length-57010-cov-12.224317_25 # 12161 # 12682 # -1 # ID=198812_25;partial=00;start_type=ATG;rbs_motif=None;rbs_spacer=None;gc_cont=0.651

MNRQRTLVAALAISLAGFGAWQASEGYSPTPYIPTAGDVPTIGHGSTRYEDGTPVKLTDP

PITRERAEQLARNLMAKDEQLFRASMPGVKLYQAEYDLYLDFVGQYGIGNWRQSSMRRHL

QAGEYRAACDALLKWRFQAGRDCKLPQNWGPKGCRGVWTRQQERHAKCIAAQE

>NODE_85_length_44781_cov_7.504270||full_3 # 1373 # 2107 # 1 # ID=227544_3;partial=00;start_type=ATG;rbs_motif=None;rbs_spacer=None;gc_cont=0.634

MTLYMYWPQVIWAVMVLLGLGIKLARHGQARTGRHSFWWQLFGSATVALLLWSGGFFSQA

RAAQPPQASLQYRADVIRNARLEWGMSAPVADFAAQLHQESGWRPDAVSPVGAQGMAQFM

PATADWISQLVPGLNSREPFNPAWAIRALVSYDRWLWQRVSAANSCERMAMTLSGYNGGL

GWVQRDKRMAVQNGLDGTRWFEHVATMNAGRSAANWRENRHYPQRILHELAPRYLTWGGG

SCVD

>NODE_821_length_13149_cov_3.602261||full_10 # 6830 # 7297 # -1 # ID=227570_10;partial=00;start_type=ATG;rbs_motif=GGAGG;rbs_spacer=5-10bp;gc_cont=0.630

MSLRNKIAIGALTGVLGMTGAVVAWFEGRSLVAYLDPVGIPTICEGVTKGVKLGDRATHE

QCDELLQRELRIALTAVDRQVRVPLPDTRRAALGSFVYNVGESQFASSTLLRLLNAGDAR

GACAQLSRWIYAGGKQLAGLVNRRAAERELCEVGL

>ERZ1023282.42184-NODE-42185-length-803-cov-1.733957_2 # 364 # 645 # 1 # ID=253226_2;partial=00;start_type=ATG;rbs_motif=3Base/5BMM;rbs_spacer=13-15bp;gc_cont=0.624

MTTRQDILNTAAAEVGYSRWDDPEAGSKYGRWYAQYKGAYFGASGVPFCDMFVSWVLFQS

GVNWLSAYVPGREAEARQRGVLISKWDVKPASP

>NODE_1834_length_5921_cov_5.585066||full_4 # 889 # 1398 # 1 # ID=259420_4;partial=00;start_type=ATG;rbs_motif=AGGA;rbs_spacer=5-10bp;gc_cont=0.657

MNSTAKKCAVLTIVALAALLPEFRTLRISSDGLLLLANAEGCRTSPYQCSAGVWTNGIGH

TRGVTPLSAVNERQVAVNLIDDLQRVERGIARCMPVSMPQPVYDATVSFAFNVGVGAACG

STFATLINQQKWRAACSQLPRWIYVDGKSSQGLMQRRRAEQAWCLKGAE

>ERZ1023194.156544-NODE-156545-length-583-cov-1.115530_1 # 3 # 326 # 1 # ID=309937_1;partial=10;start_type=Edge;rbs_motif=None;rbs_spacer=None;gc_cont=0.636

IKQQMFDTDVAHTTKDGPMLADSATRRAYAFASLTMFLFFASWGIWWSFFQIWLTSPSVG

LELNGQQVGTVGMTGRTFGPHLHFEVHGAGGAKLDPQAWLAERGIRV

>ERZ1023158.111391-NODE-111392-length-560-cov-0.831683_2 # 297 # 560 # 1 # ID=324941_2;partial=01;start_type=ATG;rbs_motif=None;rbs_spacer=None;gc_cont=0.617

MPYQPAPWTSKHTGKTYPTITQYDQKHLVLMVFFIMLCGILVVAVKKFQKANGLTVDGKA

GPATVKAILGKGDAAPAPAIPAEPVGGA

>ERZ1023158.18498-NODE-18499-length-1675-cov-1.489506||full_3 # 1054 # 1674 # 1 # ID=328582_3;partial=01;start_type=ATG;rbs_motif=GGA/GAG/AGG;rbs_spacer=11-12bp;gc_cont=0.702

MRSAKLVVGIVIGIILLSLVVVVILGDGSDDRCRPNGAITGGGGVPAGELSLPIAEGDYV

VSSTYKSADRPGHRGIDLAAADGTPMYAMAPGVVTAAGPASGFGNWIVIDHTIDGQPYST

VYGHMWDDGVDVQVGDTVAAGQHIGEVGNNGQSTGAHLHLEVWQGGRLTGGSETDPQPWL

DRAANPGSGGSEPPTDESTTSRQPSPR

>ERZ1029968.3438-NODE-3438-length-1584-cov-2.954872||full_2 # 928 # 1461 # -1 # ID=333244_2;partial=01;start_type=Edge;rbs_motif=None;rbs_spacer=None;gc_cont=0.373

MTKVVTYTCLLIAVMLVYVVVQSNGNTAKATAKVTHTEAMDYAESLEGKGWDYDNEYGWQ

CFDLVNMQWDYLFGHGLEGDYAKDIPTENNFDGEATVHKSTEGFKAQKGDIVVFNENYGG

GAGHTAIVTNGNEDGNYQKFESLDQNWEGKGAEKEEVAHRVVHDYESEMWFIRPEYK

>NODE_1392_length_8381_cov_7.169109||full_8 # 8016 # 8222 # 1 # ID=337460_8;partial=01;start_type=ATG;rbs_motif=AGGA;rbs_spacer=5-10bp;gc_cont=0.420

MSKTKFWVIGLAASAAFFTSLIKYEGYEAKPYLDSAKVATIGIGSTSYENGLCTRQISQN

PYPIRVLPS

>NODE_6322_length_870_cov_3.402454_1 # 182 # 775 # 1 # ID=347997_1;partial=00;start_type=ATG;rbs_motif=GGAG/GAGG;rbs_spacer=5-10bp;gc_cont=0.660

MKTSDRGVALIKAHEGLRLTAYTDPVGVWTIGYGHTTAAGPPKVERGMKITDAGADAILR

QDLAKFEGYVSSAVKVPLNQNEFDALVSWTFNLGPGNLRSSTLLKKLNAGDRAGAADEFL

KWTKAGGKTLPGLVKRREAERALFRTPIADLRPASKPVPVIDTIPATATASSSPPTLTLI

ALLVFVALLAVAIITKG

>ERZ1031260.1447-NODE-1447-length-2225-cov-4.057143||full_2 # 423 # 1118 # 1 # ID=353756_2;partial=00;start_type=ATG;rbs_motif=None;rbs_spacer=None;gc_cont=0.642

MNRRLLSTAVAAVLALTAAAAPQAGAVTLHVNGVPTDNGQNALKALADAANTIATSGATL

SYAGYSVVYDPRSLGEEVAEAFAPELPEDWVSIQEGSTADGRKVVTPTEGRFTSSFGPRW

GRLHNGIDIANSIGTPIYAVMDGTVVNAGPAQGFGNWVVIKHDGGEVSVYGHMRDYNVSV

GQRVSAGEQIAKIGNEGQSTGPHLHFEIKPDGVNPADPVAWFSARGISIPG

>ERZ1023368.112633-NODE-112634-length-525-cov-1.448936_1 # 3 # 524 # -1 # ID=374950_1;partial=01;start_type=Edge;rbs_motif=None;rbs_spacer=None;gc_cont=0.398

VLIVACPCALGLATPMSLMVGIGKGAQNVVTPPTSNDNVGVAYINGSNVNLRKGPDTGYG

VIRQLGKGESYKVFGQTNGWLNLGGDQWVYNDPSYIRYTGGNVPATSQSSNDGVGVVTII

ADVLRVRTGPGTNYGIVKNVYQGEKYQSFGYKDGWYNVGGNQWVSGEYVTFVK

>ERZ1023274.4257-NODE-4258-length-2501-cov-2.104661||full_4 # 2056 # 2499 # 1 # ID=387561_4;partial=01;start_type=ATG;rbs_motif=GGA/GAG/AGG;rbs_spacer=5-10bp;gc_cont=0.714

MKKLLALAVALIFLGPSTALIGVGVLMNPAATAACLPGSLIVGQIPDSLTATTRAGATVT

LNKTQLTHAATIITVGARTAGVGRPGAVIALMAALTESSLRMLANTSAYPESATYPNDGN

GGDHDSLGLFQMRPASGWGTVAELMDPT

>ERZ1030821.4-NODE-4-length-43429-cov-19.183313_39 # 22643 # 23146 # -1 # ID=387955_39;partial=00;start_type=ATG;rbs_motif=AGGA;rbs_spacer=5-10bp;gc_cont=0.565

MKSRIIAVVGASLVSIAGLLHIQNSEGVRYAAYPDPATGGAPWTICWGHTGPEVVKGLTV

TRKQCELWLNVDIREHEGYVRKLVKVPVKQGEYDALVSFSYNVGPTNLANSTLLRKLNEG

DRIGSCKEYSKWIYADKRIFQGLVTRRYNEQAMCLKLGDYVYDPRVR

>ERZ501044.614-NODE-614-length-6331-cov-4.874602_12 # 5638 # 6228 # -1 # ID=390429_12;partial=00;start_type=ATG;rbs_motif=GGAG/GAGG;rbs_spacer=5-10bp;gc_cont=0.658

MKTSDRGVALIKAHEGLRLQAYQDPVGVWTIGYGHTTAAGPPKVERGMKITDAGADAILR

QDLAKFEGYVSSAVKVPLNQNEFDALVSFTFNLGPGNLRSSTLLKKLNAGDRAGAADEFL

KWTKAGGKTLPGLVKRREAERALFRAPDASVPRPAPKPVPVIDTIPATAPSSPPTLTLIA

LVVFVALLAVAIITKG

>ERZ1030211.5868-NODE-5868-length-2001-cov-1.654162||full_3 # 1440 # 1916 # 1 # ID=415520_3;partial=01;start_type=ATG;rbs_motif=TAA;rbs_spacer=11bp;gc_cont=0.470

MYKRLHFKQLAFWGCVTLLAVASHSHAGQIYVYKDNNGSTLLTNRKSSDQSLKKVKVTYY

PDSNIHRYSNWGSSEASVLPSYSRNKNAFDHIIQQAAQRHGISSGLIKAVMHTESGFNVN

ARSPVGAQGLMQLMPATAKRFQVSNAYDPYENIMAGAKY

>ERZ1031287.24-NODE-24-length-83453-cov-15.193758||0_partial_24 # 20825 # 21352 # -1 # ID=421489_24;partial=00;start_type=ATG;rbs_motif=GGAG/GAGG;rbs_spacer=5-10bp;gc_cont=0.602

MALRKKAGAAAICAVSAIIAIVLANGEVRTNQRGLELIGNAEACRRSPYICPAGVLTDGI

GNTHGVVAGSVKTDAQIAADWQNNILDAENCVNRYASGRRLPDNAFSAATSVTFRAGCGN

LRGSQLFRLLRSGQLELACQQFPRWIYGGGRVLPGLVARAEKEQALCLDGLSLAG

>ERZ1029860.36-NODE-36-length-24596-cov-4.320443||full_31 # 18284 # 18961 # 1 # ID=433256_31;partial=00;start_type=ATG;rbs_motif=None;rbs_spacer=None;gc_cont=0.507

MKRIPTNNGYWLLNPCVFFVSALILIFSLVSCKPAAAETIPNAAKQHRAVLVRSAHMVWG

LDAPIATFAAQVHQESRWNVYAKSPVGAEGLAQFMPTTTEWIAAAYPKHLATAQPYNPGW

AMRALVQYDLYLHKRNQARSQCDHWAMILSAYNGGQGWVNRDRRLALASGASELAWFNSI

EKFNAGRSAANFKENRHYPRAILFKWEPLYVGSGWGNGVCAGFKF

>NODE_1731_length_3503_cov_3.983469||full_1 # 1 # 327 # -1 # ID=446048_1;partial=10;start_type=ATG;rbs_motif=GGAGG;rbs_spacer=5-10bp;gc_cont=0.645

MGKVLIKRGTSAAGAIAIILGGIYAAEGGYVNNKADRGGPTNLGVTERVAREHGYLGDMR

DFPKHCYGKITVCGDKIYVGTYIDKPGFRPLLAMDPGVAAVSYTHLRAH

>NODE_3131_length_2112_cov_3.816723||full_5 # 1615 # 2058 # 1 # ID=446095_5;partial=01;start_type=ATG;rbs_motif=GGA/GAG/AGG;rbs_spacer=5-10bp;gc_cont=0.581

MNPSTVKRCLVGAVLAIAATLPGFQQLHTSVEGLKLIADYEGCRLQPYQCDAGVWTDGIG

NTSGVVPGKTITERQAAGSFITNVLRVEKALDRCVLVSVPQNVYDALVSLAFNVGTGNAC

GSTMVKFINQKRWRCLLYTSDAADDLLC

>ERZ1059431.83-NODE-83-length-34232-cov-46.477134||full_28 # 22342 # 22980 # -1 # ID=460754_28;partial=00;start_type=ATG;rbs_motif=None;rbs_spacer=None;gc_cont=0.624

MRRSRSLLYIAAACVGLLVCLAASPPAKAEIPDQADRYRRDLTRIAQSEWGLDAPVSTFA

AQIHQESRWKFDAKSPVGAQGLGQVMPSTATWLAELFPKVLGKVEPYNPVWSMQALISYD

RWLANRIQARGPCEQGALILSAYNGGLGWVIKDRKLASAKGADPLTWFNSVERFNNGRSA

AAFKENRQYPRLILLRWEALYVADGWGQGVCQ

>ERZ1023267.3272-NODE-3273-length-3196-cov-2.848774||full_1 # 1 # 351 # -1 # ID=495038_1;partial=10;start_type=ATG;rbs_motif=GGAG/GAGG;rbs_spacer=5-10bp;gc_cont=0.595

MANHDTLKKGGAGALVGAALASAIALVAPWEGKRNNAYLDIVDVPTICYGHTGDDVKLGQ

TLSDAQCSKLLGEDLQEANDAVNRCVKVPLKDNQRAAFVSFTYNLGGGAFCKSSLLA

>NODE_2672_length_4075_cov_12.514179||full_5 # 1949 # 2458 # -1 # ID=500611_5;partial=00;start_type=ATG;rbs_motif=TAA;rbs_spacer=12bp;gc_cont=0.422

MSNKTKYIAATLVASAAFFTSLIGYEGYSSKPYKDTGGVATIGIGSTKYEDGTSVKITDK

PIDQKRAVQIAQAHISKDEQVFRKSLQGVKLSQAEYDLYLDFMYNFGQSNWRSSSMLTNL

KAGQYIAACKSLLKWKYVAKRDCSIRSNNCYGVWTRQVDRYNKCMGANS

>ERZ1023485.84-NODE-84-length-25011-cov-17.772840||full_26 # 18736 # 19269 # -1 # ID=518312_26;partial=00;start_type=ATG;rbs_motif=None;rbs_spacer=None;gc_cont=0.504

MNTKIRYGLSAAVLALIGAGASAPQILDQFLDEKEGNHTMAYRDGSGIWTICRGATVVDG

KTVFPNMKLSKEKCDQVNAIERDKALAWVERNIKVPLTEPQKAGIASFCPYNIGPGKCFP

STFYKRLNAGDRKGACEAIRWWIKDGGRDCRIRSNNCYGQVIRRDQESALTCWGIEQ

>ERZ1023485.90-NODE-90-length-21889-cov-16.808830||full_23 # 15905 # 16438 # -1 # ID=518313_23;partial=00;start_type=ATG;rbs_motif=None;rbs_spacer=None;gc_cont=0.547

MNTKIKYGLSAAVLALIAAGAPAPDILDQFLDEKEGNHTTAYRDGAGIWTICRGAIMVDG

KPVVPGMKLSKEKCDQVNAIERDKALAWVEKNIRVPLTEPQKAGIASFCPYNIGPGKCFP

STFYRRINAGDRKGACEAIRWWIKDGGRDCRIRSNNCYGQVSRRDQESALACWGIDR

>ERZ501066.4335-NODE-4335-length-525-cov-0.761702_1 # 8 # 523 # -1 # ID=548612_1;partial=01;start_type=Edge;rbs_motif=None;rbs_spacer=None;gc_cont=0.657

SNYRQFTAWARALDPQAPDFVVNPDAVLTDPWEGLGPIWYWDTRDLNRYADDGDLLTITK

RINGGTNGLADREAQYTRAGLVLLGFTTIKQFQDHVGLKVDGIAGPATRAAIHAELLKRP

LVTFGTARPTPKPVPVIDTIPPAASSSPPTLTLIALVVFVALLAVAIITKG

>ERZ1029868.3834-NODE-3834-length-1986-cov-10.267737||full_2 # 506 # 1261 # -1 # ID=549877_2;partial=00;start_type=ATG;rbs_motif=None;rbs_spacer=None;gc_cont=0.516

MKASNFINKNGLVDVEKYSAYMAGLRKRLLWRFIAELIFGASVFIVLVFFFIASAPSCAQ

EVPQSAAAYKRTLIRSAHGHWGLDAPVSLFAAQIHQESGWRIDARSPAGAEGLAQFMPAT

SEWFSTLHPRDLTTAQPYNPAWAMRALVLYNQYLYRRVDARDICQRWAFTLSAYNGGLGW

VNRDKALAQASGVDPLIWFDATEDYNAGRGNAAFAENRHYPKAIIYRHQPLYVAAGWGTS

VCGVGGNSAHF

>NODE_4893_length_741_cov_1.626822_1 # 2 # 469 # -1 # ID=555529_1;partial=10;start_type=GTG;rbs_motif=None;rbs_spacer=None;gc_cont=0.675

MKKLAVIATVLVLFSPALLLLGVGLMFSPGANASCTTSTSGPSVVGPVPDSLEVTTADGQ

TFTLNTTQLTHAATIIQTGSQIEGVTRDGLQIALMAALTESNLRMLANTSVYPDSGNHPN

DGDASDHDSLGLFQMRPQAGWGTVAELMDPVYQAEA

>NODE_22203_length_512_cov_1.973742_1 # 27 # 512 # -1 # ID=605019_1;partial=01;start_type=Edge;rbs_motif=None;rbs_spacer=None;gc_cont=0.451

TDPWPPGMLDVYYRIVASILWYLGLDASRCISHWEYSLVAQGKWDPGAGDGVVGHLMDMK

KFRSHVQYYIQNPPFKKEEEDDLSSEFDKQYKSRYPGSNFKGSLRDFILNSDAHSFASRV

NTEKLLKLQEENNKLLQDNNRINKELAEVLSKLAEAYRANN

>ERZ1030134.1439-NODE-1439-length-4241-cov-4.478500||full_7 # 2431 # 3039 # 1 # ID=639434_7;partial=00;start_type=ATG;rbs_motif=3Base/5BMM;rbs_spacer=13-15bp;gc_cont=0.635

MTAQPGKVWASLGAAILAIATGVFVVEGGYSNNPADPGGETNHGVTERVARDHGYDGPMR

DLPKETAQQIYVGSYVEGPGFHRVVAISPAVGEKLVDVGVNAGTGRSARWFQTALNQLSR

GGTDFPMVAVDGQIGPQTLNAYRALERKRGRIKACELVLKLLDAQQGTHYMSLNKPTFIV

GWADNRLGNVPPARCADSVAPP

>ERZ1029988.2127-NODE-2127-length-2778-cov-5.222549||full_1 # 1 # 360 # 1 # ID=646204_1;partial=10;start_type=Edge;rbs_motif=None;rbs_spacer=None;gc_cont=0.683

FRYMEEIASGAAYEGRVSLGNTQPGDGKRYKGRGPIQLTGRANYRAFGREVGIDFEAHPE

IVAFPSIGLMAAVRYWNSRGLNAKADVDDLLGITRAINGGTNGLDDRKARTAKAKELIL

>ERZ1030888.7458-NODE-7458-length-503-cov-1.558036_1 # 1 # 204 # 1 # ID=647108_1;partial=10;start_type=Edge;rbs_motif=None;rbs_spacer=None;gc_cont=0.426

VTESVLRGDEGWKPEDSGQHKRLAFVSAGGLIVELGFITNFTEMKVLMEKRWLVAKAIAE

AIREEYK

>ERZ1030151.151-NODE-151-length-35172-cov-9.111257||full_12 # 3447 # 4217 # -1 # ID=695174_12;partial=00;start_type=ATG;rbs_motif=AGGA;rbs_spacer=5-10bp;gc_cont=0.678

MAKGNFPACLAVTLPHEGGWADHPKDPGGATMKGITLATFRQYYPDASKADLRAISDKDV

ERIYRVGYWGPIRGEQLPAGVDLAVFDYGVNSGPSRAAKALQASVGASVDGKIGLETIGA

TDKRDPVATVKAVCARRLSFLRGLSTFSTFGKGWSRRVADVEAKGVAMAGAGAAVLRAEA

DKAAKASNDQAKVATGTGAAGAGGGGFEIASGDVNWLLVFGVIVVAMIVVSLVKSRSNIN

KDRAVAYSAVARQVSE

>ERZ1029956.13909-NODE-13909-length-1156-cov-4.699364_1 # 2 # 469 # 1 # ID=696672_1;partial=10;start_type=Edge;rbs_motif=None;rbs_spacer=None;gc_cont=0.665

SPVAPDFVRDPDAVNTDPWEGLGPIWYWDTRNLNRYADAGDFETVTRRINGGLNGYADRQ

ARYARAALVLMGRDPNGIKAYQAAVGLKADGIVGPVTVASLHRALTSMEPVRFADAARPV

PKPNPAPARGTPAGGGVIAVVVAAIAAVAAFIGLR

>ERZ1029956.25-NODE-25-length-60096-cov-64.413284||full_53 # 36694 # 37215 # 1 # ID=697618_53;partial=00;start_type=ATG;rbs_motif=None;rbs_spacer=None;gc_cont=0.634

MNRARTWVGALAMSLAAFATWQASEGFTPVPMIPTQGDVPTIGYGSTRYEDGTPVTLADP

PITRDRAEHLARALHDEEERRFRASLPGVKLFQEEYDLYLDFSGQYGSANWRKSSMRRHL

LAGEYAKACDALLMWRKAGGYDCSTLVNGKPNRRCWGVWVRQLERHAQCWSVQ

>ERZ1023456.349-NODE-349-length-5150-cov-4.100294_3 # 781 # 1290 # -1 # ID=700643_3;partial=00;start_type=TTG;rbs_motif=AGGAG;rbs_spacer=5-10bp;gc_cont=0.655

MSQTAKRCAVAAVLAIAALLPQFKTLKTSEAGLRLIADAEGCRTSPYQCSAGVWTNGIGH

TAGVTPQSVISERQAAVNLVYDVMRVERAIDACMRHDMPQPVYDAVVSWAFNVGTYAACR

STLGAYINRGEWRSACLQLPRWVFVKGVFSQGLQNRRDRELAWCLKGAA

>ERZ1023456.135-NODE-135-length-12730-cov-5.666509||full_10 # 6045 # 6557 # -1 # ID=700668_10;partial=00;start_type=ATG;rbs_motif=None;rbs_spacer=None;gc_cont=0.647

MNLQTVKRCTVGVVLAVAATLPGFQQLHTSLEGLKLIADFEGCRLQPYQCSAGKWTDGIG

NTHGVVPGKTITERQAAESLITNVLRTEAALGRCIVTQLPQHVYDAVVSFAFNVGTGNAC

GSTLVKLLNQRRWADACRQLPRWVYVNGVFNQGLDNRRGREMAWCLKGAA

>NODE_24890_length_708_cov_1.186830_1 # 175 # 708 # 1 # ID=789567_1;partial=01;start_type=ATG;rbs_motif=None;rbs_spacer=None;gc_cont=0.373

MKSIRTKLIAAFVAAGLSAPSAFVAYDLTYPSESLSKTVYLDPIGYPTVCIGRMDKSLQL

GQEFSIDECMKMFASDWKKHQNQLDSVVKVPYKSEWQKEALTDFTFNVGIGNVKSSKLLS

LLNQGKHVEACQQLSRWVKAKGKTLRGLVIRRDKTMPYCLGELPWDKQQAYKEFEEEY

>ERZ782894.5244-NODE-5244-length-1593-cov-3.302341||full_2 # 261 # 662 # -1 # ID=795402_2;partial=00;start_type=ATG;rbs_motif=AGGAG;rbs_spacer=5-10bp;gc_cont=0.704

MRIPARRTLLAAPALAAALVAAGGSVASAAEGTCPTPLKPLRAPALGTVTLRRGSRGSAV

RSLQNLLNHAKRLRTSNGTWYTPCPLVEDGVFGSGTEKRLRDGQRAFGVTADGVVGPTTR

RAALEAYRNGDIR

>ERZ941563.77-NODE-77-length-41991-cov-5.891430_40 # 25822 # 26379 # -1 # ID=802657_40;partial=00;start_type=ATG;rbs_motif=AGGAG;rbs_spacer=5-10bp;gc_cont=0.665

MTNRIAKSAAAVAAAVSVAGVGFIAGWEGKENKPYQDIVGVWTVCYGSTGAHVRSGGVRT

DEQCLTMLEEDLVRFEAAVNRCTPAPKNQNQFDALVSLSYNIGERAYCGSTLARKFNAGD

VAGASAEFPKWSYAGGKQVRGLLNRRLAEQRLFNTPVSPAPEAPVAPSGTVRYNPPGSGV

GLVQR

>ERZ941563.6781-NODE-6782-length-1800-cov-2.245272||full_5 # 1288 # 1800 # 1 # ID=815115_5;partial=01;start_type=ATG;rbs_motif=ATA;rbs_spacer=4bp;gc_cont=0.505

MTKTVTITAGHGGGDPGAVNGNITEAYIATDMRNMLKLYLERAGVKVRTDGDGNENQSLR

QALRLIPGSDLAIEIHCNAASTSQAGGVEALAQPKDKAICQKLCSAISDVMSIPVRGNAG

GWKDQSSGQHSRLAYVSGGGIILELFFISNPKELAIYQSKKWLVARELADV

>ERZ1029882.4468-NODE-4468-length-2389-cov-1.592973||full_2 # 1311 # 1931 # 1 # ID=831983_2;partial=00;start_type=ATG;rbs_motif=3Base/5BMM;rbs_spacer=13-15bp;gc_cont=0.678

MSLSSTLERPDVLSAPGGGDDDENGSRKKIIIWVVALILVPVVIVAGYTMFLMMAIAGAS

GGGSSQCTAGSLTTDKLEVQTTGGSTRTLGATELGHAATILSVARSLGVSARGQQIAIMT

ALQESGLKMYANSTVPASLDYPHDAVGSDHDSVNFFQQRVSGWGTVKDLMDPTYAAKAFF

GGPEGPNHGSPRGLLDIPGWESMSLG

>ERZ1030060.2411-NODE-2411-length-2235-cov-3.882569||full_2 # 661 # 1800 # -1 # ID=853685_2;partial=00;start_type=ATG;rbs_motif=AGGAG;rbs_spacer=5-10bp;gc_cont=0.446

MKKKFLVGAIVALFLLPIFPTSVDAAKGDQGTDVAVYQGAQGRFGYAHDKFTIAQIGGYN

AAGLYDQWTYPTQVSSAIAQGKRAHTYIWYDTWGSMSIAKTTMDYFLPKIQTPKNSIVAL

DFEHGASSNMQANTDTILYGMRRIKQAGYTPMYYSYKPFTLSNVYYKQILAEFPNSLWMA

AYPNYNVTPSPVWSVFPSMDGVAIYQFTSTYVAGGLDGNIDLTGITDNGYGGAIKDDDGK

VTVDPETSTPAIDDGQNANETAKKEIKAGYTVKVNFSAKNWATGQAIPQWVKGNSYTVQQ

VSGTKVLLGGIMSWINRKDVEILQTNTTNPAPSTNVHVVQSGETLSGIAAKLGTTYQALA

QKNGLSNPNLIYPGQQLAY

>NODE_8641_length_1824_cov_3.141323_3 # 502 # 1791 # 1 # ID=8214_3;partial=00;start_type=ATG;rbs_motif=AAAA;rbs_spacer=3bp;gc_cont=0.401

MKKLIKKAAIGMVAFFVVTASGPVFAAVGDQGVDWSKYNGDYGNFGYDHDKFAFSQIGGT

YGGSFVDQATYSTQVASAIAQGKRAHTYIWYQVGGSQEVAKAALDRYLPKIQTPKNSIVA

LDYEGGASGDKQANTDAILYGMRRVKAAGYTPMYYSYKPYTLENVNYKQIIKEFPNSLWI

AAYPNYEVTPVPNYSFFPSMDGISVFQFTSTYVAGGLDGNVDLTGITDNGYGEQKGQEVK

PDTITPAIDSGKEANEVKGNDVEVGMTVKVNFSATNYATGETIPQWVKGKPHKIIQKNGD

TVLLDGIMSWLSVHDVETIDASTSQPTTSAKTHIVQSGDTLSGIASNWGTNWQELARQNS

LSNPNMIYSGQVIHFTGGQSGATARTYTVRSGDNLSSIASRLGTSVQSLVSMNGISNPNL

IYAGQTLNY

>NODE_57_length_65793_cov_10.251514||full_67 # 54227 # 54763 # 1 # ID=9868_67;partial=00;start_type=ATG;rbs_motif=GGA/GAG/AGG;rbs_spacer=5-10bp;gc_cont=0.553

MSNKAKFSAAMLALLAAGASAPVLFDQFISEKEGNALVAVVDPGGVWSLCHGVTVIDGKR

VIKGQRATEEQCRKVNAIERDKALAWVEKNVHVPLTPPQKVGIASFCPYNIGPGKCFPST

FYRKLNAGDRKGACAEIRRWVFDGGRDCRLTKGQKNGCYGQVDRRDQESTLTCWGLYE

>ERZ1029884.12-NODE-12-length-64869-cov-97.923628||full_9 # 7207 # 7947 # 1 # ID=70852_9;partial=00;start_type=ATG;rbs_motif=GGA/GAG/AGG;rbs_spacer=5-10bp;gc_cont=0.656

MFNSTKQGGKHRKQSPNKGRVAVVAAATGAVSTAGFSGLVAGALTSNQHDEQDVSIKLAA

DADELQTSAPQAPAASTEETPQILAIAEYKPVENLAEQLDKAVQHSEEVARQDEAARAPL

FTKPAEGAYTSGFGPRWGTMHNGVDIANAPGTPILAVTGGTVIDSGPAQGYGNWIRIRHE

DGSISVYGHMQSLYVAVGETVQPGQLIAGMGSEGFSTGSHLHFEIWPDGATPIDPAPWLA

AHGINL

>ERZ1029884.12-NODE-12-length-64869-cov-97.923628||full_10 # 8251 # 8949 # 1 # ID=70852_10;partial=00;start_type=ATG;rbs_motif=GGA/GAG/AGG;rbs_spacer=5-10bp;gc_cont=0.652

MNRRITLSLAAVSAAAALALAMVPSPASALTITVGGKNISDAGQALGAVSKAAGAIKESG

ATITAGDYKVVYDPRALEAPLAAAFAPAQSSDWVAPQRGRTADGRTVVVPASGHYTSGFG

PRWGSVHQGIDIANSLGTPIYSVMDGTVIAAGPATGFGNWVVIKHDGGEVSVYGHMRHYD

VAVGQRVSAGQKIASIGSEGQSTGPHLHFEIKPDGQTQVDPVGWFAAQGIKI

>ERZ1023144.46250-NODE-46251-length-643-cov-1.051020_1 # 2 # 499 # -1 # ID=72682_1;partial=10;start_type=ATG;rbs_motif=AGGAG;rbs_spacer=5-10bp;gc_cont=0.349

MKTQSQINKRLRDYKNGVVDSPYRVKRWTSYDASFGAMEPGCIDKDRSYHAQCMDLAIDY

VMWLTDNQTEMWGDAKSSIINKFPKGWKIVENKPSTIPKKGWIAVYTAGTYSRYGHIGIV

YDGGNTNSFQILEQNWNEQTSCNLLGVTYIFLLNILNYFFKNPFSM

>ERZ1029952.1299-NODE-1299-length-3769-cov-7.568121_7 # 2621 # 3175 # 1 # ID=186766_7;partial=00;start_type=ATG;rbs_motif=AGGAGG;rbs_spacer=3-4bp;gc_cont=0.557

MLKVARKIVTPVVLRGAMVVSGAGIAAITGHEGLKYVGYLDSVGVPTVCYGHTRTAKVGV

EYTQEQCEQLLQEDLAEFSATVNKYITFPLSQPQFDALVSFCYNVGSYACRTSTMFRLIN

DGDYLGGAAQFDRWYRAGGLDCRDRANNCYGVWTRRQAEKKLFLSGTSQHNAPLIPIVAS

GGNP

>ERZ1023377.2821-NODE-2822-length-4187-cov-2.696999_1 # 1 # 228 # -1 # ID=188158_1;partial=10;start_type=ATG;rbs_motif=GGAG/GAGG;rbs_spacer=5-10bp;gc_cont=0.482

MAMSPKLRNSVIAAIGGGAIAIASSLITGPTGNDGLEGVRYKPYRDVVGIQTVCYGHTGK

DIMLGKTYTETECKAL

>ERZ501047.7328-NODE-7328-length-1029-cov-2.553388_1 # 1 # 264 # -1 # ID=331207_1;partial=10;start_type=ATG;rbs_motif=None;rbs_spacer=None;gc_cont=0.644

MNAHRALLTILALSTTASAATYYTVQPGDSLATVAQKANMEPATVLKLNGLQNPTLQVGQ

KLNLTPSGFARIEAAQQAPTQTVRSEEQ

>ERZ501046.4658-NODE-4658-length-794-cov-1.485792_1 # 3 # 494 # 1 # ID=332129_1;partial=10;start_type=Edge;rbs_motif=None;rbs_spacer=None;gc_cont=0.425

KHYQSLGSTVQEMLDNAGNQEGWKKNATGWWYVNADGSYPTNKWQKINNAWYYFDSNGYM

KVNSWHKHSDGYWYYLLPNGSMATGWVLISNKWYYFKEDGKMATGWVKYKEHWYYLDAKD

GDMESNQFVKSADGTGWYYLKPDGSMADKPEFTVEPDGLITTK

>ERZ1031260.564-NODE-564-length-3579-cov-7.534052||full_2 # 558 # 1253 # -1 # ID=353739_2;partial=00;start_type=ATG;rbs_motif=None;rbs_spacer=None;gc_cont=0.614

MNRRLLSTALAAVLVLTAAAAPHAGAVTLHVNGVPTDNGQQTLKALADAANTIVTSGATL

SYAGYSVVYDPRSLGDEVAEAFAPELPENWVSSQEGSTSDGRKVVTPTEGTFTSGFGSRW

GTLHNGIDIANSIGTPIYAVMDGTVINAGPAQGFGNWVVIRHDDGEVSVYGHMRDYNVSV

GQRVSAGEQIAKIGNEGQSTGPHLHFEIKPDGVTQADPVAWFNARGISIPS

>ERZ1029922.2-NODE-2-length-252270-cov-173.370803_186 # 196106 # 196888 # -1 # ID=392185_186;partial=00;start_type=TTG;rbs_motif=AGGAG;rbs_spacer=5-10bp;gc_cont=0.591

MGKHGKSAPGSNRGSAKFISAPIAAIAVTAVVGSAGMTAMNMANGDSQISSGDHTKVMAA

AMDGKGYVSDADLVEGENNGVGGGQPTVGVPQILTGEFAASNAPITNYDEQLRKAVEFSN

ERAEADRLARMPSTVVPADGAYTSGFGMRWGTNHNGIDIANAIGTPIYSAMDGVVVDAGE

AQGYGQWVRVLHGDGSITVYGHIETIDCYVGQKVMAGEKIAGMGNRGFSTGPHLHFEIWP

DGANPVDPVPWLQERGVNFG

>ERZ782888.1895-NODE-1895-length-3020-cov-3.023609||full_2 # 2170 # 2934 # -1 # ID=443760_2;partial=01;start_type=Edge;rbs_motif=None;rbs_spacer=None;gc_cont=0.612

MKRNLRARKTVALTALAVCTTLATTMGATASAAQPAASVDVSDGSSQPEVSTNADNATLI

DAFVGLTKSTAGVLAGAATPEVSNEGGALNIVLDPSSVPGLAQAFAPSGNHSGLKPTKSQ

DSAGNTVVFPTSGTLTSGFGPRWGAFHSGIDIANPIGTPIYSIMDGEVISSGPAQGFGHW

IRIQHDDGTISVYGHMPGDQLLVNVGDRVSAGQQISVIGNEGQSTGPHLHFEVHPGGGAA

VDPVNWFAQRGINI

>NODE_464_length_5689_cov_20.004970||full_4 # 4200 # 4958 # -1 # ID=475934_4;partial=00;start_type=ATG;rbs_motif=AGGA;rbs_spacer=5-10bp;gc_cont=0.626

MKRMNRARKNVALSIVTLGTVLATGTTAFAAEPVASVDVSDGSSEPTATNVDNAGVVDAL

VGLATTTVGVLNGEATPQVDGNESGGVNIVLDPGTVPGLKEAFAPQGNHSGLTTKRGTDS

AGNTVVFPTSGTLTSGFGPRWGTMHNGIDVANPVGTPIHAVMDGTVINSGPAQGFGNWIR

IQHDDGTISVYGHMPADLLKVNVGERVTAGQEIAGIGSEGHSTGPHLHFEIHPGGGAAVD

PVSWFNERGISV

>ERZ1030898.18-NODE-18-length-48693-cov-49.878017_18 # 19221 # 20189 # -1 # ID=510225_18;partial=00;start_type=ATG;rbs_motif=GGAG/GAGG;rbs_spacer=5-10bp;gc_cont=0.680

MARYVGRHRKHTTPAPVKAVAAAAVVAGVSGVGAGVATAQTKADPAPTTTLVPGPALDAD

QVARDAQAAVDGAVKSAKRDVDAATRGIANSLDVAKATMQKHGVAAQDPTAPQIMQGAVV

VMDPAVRESAAERGAQLQRSLDAAVHYNEQVEKQRAEEEAAAAAAEAAAKAQAAAEAAAA

QANAAAGVGSTVIKGGVALPASGAFTSGFGSRWGSFHSGVDIANSLGTPIYAAMAGTVID

SGPAQGYGQWIRIRHDNGAITVYGHMQTLGVSVGQRVAAGEYIAGMGSLGFSTGSHLHFE

IWPDGANAVDPQAWLAQHGIYL

>ERZ1030944.1719-NODE-1719-length-1516-cov-2.488706||lt2gene_2 # 932 # 1516 # 1 # ID=545263_2;partial=01;start_type=GTG;rbs_motif=GGAG/GAGG;rbs_spacer=5-10bp;gc_cont=0.631

MRLKQGLSAIVAVGTVVSGITVLTMQNPAPVEAVPMVRPSPELIPVEPPSETPEPDAAAT

ESANSAGPKKPIFPVHQYPEDWYLPRPASGDQVPTDDVGYVNTEGNGTLATPVDGRRTSP

FGMRMHPVLHVYKLHTGLDFAAPCGTPVGAAADGVVSFVGWAGGNGYMVGIRHGKINGYN

VVTNYAHLSSACLLY

>ERZ1029904.29-NODE-29-length-48022-cov-39.599808_24 # 25920 # 26888 # -1 # ID=545778_24;partial=00;start_type=ATG;rbs_motif=GGAG/GAGG;rbs_spacer=5-10bp;gc_cont=0.684

MARYVGRHRKHTTPAPVKAVAAAAVVAGVSGIGAGVATAQTKPAPAPTTTLVPGPALDTD

QVARDAQAAVDGAVKSAKRDADAAARGIANSLDVAKATMQKHGAAAQDPTAPQITSGAVV

VMDQAARETVDERGAQLQRSLDAAVQYNEQVEKQRAEEEAAAAAAEAAAKAQAATEAAAA

QSNAAAGVGSTVIQGGVALPASGAFTSGFGSRWGSLHGGVDIANSLDTPIYAAMAGTVID

SGPAQGYGQWIRIRHDNGAITVYGHMQTLGVSVGQRVAAGEYIAGMGSLGFSTGSHLHFE

IWPDGATKADPQAWLAQYGIYL

>ERZ1029986.11873-NODE-11873-length-1066-cov-2.492582_1 # 3 # 284 # -1 # ID=638324_1;partial=10;start_type=ATG;rbs_motif=GGAGG;rbs_spacer=5-10bp;gc_cont=0.535

MAMSPKLRNSVLAAVGGGAIAIASALITGPTGNDGLEGVRYNPYQDVVGIWTVCYGHTGK

DIMLSKKYTEAECRALLSKDLNAVARQINPYIQK

>NODE_726_length_4255_cov_4.038095||full_3 # 3265 # 4254 # 1 # ID=705579_3;partial=01;start_type=ATG;rbs_motif=TAAAAA;rbs_spacer=14bp;gc_cont=0.368

MENNTFKLEPTLKTSTIVHSILKKTNRNIILAVFFLLVAMLSGSFTAYLIVEKDSASTEE

TLLVKLKQNKKDLKLLQSEYNKLSAKMKEAGKILATIEEKDAKIYRNVYGLEEIPEEVRM

AGFGGTDRYSDLLSLPNGEYVAETAKSMDVLVKRLEFQAKSLAEIKAASNGKEKEFASIP

AIQPIANKHMSRVASGFGMRFHPILKIKKMHKGLDFAAPTGTPIYATADGRVEKTGSENG

YGNMVKINHGNGYETLYGHMSKIKVKPGQRVKRGEIIGNVGNTGMSTGSHLHYEIHKNGE

VINPLTYFYKDISPDEFVKLYEESQKMSVS

>ERZ1023148.93313-NODE-93314-length-540-cov-1.000000_1 # 2 # 538 # 1 # ID=456528_1;partial=11;start_type=Edge;rbs_motif=None;rbs_spacer=None;gc_cont=0.663

QKNRTPTATRASTPSIRAWRAGRAAAAIVTVPVGVLKAGAVAFTPALPEAARTALDGLSM

GAYTAALPGFNSALVQAACTTVKRSAMYCAQVGHEAGGLYYQEEIWGPTRQQKKYDPSSG

SDLSRQLGNTKVGDGPLYKGRGRIQLTGKGNYERFSVWCFVRKLVPTRDYFVQHPQLVA
