# Supplementary material for: Exploration of Human Skin Phageome to Reveal Endolysins and Novel Antimicrobial Peptides for Therapeutic Applications
Source: Microbiologyopen. 2025 Nov 9;14(6):e70115. doi: 10.1002/mbo3.70115 (PMC12597775; doi:10.1002/mbo3.70115)
Supplement: Supplementary file 7 — Table S4: Endolysin‐derived peptides. [file MBO3-14-e70115-s007.docx]

**Table S4. Endolysin-derived peptides**

>Sequence_1

LGFFSHVVGNGRVMQVGPVN

>Sequence_2

QAGNYAAACKALLKWRFAAG

>Sequence_3

CKALLKWRFAAGYDCSTPGN

>Sequence_4

TPGNKRCWGVWQRQLNRHAK

>Sequence_5

VGQYGIGAWRTGSPRRDLLA

>Sequence_6

WRTGSPRRDLLAGNYAQACH

>Sequence_7

YRYAAGFDCSTPGNKRCRGV

>Sequence_8

CSTPGNKRCRGVWARQLDRH

>Sequence_9

TLWYHPTNYVEWHCGKPYPN

>Sequence_10

KNQLKLIDYFISRIKHYANG

>Sequence_11

YFISRIKHYANGGKTPKKPQ

>Sequence_12

KKPQISDKPFKKYNWSGKFR

>Sequence_13

PFKKYNWSGKFRAHKNNTLP

>Sequence_14

ANLWWIKFKYPRKGSSNKFF

>Sequence_15

KYPRKGSSNKFFYMPIGHIE

>Sequence_16

ADQFLAWKKAGGKVLPGLVR

>Sequence_17

KAGGKVLPGLVRRREAERAL

>Sequence_18

KNEQTALKFAAHKLKKWGLP

>Sequence_19

FAAHKLKKWGLPANRNTVRL

>Sequence_20

TPNTPIKTHYVGPIRSSPVS

>Sequence_21

FIRKYIAPNVQKYLKQAGHS

>Sequence_22

KVDKDINNCLKATVGTIRNI

>Sequence_23

GKPIGGTSAGSKKAKQTKWN

>Sequence_24

AGSKKAKQTKWNWKGRFIPN

>Sequence_25

TKWNWKGRFIPNTAIKVRTS

>Sequence_26

FIPNTAIKVRTSPSLKGKIV

>Sequence_27

DKKERIKAEKYWGKLKWNWH

>Sequence_28

HKALKAKKWATFAQGYNGPA

>Sequence_29

YGIGTWLKGSPRRDLLAGNY

>Sequence_30

NKRCAGVWARQLKRHAKCMS

>Sequence_31

DDGIIGRGTLTAVFKKLGAS

>Sequence_32

RFKGRGPIQLTGRTNYRKYG

>Sequence_33

GQKYRGRGLIQVTGRANYEA

>Sequence_34

PVYAALSAGWFWHRAGLNTL

>Sequence_35

GIIGAGTLTALFARMGAAPA

>Sequence_36

GKRYKGRGPIQLTGRANYRR

>Sequence_37

IGLLVGCVYWDGRKLNAKAD

>Sequence_38

GDAKSSIKNKFPKGWKIVEN

>Sequence_39

NKFPKGWKIVENKPSTIPKK

>Sequence_40

IPKKGWIAVYTAGTYSRYGH

>Sequence_41

FQILEQNWNGWANKKPSLRW

>Sequence_42

SLRWDNYYGLTHFIVPPIAK

>Sequence_43

GLTHFIVPPIAKEVEKSKKD

>Sequence_44

HIKGWNMTKRGRKPKGVVIH

>Sequence_45

TPIKTHYVGPFRSCPVSGVL

>Sequence_46

GPFRSCPVSGVLQPGQTIKY

>Sequence_47

PEGWKIVLNRPSTIPKKGWI

>Sequence_48

NRPSTIPKKGWIAVFTSGTY

>Sequence_49

PTSTTIKDKPGSASTPANRK

>Sequence_50

MVTMPVPKGFKVTSSFGPRW

>Sequence_51

GFKVTSSFGPRWGTTHWGTD

>Sequence_52

VLDWTRRFVFGKMRPTHQIK

>Sequence_53

VFGKMRPTHQIKNIIIHVTV

>Sequence_54

PPVKLSAADLRAGKKGFAGH

>Sequence_55

TTKYFTDFLKGFIGPIISDV

>Sequence_56

GYLDSVGVPTVCYGHTRTAK

>Sequence_57

QFDALVSFCYNVGSYACRTS

>Sequence_58

WYRAGGLDCRDRANNCYGVW

>Sequence_59

CRDRANNCYGVWTRRQAEKK

>Sequence_60

AKTYMAHDLKKFEATVNKAV

>Sequence_61

FDVWVNAGGKRMQGLVNRRA

>Sequence_62

MKKLIKKAAIGMVAFFVVTA

>Sequence_63

AIGMVAFFVVTASGPVFAAV

>Sequence_64

AILYGMRRVKAAGYTPMYYS

>Sequence_65

MSNKAKFSAAMLALLAAGAS

>Sequence_66

KNVHVPLTPPQKVGIASFCP

>Sequence_67

PPQKVGIASFCPYNIGPGKC

>Sequence_68

SFCPYNIGPGKCFPSTFYRK

>Sequence_69

PGKCFPSTFYRKLNAGDRKG

>Sequence_70

IRRWVFDGGRDCRLTKGQKN

>Sequence_71

GRDCRLTKGQKNGCYGQVDR

>Sequence_72

SSFGPLLPDLLKRFQVTTAL

>Sequence_73

EAKPELVATWPWAAWAVFYY

>Sequence_74

TWPWAAWAVFYYWSTRNLNA

>Sequence_75

RINGGTNGLSDRATYLGKAK

>Sequence_76

SVGVWTIGYGATRGVKAGMS

>Sequence_77

QFPRWNKAGGQVLPGLVRRR

>Sequence_78

LGVMPGARLRAGIFLTFLNA

>Sequence_79

LRAGIFLTFLNAAFAGHQIN

>Sequence_80

FLNAAFAGHQINTARRVAAF

>Sequence_81

HAYTRGQWLDPRVWPAVERA

>Sequence_82

LDPRVWPAVERAAILCRRLC

>Sequence_83

DLGKRVDALSWVKNPVTGKL

>Sequence_84

LSWVKNPVTGKLWRTKDALW

>Sequence_85

VDGWQTRGYAGQSLYGVYGH

>Sequence_86

CTYGRSDLPGPLCQLMLGRS

>Sequence_87

PGPLCQLMLGRSGTVYAVSY

>Sequence_88

FGRIQGVFVHHIGSDRYHWS

>Sequence_89

VATLVGAGIAWHAGKGWYKN

>Sequence_90

IAWHAGKGWYKNWPTNDANR

>Sequence_91

DAYYRICAAILWFLGKRATT

>Sequence_92

AILWFLGKRATTDTLLAHWE

>Sequence_93

LFLWPLILLVFAIGIFIVPK

>Sequence_94

AYRDTGGVWTIGFGTIRYPS

>Sequence_95

KPRPTHQIRNIIIHVTVNAP

>Sequence_96

GDVLTTKFFTDFLKGFVGPV

>Sequence_97

PQDSYVSSPFGPRWGTVHRG

>Sequence_98

PFGPRWGTVHRGTDFGRSGG

>Sequence_99

ACKREFEARGVRVIGCYSYV

>Sequence_100

RGVRVIGCYSYVPYWENRIR

>Sequence_101

WVAAYGTNPRGVPSAIYPGN

>Sequence_102

PRGVPSAIYPGNAHRQWGYP

>Sequence_103

YPGNAHRQWGYPLGNQRPVL

>Sequence_104

WGYPLGNQRPVLWQFGSNAL

>Sequence_105

SLSNLKGVHPDLIKVVKRAI

>Sequence_106

GAADQFPRWNKAGGKVLNGL

>Sequence_107

WNKAGGKVLNGLTKRRNAER

>Sequence_108

QPGDGRRFKGRGPIQVTGRR

>Sequence_109

KGRGPIQVTGRRNYTVLSQW

>Sequence_110

MTRVSPHFQRREFACRCGCG

>Sequence_111

ERIRQHFNQPVTITSGCRCA

>Sequence_112

QPVTITSGCRCATHNASVGG

>Sequence_113

CRCATHNASVGGARNSQHTL

>Sequence_114

VGRYNTFTHVDTRSNGPARW

>Sequence_115

EYTQGKTWKYYSNLPKDKNG

>Sequence_116

MMSKKFGAMILCSAAAVATA

>Sequence_117

MILCSAAAVATAFFTQQKNL

>Sequence_118

VYMIVNLEGCVRNPYKCPAD

>Sequence_119

GCVRNPYKCPADVWTNGVGN

>Sequence_120

NVGCGNIKTYYSKTQGKRVA

>Sequence_121

TYYSKTQGKRVATTLYRAAQ

>Sequence_122

MVTHPMRQGTYQVSSGYGPR

>Sequence_123

GTYQVSSGYGPRWGSFHAGL

>Sequence_124

YGPRWGSFHAGLDFAAPIGT

>Sequence_125

MNKLYKQLTAFISGFFGPQI

>Sequence_126

EQVIGKRGGVGYYPKSGFVH

>Sequence_127

FQIVEQNWNGRANKKPSKRW

>Sequence_128

RNERLWYHPTNFVEWHCANF

>Sequence_129

SCPHRSWEIHIGKGQPYTRR

>Sequence_130

KQEVLPTGWKKNKYGTYYKA

>Sequence_131

WKKNKYGTYYKAQKGSFING

>Sequence_132

YLPVGTWNGKKPPKNKMKEV

>Sequence_133

NGYRWITYINKSGKRRYIAT

>Sequence_134

GWQCFDLANMYWYKLFGHGL

>Sequence_135

WFIRPKYKTKTQSRSTQSPT

>Sequence_136

QSPTLEKKKITPKKKRKTQK

>Sequence_137

KITPKKKRKTQKIKYIQDYV

>Sequence_138

ATFQECARLLKKWGLPANRN

>Sequence_139

KKWGLPANRNTIRLHCEFVS

>Sequence_140

TKSTVAGAWKRNSYGSWYMS

>Sequence_141

FTNGSQPIMARTVGPFRSCP

>Sequence_142

YFIKQIRAYMNGKKPTSKVV

>Sequence_143

YMNGKKPTSKVVVSKPGSAS

>Sequence_144

RWGAAHRGVDFGRNGGSANM

>Sequence_145

VDFGRNGGSANMPVYAAQGG

>Sequence_146

GHPIFAVKSGTVARVGPASG

>Sequence_147

AVWPGEKPKATPVPAKPAVR

>Sequence_148

FTFGSPRPNSAITGICIHVT

>Sequence_149

LIKSTNGRLANHKACVKGLG

>Sequence_150

LANHKACVKGLGLRRINHTV

>Sequence_151

VIHATCPDVGYPSASKAGRA

>Sequence_152

VGYPSASKAGRAVSTAHYFA

>Sequence_153

AGRAVSTAHYFAEATRPASA

>Sequence_154

ILARDICHRHHIPVRKLTTA

>Sequence_155

KWSTKDALWSVWYYVLECRN

>Sequence_156

FDVANQYWLYLFGHTLKGVG

>Sequence_157

WFIRPFYAKEMTKNKVKSKA

>Sequence_158

KEMTKNKVKSKAKPVKKAKA

>Sequence_159

KSKAKPVKKAKAKKGKKILL

>Sequence_160

KAKAKKGKKILLVAGHGKGA

>Sequence_161

KALSSALGKTVGKIRGVTPR

>Sequence_162

INAPKSKPSKIKTTWNWGGK

>Sequence_163

SKIKTTWNWGGKFTANSTIK

>Sequence_164

VIKKDGYWWIRFKYVQPGSS

>Sequence_165

WIRFKYVQPGSSNKHFYCAV

>Sequence_166

PGSSNKHFYCAVCKITDKQQ

>Sequence_167

NIYGGKNPTANAINAFMSKV

>Sequence_168

TANAINAFMSKVKAAGYKPL

>Sequence_169

GSAVSQAPKKARVLKKKAHV

>Sequence_170

KKARVLKKKAHVYNKAGHRT

>Sequence_171

KAHVYNKAGHRTGKTIKKGT

>Sequence_172

GHRTGKTIKKGTSITTHGKK

>Sequence_173

KKGTSITTHGKKKTINGKKY

>Sequence_174

HGKKKTINGKKYYKIAKNQY

>Sequence_175

GKKYYKIAKNQYIRSCLLYT

>Sequence_176

RVWPAVERAAVLCRQLCDKH

>Sequence_177

PGPWFPWDRFMAVVNGGSSS

>Sequence_178

WSVWYYVLECRSRLARLEAD

>Sequence_179

PATGREPWTIGYGTTKGVRP

>Sequence_180

TIGYGTTKGVRPGMSITSAR

>Sequence_181

VHAGGKIMPGLVKRRAAERA

>Sequence_182

NQVAGDGWKYRGRGLKQITG

>Sequence_183

KYRGRGLKQITGLSNYRSCG

>Sequence_184

KTQLRFCHFFAQLAHESGNF

>Sequence_185

KWVKEIWGPTPAQSRYEGRK

>Sequence_186

THFIVPPVAKEVHTLTTKVK

>Sequence_187

TTVINKPGSASTPATRKNMN

>Sequence_188

YLKSLEGKAWNPDRTYGVQC

>Sequence_189

RPFYAKETTKNKLKSKAKPV

>Sequence_190

SKPSSKKTTWNWGGTFYPNA

>Sequence_191

TWNWGGTFYPNAPKSGIRVR

>Sequence_192

IKKDGYWWLRFKYQAPGSSK

>Sequence_193

MNPSIVKRCLVGAVLAIATT

>Sequence_194

NRQQWWLACNQLPRWIYVNG

>Sequence_195

GNAKDAINNNFKGLCTVYRN

>Sequence_196

RPHFTGQRQKQSKNSKKPSK

>Sequence_197

KPSKPSKPSKPSKPKTEFKW

>Sequence_198

SKPSKPKTEFKWYGRFTAHK

>Sequence_199

YKRDHYWWLGFHYVQKGSSK

>Sequence_200

DNGKQKTNCRGYDVIVGGAI

>Sequence_201

IRRCSNIWASLPGAGYGQNE

>Sequence_202

LTGHAVDVVAYVAGNVSWDW

>Sequence_203

MSAKIKTGIAGGICSVAIII

>Sequence_204

QRGLELIGNAEGCRRDPYNC

>Sequence_205

TFNKIFPHAVKGVYQAISAQ

>Sequence_206

FKENLNYSWLGLSKTFRKYF

>Sequence_207

WLGLSKTFRKYFPDPLTAKK

>Sequence_208

GDGKRYKGRGLVQVTGRKNY

>Sequence_209

RGLVQVTGRKNYTRWSARMK

>Sequence_210

VVGGWVARTPVARVLVAAGA

>Sequence_211

AAGALTAWDVFLGYVTGTVP

>Sequence_212

GGGGSDGSTAYAGPYRNGSK

>Sequence_213

TAYAGPYRNGSKDPGAPGPT

>Sequence_214

NGSKDPGAPGPTGGPVWRIQ

>Sequence_215

PGPTGGPVWRIQDRLKRAYA

>Sequence_216

RKPTASEVAAWAKSRIGKRL

>Sequence_217

SNNPPVGSVWVTNCLPYHQF

>Sequence_218

VWVTNCLPYHQFGHIGFVVA

>Sequence_219

DEANYFVNNLPSRPRYLVCD

>Sequence_220

RTWDAIVKNYGFIGRFIPRA

>Sequence_221

NYGFIGRFIPRAQDLAAVKL

>Sequence_222

GRIEEAIAAAAPIWASLPGA

>Sequence_223

DNHDWLWSASGCPPYAFYRY

>Sequence_224

ELANGDAGTPRPQPRPQLQS

>Sequence_225

NKSLLEKIIDFLLSIFKFKK

>Sequence_226

PWFPWDKFMAVVCGGSSDSG

>Sequence_227

GRKLGTSVNGTNWIGLFPKV

>Sequence_228

NGTNWIGLFPKVLNELMPEA

>Sequence_229

YPKAVWPSTISPAYAGGTVY

>Sequence_230

TISPAYAGGTVYHYIALMGY

>Sequence_231

GYKLDKRGSKPKGVVLHNDA

>Sequence_232

STIAGAWKRNNYGTYYMKEK

>Sequence_233

PPPPRSPPLYSSAASDVYKR

>Sequence_234

GKGVTSVGCVWWWCVVRYIP

>Sequence_235

CVWWWCVVRYIPAAHHSAGS

>Sequence_236

YPRTQVKQDTYYNCGPASCQ

>Sequence_237

APSTISPAYAGGWVYHYVAA

>Sequence_238

NMLPTIATTLATVLGRIFKG

>Sequence_239

LRGYKDPIGIVTACAGHTKT

>Sequence_240

GIVTACAGHTKTAVLGRPYT

>Sequence_241

VSFAYNVGVGAYCRSMTARR

>Sequence_242

TARRFNAGQWAGACKAMNQA

>Sequence_243

THKFTFGRPRPVANVVYIFI

>Sequence_244

PRPVANVVYIFIHVTVNTPG

>Sequence_245

LTGVPGTGLSTHKATRVWGG

>Sequence_246

WYWFTDSGSMATGWARINNT

>Sequence_247

MSNRSWPLPRNQVRISSRFA

>Sequence_248

PGEAPAKAPAPARAATAIAS

>Sequence_249

NKGRVAVVAAATGAVSTAGF

>Sequence_250

FTKPAEGAYTSGFGPRWGTM

>Sequence_251

MNRRITLSLAAVSAAAALAL

>Sequence_252

TVGGKNISDAGQALGAVSKA

>Sequence_253

DAGQALGAVSKAAGAIKESG

>Sequence_254

DGTVIAAGPATGFGNWVVIK

>Sequence_255

RFAFGRPRPTHQIRNIIVHV

>Sequence_256

GDKLNAGNAFLLWKMAGGKV

>Sequence_257

AFLLWKMAGGKVLNGLIKRR

>Sequence_258

GGKVLNGLIKRREKERQLFL

>Sequence_259

GVTYIFLLNILNYFFKNPFS

>Sequence_260

FRAKPYLCPAGVPTIGYGAT

>Sequence_261

LRWNRAGGKVLAGLTKRRTD

>Sequence_262

PVPKGFVVTSPYGPRWGTIH

>Sequence_263

TSPYGPRWGTIHYGVDYGVA

>Sequence_264

PMKQGTYTVSSGYGPRWGTF

>Sequence_265

LAGAAQPGGSVARPVGKQGG

>Sequence_266

DLGALAACWFWSKANINSSA

>Sequence_267

NIGRKTKPIGDAHGYANRKK

>Sequence_268

SSSKKTAPKQKVKKVSTKKE

>Sequence_269

AAKTLRCDVSAIRAVAEVES

>Sequence_270

MSFRLSARSRARMRGVHPAL

>Sequence_271

WDWPLYARIAEAFKAAAKAR

>Sequence_272

IAEAFKAAAKARGAPIVWGG

>Sequence_273

NTFGNGHGHVAWVIAATLNQ

>Sequence_274

FASNKASATIRKAKTKVTKP

>Sequence_275

TIRKAKTKVTKPKTKWNWKG

>Sequence_276

VTKPKTKWNWKGRFTTNTKI

>Sequence_277

NWKGRFTTNTKIKVRRSPGL

>Sequence_278

SIEKKDGYWWGKFKYPTNPS

>Sequence_279

WWGKFKYPTNPSAGYFYCAI

>Sequence_280

SSRWLYPLGNRKPDLLQFGS

>Sequence_281

DYPRDQVKQDTNYWCGPASA

>Sequence_282

QDTNYWCGPASAQTVIRSHT

>Sequence_283

APPNNYPRGVNGSVSPRYAG

>Sequence_284

GVNGSVSPRYAGGTVYHYIA

>Sequence_285

TPYGYWISHAQLASLIPPKG

>Sequence_286

VIAVGVCWHAGRGSWSGWPT

>Sequence_287

HAGRGSWSGWPTNNANFHTI

>Sequence_288

WPGVQIDAYKRGCAAILRRI

>Sequence_289

YKRGCAAILRRIGRGAGDCV

>Sequence_290

SGGTPMYAMAPGVVTAAGPA

>Sequence_291

MAPGVVTAAGPASGFGNWIV

>Sequence_292

HVTVAGGGKPTPNQRYGAAP

>Sequence_293

WRWPNFSAAEIACRGTGAIK

>Sequence_294

MTTTFYRHWRDVPAGVWRWP

>Sequence_295

PAGVWRWPNFSPAEIACRGT

>Sequence_296

ACPHRSAKLHTGINPIRQAW

>Sequence_297

LHTGINPIRQAWTKATQLKL

>Sequence_298

KQDTYYYCGPASCQTVILGA

>Sequence_299

PVLNKRIPGSHYCTVRMPND

>Sequence_300

YYSGGTVYHYIAVMGYAPGR

>Sequence_301

APGRFWIADSGFYPYGYWIS

>Sequence_302

YYCAPASCQTVVLGATGHIV

>Sequence_303

AGFQCFDYANVGWDKLFGHG

>Sequence_304

NGGWTAGPINNGTGWETATR

>Sequence_305

SSKSKKTTPKITWNWKGRFT

>Sequence_306

PKITWNWKGRFTSNSTVKVR

>Sequence_307

KKDGYWWVKFKYPTNPKAGY

>Sequence_308

KFKYPTNPKAGYFYCAVCKI

>Sequence_309

KAGYFYCAVCKITDIQARIK

>Sequence_310

GRADLGNTCPGDGVRYAGRG

>Sequence_311

CPGDGVRYAGRGPIQLTGRA

>Sequence_312

AGRGPIQLTGRANYKAFTRW

>Sequence_313

PKWGFLAAAYYWTAARPQIN

>Sequence_314

QDTYYYCGPASTQTIIRAAT

>Sequence_315

PARYNTRNFDGIVGWFYFPT

>Sequence_316

WCDVDGYIWISYIGGSGNRR

>Sequence_317

MTVNKTKAQAHAYLGKLKGY

>Sequence_318

MFFIRPLYKAKATVVNKVKD

>Sequence_319

KAKATVVNKVKDKVTSVAKP

>Sequence_320

KVTSVAKPTSQGKKILIASG

>Sequence_321

AINGKPIGGTSAGSKKITWN

>Sequence_322

GTSAGSKKITWNWKGRFTPN

>Sequence_323

FQRLAGNYKQFNPHLQHIGY

>Sequence_324

AHVKGHNLSSLGICLVGGIT

>Sequence_325

PIWASLPGAGYGQREHDLGA

>Sequence_326

YFIRFNFPKNISVVKKAKRK

>Sequence_327

KNISVVKKAKRKLSSNKASG

>Sequence_328

NAFGCQCFDTANQYWLYLFN

>Sequence_329

PMWFIRPFYAKETTANKIRS

>Sequence_330

DFNRKNIIPRVKKHLESVGN

>Sequence_331

PSSKPSADKITWNWKGVFYP

>Sequence_332

KITWNWKGVFYPNPEKAIRV

>Sequence_333

RWDNYYGCTHFIRPKYKSEG

>Sequence_334

THFIRPKYKSEGLMNKITNK

>Sequence_335

KSEGLMNKITNKVKPPAQKA

>Sequence_336

ITNKVKPPAQKAVGKSASKI

>Sequence_337

NRTTVNLHRQYFGTSCPHRS

>Sequence_338

RQYFGTSCPHRSWDMHVGKN

>Sequence_339

DYFISRIKHYYNGGTKTTWK

>Sequence_340

HYYNGGTKTTWKWSGKATAK

>Sequence_341

TTWKWSGKATAKKGVSPIAA

>Sequence_342

GYWWAEFEYPTNPKAGRFYC

>Sequence_343

YPTNPKAGRFYCALGPITHK

>Sequence_344

PVAKEVKKVKTASKEAPKQK

>Sequence_345

PHRSLLLHTGLDPLYHSITK

>Sequence_346

QGTYTTSSGFGPRWGTNHNG

>Sequence_347

QGRDRAPGSVGGFGNWIWQD

>Sequence_348

TPPGRTGGRPVDPAPRLRNA

>Sequence_349

DAPAPPQPPAPAKTLPAGAK

>Sequence_350

TIISGARWAAATCYHEKLGV

>Sequence_351

FTATGCPVHLQGPKAGNAWG

>Sequence_352

HLQGPKAGNAWGGKAGKYHN

>Sequence_353

GDGWKFRGRGLIQITGRANY

>Sequence_354

GTNGLDDRKKRLERAKKALG

>Sequence_355

MVALTTGAVTTGGAAVASAA

>Sequence_356

EGAYTSGFGPRWGTFHKGID

>Sequence_357

GQTVRAGQRIAGMGSRGWST

>Sequence_358

IAGALALALPLAALPANAFT

>Sequence_359

VMPTEGRFTSGFGPRWGSVH

>Sequence_360

GFGPRWGSVHRGIDIANNIG

>Sequence_361

KHHRKSSKASKSVAGNRNKK

>Sequence_362

FINSGYRCPLLNQLLHGVGN

>Sequence_363

FEHSKFGVIWLHVSCKPNKA

>Sequence_364

IWLHVSCKPNKAQNRHMALR

>Sequence_365

YSRWTDPKPGTKYGRDYATR

>Sequence_366

TGNHLHFGLKVNGKYVNPYP

>Sequence_367

NPYPVIANGTIPTTYAAAIG

>Sequence_368

GTIPTTYAAAIGSEACAGAY

>Sequence_369

AGAYGGLSPSNGKAMSKKFP

>Sequence_370

TVPDKRISGAKITPRLAKLR

>Sequence_371

SYGSNSSSTVGWKKINGSWY

>Sequence_372

TVGWKKINGSWYYFKSDGSK

>Sequence_373

SGAMKTGWYQVSGKWYYSYS

>Sequence_374

NYALGFGNRLLTGNSKAEPV

>Sequence_375

QWLDPRVWPAVEKAAVLCRQ

>Sequence_376

MKSIVKRCSVAAVLALAALM

>Sequence_377

TPYQCSAGVWTSGIGHTAGV

>Sequence_378

HIYDALVSFSFNVGTGAACR

>Sequence_379

FSFNVGTGAACRSTLVSYIK

>Sequence_380

AACRSTLVSYIKRHQWWQAC

>Sequence_381

INKGLENRRARERAYCLKGV

>Sequence_382

GVGVWTIGYGTTVINGVKVK

>Sequence_383

WHQKRGFKRNPTYIKRFNPH

>Sequence_384

RNPTYIKRFNPHLKHVGYHF

>Sequence_385

FNPHLKHVGYHFIIDTNGTV

>Sequence_386

VKGHNQNSIGICLVGGITGM

>Sequence_387

IGICLVGGITGMGKNHGEYT

>Sequence_388

QALHKLLRKLESRFPSSRIC

>Sequence_389

TSFVSLRKYAIVTRLRIAAF

>Sequence_390

PWGIARHPSLGLCSQIHLSR

>Sequence_391

GVATLCGVGIAYHAGRGSKP

>Sequence_392

GIAYHAGRGSKPGWPTNNAN

>Sequence_393

MKSTTKKIKTTLAGVAALFA

>Sequence_394

KTTLAGVAALFAVFAPSFVS

>Sequence_395

MTISTEVGTGTGDNRKWWCP

>Sequence_396

TGTGDNRKWWCPANDRVPAT

>Sequence_397

WWCPANDRVPATKNFPNDSK

>Sequence_398

LGRNSTGLRRTFRAGLAALI

>Sequence_399

SAAEQFGRWVKAGGKTLAGL

>Sequence_400

WVKAGGKTLAGLVRRRAAER

>Sequence_401

APATPWTPPAVPGTIFGIDI

>Sequence_402

EAIHKCRSRWASLPGAGYGQ

>Sequence_403

MKKLGLILAAILCLVVLIVV

>Sequence_404

MMILTKTRKALGVCSVITVM

>Sequence_405

IGNAEGCRRDPYQCPADVLT

>Sequence_406

ACGEFPRWVYASGKKLAGLV

>Sequence_407

GPIQLTGRANYRAFTDWCRS

>Sequence_408

AAVATSRWGFLAASWYWTVA

>Sequence_409

GFLAASWYWTVARPKINGLC

>Sequence_410

NGGTHGLADRRARFDRCRRL

>Sequence_411

QVRWDWPLYAQISAAFKQAA

>Sequence_412

KAPYNLKWSKGACLLYTSPS

>Sequence_413

RKFLFGRARPVKDIKIICIH

>Sequence_414

GFPWDVYLRYVKEYMGNPNK

>Sequence_415

NPNKNTPSPAPAPAHKRVWP

>Sequence_416

PAPAPAHKRVWPMAKDSFRI

>Sequence_417

RVWPMAKDSFRISSGFGPRG

>Sequence_418

FIKGFFGPQIDALQDVWTQL

>Sequence_419

QIDALQDVWTQLRGPGGRGW

>Sequence_420

WTQLRGPGGRGWKQLGKNDK

>Sequence_421

FIYPGQKLKVPGKGGSGSSS

>Sequence_422

SSNGGYYTPVFCHQNLYTWG

>Sequence_423

PVFCHQNLYTWGQCTWHVFN

>Sequence_424

YTWGQCTWHVFNRRSEIGKG

>Sequence_425

TIDYRPTVGSIAQTDAGYYG

>Sequence_426

FRSCPVNGVLQPGQTVRYDT

>Sequence_427

PKSKPSKSKNTWNWSGTFYP

>Sequence_428

KNTWNWSGTFYPNAPTKGIR

>Sequence_429

PNFKAAEKMTWNWAGRFTAN

>Sequence_430

MTWNWAGRFTANTTIKVRRS

>Sequence_431

DFVSITKKDGYCWIKFKYPT

>Sequence_432

YCWIKFKYPTNPSAGYFYLV

>Sequence_433

PTNPSAGYFYLVVCKITDKN

>Sequence_434

WKGVPVRYQLLTIGTRRPGK

>Sequence_435

YWWIRFKYVQPGSSKDDFYC

>Sequence_436

AYMSTRLPHHFYRIVGSGSA

>Sequence_437

ALLPDAALAAGWFWTPYKNI

>Sequence_438

DVITDFVHHVTKGVRFWGNA

>Sequence_439

HVTKGVRFWGNAKDLINNVM

>Sequence_440

WGNAKDLINNVMPKGWKVVE

>Sequence_441

AVDLVPYINGKLRWEWPAIY

>Sequence_442

NGKLRWEWPAIYPIAAAVWQ

>Sequence_443

GRWMNEIWGPTPAQRRYEGR

>Sequence_444

DAGLAAGWFWGPYKRLNKIA

>Sequence_445

GKYAAHTLNCNTGSIGVAVA

>Sequence_446

AGFDPGPADGIYGTRTKAAA

>Sequence_447

RTVGLVADGIIGAASWPKLM

>Sequence_448

MKPIRLLVVHCSATPANRDI

>Sequence_449

EQLLGMLHTRYPLARICGHR

>Sequence_450

DRNGDGKVTPGEWVKACPTF

>Sequence_451

TPGEWVKACPTFDVAAWWAQ

>Sequence_452

MYLPLKRGTYRISSGYRTKS

>Sequence_453

RTKSRPKHRGIDFSAPVGTP

>Sequence_454

RGIDFSAPVGTPIYAPFDGT

>Sequence_455

VIQGKDRAPGSVGGFSNWVW

>Sequence_456

TGPHAHCELWSRPGRIGGHD

>Sequence_457

LWSRPGRIGGHDIDPTRFWG

>Sequence_458

RFWGDNAQNPGGAPAPARKA

>Sequence_459

NPGGAPAPARKAPAVAASNV

>Sequence_460

WTRKFVFGRHRPTNQIKIIC

>Sequence_461

RHRPTNQIKIICIHVTVNKP

>Sequence_462

GKGFPWDVYLGYVRGYLNGT

>Sequence_463

YLGYVRGYLNGTITNTPQKQ

>Sequence_464

MLTTQYFKDFITGFIGPVIS

>Sequence_465

KWARQTPNRARRVTEVIRTG

>Sequence_466

ESTIGWHAPPNPHSLGCLLY

>Sequence_467

MTTMPVPRGFKVTSPFGPRW

>Sequence_468

SNKYSPWGIARHPQLGLCSQ

>Sequence_469

CGAGIAYHAGRGWKSGWPTN

>Sequence_470

YHAGRGWKSGWPTNNANWTS

>Sequence_471

AQFNFGNPRPTNNIRIICLH

>Sequence_472

HFIVPPVAKEVKKAPKKVKE

>Sequence_473

LKGAKWVGNKPAPVRAATAV

>Sequence_474

LDWSQRFTFGRPRPTHQIRN

>Sequence_475

KWGLPANRNTIMLHCEYFAT

>Sequence_476

VVFPGKYGSGAGHVAIVTKA

>Sequence_477

IRLDFPTKISAGTKAKQIIK

>Sequence_478

TAYGVRVGNKKEYGMYWVNK

>Sequence_479

YRDGGGIWTICRGATMVDGK

>Sequence_480

IGPGKCFPSTFYKRINAGDR

>Sequence_481

DGGKDCRIRSNNCYGQVSRR

>Sequence_482

LDKRGAMVTGWMFLNHRWYF

>Sequence_483

TGWMFLNHRWYFFKSDGRMA

>Sequence_484

RWYFFKSDGRMATGWVKYRE

>Sequence_485

FNDWTVCPGTFVYNQLDAIA

>Sequence_486

ATKTATKKKTTAKKKMKKLT

>Sequence_487

EVKGYRLPNRGYKPKGIVLH

>Sequence_488

ARLLNKWGLKANRNTVLLHM

>Sequence_489

IKQIRAYQAGKVPTASVAKG

>Sequence_490

GNAPIMVRTTGPFRSCPYAY

>Sequence_491

SWGWQCFDEANMYWFKLFGH

>Sequence_492

TSFTREEFVKCLKSTVGKQY

>Sequence_493

VKCLKSTVGKQYDYDLYAAF

>Sequence_494

QMWFIRPKFSNKKTESKLLK

>Sequence_495

PTNPSSGYFYCALCKITDKQ

>Sequence_496

RDLGAWAGLAWTLAEAWCDT

>Sequence_497

EPPVVLPPANPPILKPPPLP

>Sequence_498

YTGAKFALVKLTQGTGYLNP

>Sequence_499

VKLTQGTGYLNPKAKGPVSY

>Sequence_500

YLNPKAKGPVSYTHLTLPTN

>Sequence_501

GVTDVGRVWGWCVVRYIPAA

>Sequence_502

LEGKYIDFDGWYGLIGSPCK

>Sequence_503

ENNWRGGGWTNGPAQGGTGW

>Sequence_504

WTNGPAQGGTGWEKATRRRH

>Sequence_505

SYSTKTTTKKPAAKKKKAKK

>Sequence_506

KPVASGWKRNNYGTYYKSER

>Sequence_507

NGNTPIITRTVGPFRSCAQA

>Sequence_508

RTVGPFRSCAQAGLLPAGAT

>Sequence_509

VWVSYVTNKGYRVWLPIRTW

>Sequence_510

KGYRVWLPIRTWNGVAPGQP

>Sequence_511

IRTWNGVAPGQPGYSVGSLW

>Sequence_512

FDGWYGLIGSPCKILWIAGN

>Sequence_513

YWRNPEGGASSYIRRAAMRL

>Sequence_514

DKVYGYQCFDSVNYYWYKLF

>Sequence_515

FDSVNYYWYKLFGHGLKGAG

>Sequence_516

TDTNKKTKAKKKMKKLTYIR

>Sequence_517

AARLLNKWGLKANRNSCRVH

>Sequence_518

KQRYYLPIRTWNGIAPPNQG

>Sequence_519

MLTVKYFTDYIKGFIGPLIS

>Sequence_520

THFIVPPVAKEVKKLSVVKK

>Sequence_521

VVKKEAPKQTKPTVKKSLPV

>Sequence_522

FTPNTAIYTRFVGPFTTCPQ

>Sequence_523

NTVVFPTSGTFTSGFGPRWG

>Sequence_524

PRWGSFHNGIDVANPIGTPI

>Sequence_525

TPVKSSPSKSTSKPKTTSKK

>Sequence_526

TSKKTYNLPSGILKVTKPLT

>Sequence_527

PSGILKVTKPLTKGSGVKAL

>Sequence_528

DGYYGPKTANAVKRFQLMHG

>Sequence_529

MLKVARKIVTPVVLRGAMVV

>Sequence_530

KHGVRVIGCYSYVPYWEGHV

>Sequence_531

YKPYRDVVGIQTVCYGHTGK

>Sequence_532

DGYRYLGRGPIQITGRSNYA

>Sequence_533

AGRDCKLPQNWGPKGCRGVW

>Sequence_534

QNWGPKGCRGVWTRQQERHA

>Sequence_535

MANRCWPLPKNKIRITSRFA

>Sequence_536

PKNKIRITSRFAGRINPVTG

>Sequence_537

GDGRRFKGHGLIQVTGRGNH

>Sequence_538

HGLIQVTGRGNHGRYSRWKY

>Sequence_539

RPFSAGKYPITPAQVDALAT

>Sequence_540

HPVSANAGPLVAGRYAAHTL

>Sequence_541

GSAGPGAAPAVPPFTSTPGI

>Sequence_542

EVSSGFGGRGNPTGGGWQNH

>Sequence_543

MAQRFRPLPSGTKVTSPYGP

>Sequence_544

PSGTKVTSPYGPRDGGFHGG

>Sequence_545

PVHACQAGTVIFAGAASGYG

>Sequence_546

GCPHPNTVPAPAPAPVSGGT

>Sequence_547

NEDLQLTRTAALVAWRCAVR

>Sequence_548

RYVGGRGMPKSPGVCGHVDF

>Sequence_549

FDRGHGDFGAIWGPFMHHTG

>Sequence_550

GAIWGPFMHHTGSFGETPRG

>Sequence_551

IHLASNGVATLCGVGIAWHA

>Sequence_552

ATLCGVGIAWHAGTGSWPGI

>Sequence_553

AWHAGTGSWPGIPTNNGNQV

>Sequence_554

SYLKIVRAINKRLGNPLNKV

>Sequence_555

INKRLGNPLNKVVAHKEYGA

>Sequence_556

DDPKVGAHPVGGVIGQRWAL

>Sequence_557

FGSLEWDPSGAVKKIGPAAT

>Sequence_558

SGAVKKIGPAATNLSLVNAQ

>Sequence_559

YNCGNVFVADGWAGFSHAYC

>Sequence_560

ADGWAGFSHAYCASMIPKYR

>Sequence_561

QRINLKSGGVPANRNTVRLH

>Sequence_562

PRGLLKHVAPTLRGGVIKII

>Sequence_563

GRTKPGKKVTNAKGGQSNHN

>Sequence_564

LYLKIGKWAKSAGLLWGGNW

>Sequence_565

HGPGGGMNSHMWCDIDGMRV

>Sequence_566

FLAASWYWLHGGPRKGQINA

>Sequence_567

CVNGWIDGVMPVGWADRRTR

>Sequence_568

VMPVGWADRRTRYLNCMAMG

>Sequence_569

MAYVDGVWWWCAVRYIPAAH

>Sequence_570

GAQCFDLANKYWNKLFGGQL

>Sequence_571

KRELEARGYHVPGVYTGRWY

>Sequence_572

DGGANHRGWSYPLGDRKPDI

>Sequence_573

SLEKNGIKFAFVWQFRGGSG

>Sequence_574

LVAGLGGGRVLGWVTKSWSQ

>Sequence_575

RVLGWVTKSWSQGVTGSDYA

>Sequence_576

WKPIDPSPKKVTKKVNIPTQ

>Sequence_577

KKVTKKVNIPTQYQKIRPNP

>Sequence_578

YTATLCGVGVAWHLGKGSYP

>Sequence_579

GVAWHLGKGSYPGLPTNNAN

>Sequence_580

IGRGTLSALFRKLGASNARA

>Sequence_581

NGVPVRFDLLPFGTRRYGQK

>Sequence_582

TPQAKHTKPKTTWNWSGVFY

>Sequence_583

PKTTWNWSGVFYPNTTIKVR

>Sequence_584

KLPEGWRLVLNRPSTVPKKG

>Sequence_585

KPSKRWDNYYGLTHFIAPPV

>Sequence_586

GDMLTKKYFTDFIKGFIGPI

>Sequence_587

GAIDWRNPRSYISKYFTVAE

>Sequence_588

RPEPINSQVGGVHGSKHCLG

>Sequence_589

GIKFAFVWQFRGGGSNPSNA

>Sequence_590

GVVGHLMDMNKFRSHVQYYI

>Sequence_591

MNKFRSHVQYYIQNPPFKKE

>Sequence_592

YKSRYPGSNFKGSLRDYILN

>Sequence_593

SKPSKAKTTWNWAGKFTANS

>Sequence_594

QPGSSDKHFYCAVCKITCLL

>Sequence_595

AGSKYGRWYAGYKGAYFGAS

>Sequence_596

RGDIFIWGRRGYSSGAGGHT

>Sequence_597

NWYWFDSSGYMATSWKKISG

>Sequence_598

GYMATSWKKISGKWYYFNRD

>Sequence_599

GNIPARRGDVFIWGIRGASG

>Sequence_600

INGCPPVTIYRPPAEGPSAD

>Sequence_601

QFTTITRRINGGLNGLEGRL

>Sequence_602

QVIWAVMVLLGLGIKLARHG

>Sequence_603

LLGLGIKLARHGQARTGRHS

>Sequence_604

GRHSFWWQLFGSATVALLLW

>Sequence_605

LFGSATVALLLWSGGFFSQA

>Sequence_606

LLLWSGGFFSQARAAQPPQA

>Sequence_607

LQILPHARQFAGVFVPVINT

>Sequence_608

LEQPQYACLSAAWYWATNGL

>Sequence_609

LSAAWYWATNGLNTLADAGH

>Sequence_610

LQILPNAGPVAGVFVPVLNA

>Sequence_611

PVAGVFVPVLNAAMGHYQII

>Sequence_612

HACMSAAWFWATKGLSTLAD

>Sequence_613

AYLDPVGIPTICEGVTKGVK

>Sequence_614

VRVPLPDTRRAALGSFVYNV

>Sequence_615

LLNAGDARGACAQLSRWIYA

>Sequence_616

GACAQLSRWIYAGGKQLAGL

>Sequence_617

GRPPGAAAIKAAGHVGAVRY

>Sequence_618

LYQRIIDTVSTPGPRVGGTI

>Sequence_619

QHPRTPATPAPVPPREVTPM

>Sequence_620

PAPVPPREVTPMIRPTPGHR

>Sequence_621

TGWRGRGQGDFGAIWGTIAH

>Sequence_622

GDFGAIWGTIAHHTGDNNTP

>Sequence_623

TGAGVAYHAGMGSWPGIATN

>Sequence_624

AGMGSWPGIATNGANQVTIG

>Sequence_625

RICAAISWYLGHSSLRVIGH

>Sequence_626

SGEEFGFLGAVWYWTVARPK

>Sequence_627

DYNHGYLPQDTGYYCGPAST

>Sequence_628

QDTGYYCGPASTQTVVWTAT

>Sequence_629

MSLSLPITKIVIHCSATKNG

>Sequence_630

RGFKRSPVLAKQFNPHLQHL

>Sequence_631

QEKLYAIGRITLGKKVTNAK

>Sequence_632

IFAKYGWEWGGNWKTFKDLP

>Sequence_633

PSFLPKPGDVVVWNRKYGGG

>Sequence_634

GYRLPNRGYKPTSITLHNDA

>Sequence_635

RGPIQLTGRNNYGAFGRWCH

>Sequence_636

RNNYGAFGRWCHDRGIINDP

>Sequence_637

PKWGFMAAAWYWVIARPRLN

>Sequence_638

GWQRYEGFKAATRAINGGTN

>Sequence_639

YYNCGPASTQTVVFAATGIL

>Sequence_640

TVNGTDWIGFFPKVLNKHIP

>Sequence_641

GFFPKVLNKHIPGAEYQSVE

>Sequence_642

RLWNDIKTSVKAGHGVICNI

>Sequence_643

ICNIVAPPSNYPRGVYGSIS

>Sequence_644

GSISPAYGGGVVYHYISAMG

>Sequence_645

DGPRYKGRGPIQVTGRNNYA

>Sequence_646

TLGTTTPSYGGGTVYHYVCI

>Sequence_647

YGGGTVYHYVCIVGYEVNGG

>Sequence_648

GTAVITGVGIAWHAGTGWFQ

>Sequence_649

GIAWHAGTGWFQNWPTNAAN

>Sequence_650

ALALPKHKAVCQRLCKAVAD

>Sequence_651

AVCQRLCKAVADVTGWKLRG

>Sequence_652

ALWKQRKWPICRAIAAAIAA

>Sequence_653

NNEWFYFDERGYCLINRWFN

>Sequence_654

RFNFPKNVSVVKKAKQKLSS

>Sequence_655

IAGAIHGSPIGGVVASKKKS

>Sequence_656

PIGGVVASKKKSSSKKLNVP

>Sequence_657

KKKSSSKKLNVPKTIPSGYK

>Sequence_658

SGYKINNKGVPYKKEKGRYT

>Sequence_659

NDGAYGFQCADVPCYGLRHW

>Sequence_660

CADVPCYGLRHWYGVTLWGN

>Sequence_661

FTDSGSMATGWTRINNAWYY

>Sequence_662

LPNAGQVAGVFVPVLNTAMN

>Sequence_663

NVYAGRMGNTAPGDGWKYRG

>Sequence_664

NTAPGDGWKYRGRGLIQLTG

>Sequence_665

GWKYRGRGLIQLTGKTNYRL

>Sequence_666

MNSTAKKCAVLTIVALAALL

>Sequence_667

CSAGVWTNGIGHTRGVTPLS

>Sequence_668

ATVSFAFNVGVGAACGSTFA

>Sequence_669

QQKWRAACSQLPRWIYVDGK

>Sequence_670

DLPCYIMRQFFDVSLYGNAY

>Sequence_671

VGGPARYHERTVGEIVGYIV

>Sequence_672

NGYRWISFVGASGSRNYMAI

>Sequence_673

LAITLPALVNPKAGGYIGGT

>Sequence_674

VNPKAGGYIGGTAEHQRLAM

>Sequence_675

RWYAQVTGSPSFGASGVPYC

>Sequence_676

LAKAAQPTTPPKKALLFMCK

>Sequence_677

HCCCLLQRKMTTTCPLSFRM

>Sequence_678

VFQFIANKLNKWGLPANRNT

>Sequence_679

IFIWGRRGYSAGAFGHTGMF

>Sequence_680

PAPVKKGWQEDSKGFWWARG

>Sequence_681

QEDSKGFWWARGNGTYPASR

>Sequence_682

WKKINGYWYYFNRDGAMQTG

>Sequence_683

WMRVGQPYYYCYRYKGAPAT

>Sequence_684

IKFALIKLTQGTGYINPKAK

>Sequence_685

YKPLVYAGAYVLRNRLNTAR

>Sequence_686

AYVLRNRLNTARIIKSFGTC

>Sequence_687

NTARIIKSFGTCLWVASYKV

>Sequence_688

GTPLRLPFQRGFPHIILQAF

>Sequence_689

RKIRADGYSVWRRGGTARGG

>Sequence_690

SVWRRGGTARGGGETPPVAL

>Sequence_691

AYEGRCSDLGNCFPGDGVRF

>Sequence_692

DEFAFVGFAWYWITRRNGNG

>Sequence_693

IDQAVGVLNFIAATNPAILG

>Sequence_694

MPFGGGSLRLNWGLKMSKFK

>Sequence_695

TMNKLKNYFIKQIKAFMNGK

>Sequence_696

TCTVRQGIITRRTGPFVSLP

>Sequence_697

SLTMFLFFASWGIWWSFFQI

>Sequence_698

ASWGIWWSFFQIWLTSPSVG

>Sequence_699

FFIMLCGILVVAVKKFQKAN

>Sequence_700

DWRLALAAYNAGPGNVNKWL

>Sequence_701

RAASTARYPLSAYLGRRTPG

>Sequence_702

KAICQKLCSAISDVMSIPVR

>Sequence_703

REYFSTSCPHRSWAIHVGVG

>Sequence_704

SDNQYVKYHWRGKFTAHKTN

>Sequence_705

HWRGKFTAHKTNTLPIVPRY

>Sequence_706

DKQAKLWWIKFKYVKKGSSD

>Sequence_707

FDNGYVDHPRKKITRKMGGS

>Sequence_708

DGIYGPATKAKVAAWQAAHK

>Sequence_709

FRGNAYPDPATGGKPYTIGN

>Sequence_710

DVKKFEAAVSKAVKVKLTQG

>Sequence_711

SFTYNVGPAKMTSSTLIKKL

>Sequence_712

LRWNKAAGKVMAGLTTRRAA

>Sequence_713

FSTKPKKKQPKITWNWKGRF

>Sequence_714

NPSAGYFYCAVCKMTDIQGR

>Sequence_715

VAKYYKKDVKVTPVKTPKTA

>Sequence_716

PKTATKTEQKANKKVMKGYA

>Sequence_717

KGYATQTQVNARLKGYLNKK

>Sequence_718

LPPKRRVDNYFGCAGFWVPN

>Sequence_719

NYFGCAGFWVPNVGKTVTQK

>Sequence_720

WVPNVGKTVTQKIATVTKKA

>Sequence_721

VTQKIATVTKKAASKTTPKK

>Sequence_722

TKKAASKTTPKKTKWAWKGR

>Sequence_723

TPKKTKWAWKGRFYPNAKKG

>Sequence_724

WKGRFYPNAKKGIRVRRSPT

>Sequence_725

FDQIIKANVYWWIRFKYPTN

>Sequence_726

GYFYCAVCKITDKKERIKHE

>Sequence_727

YSRWADKGSGTKYGRWYAQA

>Sequence_728

TNKWQKINNAWYYFDSNGYM

>Sequence_729

NGYMKVNSWHKHSDGYWYYL

>Sequence_730

WHKHSDGYWYYLLPNGSMAT

>Sequence_731

WYYLLPNGSMATGWVLISNK

>Sequence_732

ISNKWYYFKEDGKMATGWVK

>Sequence_733

VEWLRYRDKALGIAHYYCNR

>Sequence_734

IIPKVKKYLEKSGHNVVLYG

>Sequence_735

PMWIDVETNVGLHVGHIRAC

>Sequence_736

YPGDQHRQWDYPLGNQKPAL

>Sequence_737

IGKAVDIVPLVNGKISWDFN

>Sequence_738

PLVNGKISWDFNHYYPLAKA

>Sequence_739

RWGGAWTVITGKSGTPQEWV

>Sequence_740

IYRRLYWTRPAFDQIAPRAP

>Sequence_741

MGPAVAATFLQRALNALNRG

>Sequence_742

NLNYSWSGLSQTFRKYFPDP

>Sequence_743

DLWYAFQCFDYANAGWKALF

>Sequence_744

FDYANAGWKALFGLLLKGVG

>Sequence_745

VGWHTANQIGNKYYYGIEVC

>Sequence_746

SASSNTVKPVASAWKRNKYG

>Sequence_747

GNQPITVRKVGPFLSCPVGY

>Sequence_748

KVGPFLSCPVGYQFQPGGYC

>Sequence_749

TGEVDKAGNRISGFGKFSAV

>Sequence_750

SQYPNRSWKIYRNTPSFVAK

>Sequence_751

YAAVPAQLRRWTKGRVKGVL

>Sequence_752

RCVESLTYSAQRLAAVWPRR

>Sequence_753

DGWRFRGRGLLQVTGRSNYR

>Sequence_754

RPQLLAEPRWACRSAAWWWQ

>Sequence_755

RWACRSAAWWWQRNGLGELA

>Sequence_756

TDYSDHPFAHGRPAKVFNRR

>Sequence_757

TASGRYQHLYLFWPHYRKQL

>Sequence_758

AGRIERAISRCRNIWASLPG

>Sequence_759

MSKTKFWVIGLAASAAFFTS

>Sequence_760

WLDPRVWPCLLYTSPSPRDG

>Sequence_761

DGIVVLPANATFTSGFGTRW

>Sequence_762

GPASGFGNWIRISHPDGSMS

>Sequence_763

QMCIRDRYKLDKRGSKPKGV

>Sequence_764

QLKLIDYFIERVRHYAGGGK

>Sequence_765

PALHYGATVHGYPRILGHGA

>Sequence_766

KFIKNESWHCVTQCAVNVRS

>Sequence_767

YRWISYISWSGKRRYVAYRR

>Sequence_768

WSGKRRYVAYRRLSGNTRPW

>Sequence_769

FPMWFIRPKFKTATATRSTQ

>Sequence_770

STKKAAPKKKAKPVKLNIIK

>Sequence_771

KKAKPVKLNIIKDVVKGYNL

>Sequence_772

NIIKDVVKGYNLPKRGYKPK

>Sequence_773

ARPAEGTFTSGFGTRWGTFH

>Sequence_774

TLGVSVGERVKAGQYIAGMG

>Sequence_775

INIIWGGAWGRYLNHYVTAN

>Sequence_776

LDTGVNCGIKFAKPLLQRAL

>Sequence_777

IKFAKPLLQRALNLLNNQGK

>Sequence_778

PVGVWTIGYGHTTAAGPPKV

>Sequence_779

DEFLKWTKAGGKTLPGLVKR

>Sequence_780

TASSSPPTLTLIALLVFVAL

>Sequence_781

LTLIALLVFVALLAVAIITK

>Sequence_782

MKKWIATLTLGITVATSLSP

>Sequence_783

MNRRLLSTALAAVLVLTAAA

>Sequence_784

VTPTEGTFTSGFGSRWGTLH

>Sequence_785

LLSTAVAAVLALTAAAAPQA

>Sequence_786

TEGRFTSSFGPRWGRLHNGI

>Sequence_787

GDLGNNYAGDGRRFKGCLLY

>Sequence_788

QMWFIRPNFSRKPQAKKTSV

>Sequence_789

SSKKKPKITWKWKGRFTSNT

>Sequence_790

PNGKFMFSSRSTSLLSKVHK

>Sequence_791

SRSTSLLSKVHKDLQKVINR

>Sequence_792

VRNWEGGYFHKMAKAVKQAA

>Sequence_793

MAATHPMKPGTYTVSSPFGP

>Sequence_794

PGTYTVSSPFGPRWGTHHNG

>Sequence_795

HFEEWIRPGRLGGRPVDPAP

>Sequence_796

GRLGGRPVDPAPRLRGAASP

>Sequence_797

DPAPRLRGAASPGGAAPAPA

>Sequence_798

AASPGGAAPAPAPAAPTAPV

>Sequence_799

PAPAPAAPTAPVRAATAVAA

>Sequence_800

VLIVACPCALGLATPMSLMV

>Sequence_801

VNLRKGPDTGYGVIRQLGKG

>Sequence_802

SFVYNLGCGSLTHSTFGNQI

>Sequence_803

MSVQQSIINWFVARRGLITY

>Sequence_804

NWFVARRGLITYSMYGSRNG

>Sequence_805

DTNLKPLKCYWWTSAAPVYV

>Sequence_806

CYWWTSAAPVYVRVFGVLNP

>Sequence_807

GDPTHFIPKIAVPIRADCTK

>Sequence_808

DTGRGGYNGGWYWRLFTFGR

>Sequence_809

RGLIQITGRRNYLACSQALF

>Sequence_810

VTSKFGPRWGTIHWGTDYGC

>Sequence_811

AGAARGFGRWVTIDHPASNG

>Sequence_812

LTKKFFTDFIKGFIGPVISD

>Sequence_813

FTDFIKGFIGPVISDVKDIR

>Sequence_814

LRGPSGNGWPQLGKDRQGRN

>Sequence_815

GLNNFFIYPGQKLKVSGTAS

>Sequence_816

YDPSKGAEQWRPLLKWAINF

>Sequence_817

QWRPLLKWAINFQHNGIADT

>Sequence_818

CSPPREPWMKAKQPPREYLD

>Sequence_819

VEYFRGAASVLGRQRVGIYG

>Sequence_820

VAPGRVLGWVTSSWNAVGYE

>Sequence_821

EWGWRAIPALPAGSKALPDV

>Sequence_822

NIVDWTHKFTFGRPRPVANV

>Sequence_823

SGKTTGLSAPNGFRRAVYLP

>Sequence_824

WTESRYNPFAISRVGAAGLG

>Sequence_825

FAISRVGAAGLGQLMPGTAK

>Sequence_826

LAYNIGVAGYCGSTAARRIN

>Sequence_827

GYCGSTAARRINAGRIREGC

>Sequence_828

GVLRPVKGLTARRQRERALC

>Sequence_829

STALIGVGVLMNPAATAACL

>Sequence_830

VLMNPAATAACLPGSLIVGQ

>Sequence_831

QLTHAATIITVGARTAGVGR

>Sequence_832

YPDPATGGAPWTICWGHTGP

>Sequence_833

APWTICWGHTGPEVVKGLTV

>Sequence_834

YSKWIYADKRIFQGLVTRRY

>Sequence_835

FVPKWYRDPVGVWTIGYGHT

>Sequence_836

KPAFNDGVKAALNPFAAFFA

>Sequence_837

KAALNPFAAFFAFLARIFGG

>Sequence_838

DASVPRPAPKPVPVIDTIPA

>Sequence_839

PGSNRGSAKFISAPIAAIAV

>Sequence_840

KFISAPIAAIAVTAVVGSAG

>Sequence_841

MSVQQSIVNWFVNHRGKLTY

>Sequence_842

RFGFGNPRSTNGLQGICIHT

>Sequence_843

IKKNSKKYAKLIAGAINGKP

>Sequence_844

AINGKPIGGVVASSKKPKPK

>Sequence_845

GFIQVTWADNYRAFGRWCHA

>Sequence_846

PKWAWLGAVWYFTTHRHGLV

>Sequence_847

RVWAHRIDTPGSCLLYTSPS

>Sequence_848

DLCSKTSGGYGLYSAQHKRL

>Sequence_849

ESASWGIGQVMGFHWKALGY

>Sequence_850

KDWKAFARGYNGPAYAKNSY

>Sequence_851

MGRQWPLPRGKFTITSRFAG

>Sequence_852

RGKFTITSRFAGRINPVTGQ

>Sequence_853

TPFYACAGGTVQYIGAASGY

>Sequence_854

AKYAVEKAKAYGVPAGSYIA

>Sequence_855

LRNHLSTSRIVKSFGTCLWV

>Sequence_856

NVYRGDGVKFAGRGPIQLTG

>Sequence_857

YYKKKVEVAPKPTPTKKGAT

>Sequence_858

DLPNWITTKYFGIALWGNAI

>Sequence_859

DDGVIGAGTLTAVYRKLGAS

>Sequence_860

LGASPVNADLLGNASNVFLR

>Sequence_861

RRLISLGYNPGGADGLIGPK

>Sequence_862

LNCNSGSIGISLCGMTGANE

>Sequence_863

AGRNAGVFVPVLNTAMNRYG

>Sequence_864

NRYGIVGTARAAAFVAQVGH

>Sequence_865

PVKLTPADLRAGKRGFCGHL

>Sequence_866

TNVLTPKYFTDFIKGFIGPL

>Sequence_867

MLRLLNQGLREQACRQFPRW

>Sequence_868

FPRWVYANGKVLNGLKTRRD

>Sequence_869

QLAFWGCVTLLAVASHSHAG

>Sequence_870

VLTGVGIAWHAGAGWFTNWP

>Sequence_871

WHAGAGWFTNWPTNAANQVS

>Sequence_872

GTSPWPPAMLDAYYRCCAAI

>Sequence_873

DKDGYMATSWKKIGEKWYYF

>Sequence_874

KKIGEKWYYFNRDGSMQTGW

>Sequence_875

CLSEGTIGWDAPPHPHNPGV

>Sequence_876

KTTAKKKTTAKKKMKKLSYI

>Sequence_877

SCPYAYDFQPGGWCDYTSVL

>Sequence_878

STTKKTTPKAATSKPIKKIS

>Sequence_879

KTVANKPGSASTPATKSNNG

>Sequence_880

STGPFTTCPFTGWLNPGVTI

>Sequence_881

PFTGWLNPGVTITYDECMVQ

>Sequence_882

TAQGTPGTSNYVLGPLWGTI

>Sequence_883

AQSANKTATKKKTKAKKKMK

>Sequence_884

QCFDLANQWWLYLFNHTLKG

>Sequence_885

YAKETTVNKVKSKSKPVAKA

>Sequence_886

KVKSKSKPVAKATKKKGKKI

>Sequence_887

MALRKKAGAAAICAVSAIIA

>Sequence_888

LELIGNAEACRRSPYICPAG

>Sequence_889

ACRRSPYICPAGVLTDGIGN

>Sequence_890

CQQFPRWIYGGGRVLPGLVA

>Sequence_891

CKHVLEPLRKRVGRVVVTSG

>Sequence_892

RKRVGRVVVTSGYRCGELNK

>Sequence_893

VTSGYRCGELNKAVGGARHS

>Sequence_894

ELNKAVGGARHSRHLTGRAA

>Sequence_895

SDTAMCRKYAAILRAHTPCT

>Sequence_896

IAVFNGSLGGGAGHVAVVTK

>Sequence_897

GWTNGVASPGWGPERVTRRW

>Sequence_898

DFPSKINAGKKAKQVIKNAV

>Sequence_899

MKRIPTNNGYWLLNPCVFFV

>Sequence_900

GYWLLNPCVFFVSALILIFS

>Sequence_901

VFFVSALILIFSLVSCKPAA

>Sequence_902

LIFSLVSCKPAAAETIPNAA

>Sequence_903

GAGIAWHAGRGWLDGWPTNN

>Sequence_904

GRGWLDGWPTNNANAVAIGF

>Sequence_905

ATAAILWFLGKRATKHTMIA

>Sequence_906

GTLDPKPAPPAPKPAPKPVA

>Sequence_907

PPAPKPAPKPVARPLTQQYF

>Sequence_908

QQYFTDFFGGYMGNVVSDVK

>Sequence_909

WGSHTDCGKGFPGDVFAASL

>Sequence_910

KGFPGDVFAASLTKYISNSA

>Sequence_911

PDKVGRWAEFANAHIYWTPQ

>Sequence_912

PQVSSKKYYKYNWSGVFTSN

>Sequence_913

IIKDLQANLWWIRFKYQAKG

>Sequence_914

LWWIRFKYQAKGSSNKDFYM

>Sequence_915

FNRKNIIPKIKKYLEKAGHN

>Sequence_916

ISSALDKTVGKIRGVTARND

>Sequence_917

WARFKFDNKGEYFYVALCRI

>Sequence_918

FPRWNKAKGKVLNGLTRRRA

>Sequence_919

KSGQYVAACKSLLKWKYVAK

>Sequence_920

CKSLLKWKYVAKRDCSIRSN

>Sequence_921

YVAKRDCSIRSNNCYGVWTR

>Sequence_922

YGLTHFIVPPVAKEFKVLKT

>Sequence_923

VLKTETKKAPTQTNKTKTKK

>Sequence_924

TKRITGWTMDKRGYNPHGVV

>Sequence_925

TIVNKPGSASTPATRTNSNG

>Sequence_926

FTPNTAIRTRYIGPFTSCPQ

>Sequence_927

PQVSENKYVKYNWRGTFTAH

>Sequence_928

VKYNWRGTFTAHKTNTLPIV

>Sequence_929

NPLKMWANRAVPESLKFRHD

>Sequence_930

MKRTLCALAAVAVSAAATVT

>Sequence_931

MKRNLRARKTVALTALAVCT

>Sequence_932

KTVALTALAVCTTLATTMGA

>Sequence_933

IRDRFINQKRWRDACYQLPR

>Sequence_934

KRWRDACYQLPRWVYVKGVF

>Sequence_935

QLPRWVYVKGVFNPGLDNRR

>Sequence_936

MANLKTKLSAAMLALIAAGA

>Sequence_937

SLTSYRDGAGIWTICRGATR

>Sequence_938

AGIWTICRGATRVDGRPVTQ

>Sequence_939

MRLKWAVAAAATVGFGLALP

>Sequence_940

YRYPKPQDSAPSGFRRASYL

>Sequence_941

ISAGLAVRWFPHIAAAMKEF

>Sequence_942

YAARSAAWFYTSRGCLRYPG

>Sequence_943

MGKVLIKRGTSAAGAIAIIL

>Sequence_944

GDMRDFPKHCYGKITVCGDK

>Sequence_945

LAFNVGTGNACGSTMVKFIN

>Sequence_946

NACGSTMVKFINQKRWRCLL

>Sequence_947

VWPAVERAAVLARDICKRNG

>Sequence_948

AVLARDICKRNGIPIRKLST

>Sequence_949

DPGPYFPWDKFIATVNGAKV

>Sequence_950

VDLLSWVKNPLTGKLYRTKD

>Sequence_951

ERVDLLSQVLNPVSGKLYKT

>Sequence_952

VLNPVSGKLYKTKDALWSMW

>Sequence_953

RAWRAGRAAAAIVTVPVGVL

>Sequence_954

AAAIVTVPVGVLKAGAVAFT

>Sequence_955

AALPGFNSALVQAACTTVKR

>Sequence_956

LYKGRGRIQLTGKGNYERFS

>Sequence_957

QLTGKGNYERFSVWCFVRKL

>Sequence_958

ERFSVWCFVRKLVPTRDYFV

>Sequence_959

MRRSRSLLYIAAACVGLLVC

>Sequence_960

YIAAACVGLLVCLAASPPAK

>Sequence_961

VMPSTATWLAELFPKVLGKV

>Sequence_962

PKGFHVTSPFGPRWGQFHFG

>Sequence_963

PKPRADGLRPNPGWRGDPTF

>Sequence_964

RPNPGWRGDPTFLPKVLRAF

>Sequence_965

IHLSRTGVATLCGAGIAYHA

>Sequence_966

GIRDRSPSRGLGMCIRDSTT

>Sequence_967

VSYIPYKDIVGVWTVCHGHT

>Sequence_968

IVGVWTVCHGHTGKDIMLGK

>Sequence_969

GDIKGACDQLRRWIYAGGKQ

>Sequence_970

QLRRWIYAGGKQWKGLMTRR

>Sequence_971

FTRLVESFNYSISGLAGFIR

>Sequence_972

GNKAAGDGWKYRGRGIIGIT

>Sequence_973

AARSAAWFFASKGCMKYTGD

>Sequence_974

VWTQLRGPGGKGWKQLGQDS

>Sequence_975

DVLRAFGVTVRERPGWKLWG

>Sequence_976

TVRERPGWKLWGNGDFGAIK

>Sequence_977

ATICGAGIAWHAGRGRYPGW

>Sequence_978

YRICAAILTKLGKRATTQTL

>Sequence_979

DVNIVKRITNFITAFVGPIG

>Sequence_980

RDKGQYPGWAQLGKNPRGGN

>Sequence_981

QRFQFGAPRSTSKIRNIFIH

>Sequence_982

YYGLTHFIVPPVASKSKPKK

>Sequence_983

KPKKEVKNTAPTQTKKSSKK

>Sequence_984

DKHITGWTMTKRGHNPKGVV

>Sequence_985

GDKPKSTTVKSKPASASTPA

>Sequence_986

NKPSTVPKKGWIAVYTSGIY

>Sequence_987

TSGTLTSGFGPRWGTMHNGI

>Sequence_988

SATAIDINAPKYPWGTYRMD

>Sequence_989

GYLGAYGKPAANPMKQPTPK

>Sequence_990

CFDLANQYWYYVTGHALRGL

>Sequence_991

VSKTKARMVKPKPIKNVKKK

>Sequence_992

VKPKPIKNVKKKKIMIVAGH

>Sequence_993

FHLDSAGPKATGGHTIIPAG

>Sequence_994

KKNLKPFTKSIASAINGKPI

>Sequence_995

KSIASAINGKPIGGASAGKV

>Sequence_996

GKPIGGASAGKVKSVKKTWD

>Sequence_997

AGKVKSVKKTWDWKGRFYPN

>Sequence_998

KTWDWKGRFYPNTTIKVRKK

>Sequence_999

VVQLYKDTKKKLWWGKFKYP

>Sequence_1000

KKKLWWGKFKYPTNPNAGYF

>Sequence_1001

AGVATMCGVGVAWHAGRGSY

>Sequence_1002

QLTGGRNRGQYPGWAQLGTK

>Sequence_1003

PYSYDCSSSVYFALTAGGFL

>Sequence_1004

QRGDVFIWGTRGASSGAFGH

>Sequence_1005

VTIYRPPFGATTKTATAALS

>Sequence_1006

GATTKTATAALSKGLSLTSK

>Sequence_1007

DFGPLLRKAYGRWVRNAKGK

>Sequence_1008

WGTLAQRMNPRASAGLFFNK

>Sequence_1009

FFNKMMTFNWRAMDPGAACQ

>Sequence_1010

SMANRWGPGSYDCSSSVYFA

>Sequence_1011

DVFIWGVRGASTGSAGHTGF

>Sequence_1012

DYNIGGGNFKNSSVVAYLNK

>Sequence_1013

KIYYGQCFDLANVYWAKLFG

>Sequence_1014

KPGDVVVFPRTFGGGYGHVA

>Sequence_1015

PRTFGGGYGHVAIVISATLN

>Sequence_1016

RRTHGYEFPMWFIRPFYKKA

>Sequence_1017

PMWFIRPFYKKANVTKSIQS

>Sequence_1018

SIQSPTVTKKKATPKKRKMK

>Sequence_1019

KKKATPKKRKMKTLKYIRDE

>Sequence_1020

GPNFPWDVFLQKVRDRLKPT

>Sequence_1021

QRFNFGGPRPTAKIRNIFIH

>Sequence_1022

MNWSAAPGYVTYRTCLLYTS

>Sequence_1023

CFDTANQYWMYLFGHMLKGI

>Sequence_1024

VKSKMTPKKKPKMNKGKKIL

>Sequence_1025

KDGYWFIRFKYQAPGSSNKH

>Sequence_1026

YDFPMWFIRPKYKKEGQTRS

>Sequence_1027

TKNSKKTKKVTRKAKKLNYF

>Sequence_1028

LNYFKDYVKGYNLPKRGYNP

>Sequence_1029

SSNTKSTVAGAWRKNSYGTW

>Sequence_1030

QLASADYGFYGAVWYWATRG

>Sequence_1031

FYGAVWYWATRGLNDFADAG

>Sequence_1032

TRGHGDFNVIWGVVAHHTGG

>Sequence_1033

GLAQRIAQSIAGVIGTPVRG

>Sequence_1034

YGHMWNAFQTGLKAGDRVKK

>Sequence_1035

AFVSFTYNLGGGAFCKSSLL

>Sequence_1036

AAADEFPKWNRGGGKVMKGL

>Sequence_1037

MKAILDFLKTLTGNQSTRKK

>Sequence_1038

QFLVWNRGGGKMLRGLVRRR

>Sequence_1039

MTTTFHRHWRDVPKNAWRWP

>Sequence_1040

DVPKNAWRWPNFSPAEIACR

>Sequence_1041

QLGVDGILGPASWAAAWTKP

>Sequence_1042

PKWNKAGGRVLAGLVKRRKA

>Sequence_1043

WNQSSMLSNLKAGRYKHACA

>Sequence_1044

NLKAGRYKHACASLLKYKYV

>Sequence_1045

MLTNLKAGQYIAACKSLLKW

>Sequence_1046

MSRPGSTLPVRSLVAALALS

>Sequence_1047

VDFLYQYGIGNWRASSMRRH

>Sequence_1048

GYDCSTRIDGRPNTVCWGVW

>Sequence_1049

DGRPNTVCWGVWERQQQRHA

>Sequence_1050

GPFRSCPVSGVLPVSYTHLR

>Sequence_1051

IKAKTTKPKPVKKVKKRKVM

>Sequence_1052

KPVKKVKKRKVMIVAGHGYN

>Sequence_1053

EEWEKIGGVWYYFDAKGYCL

>Sequence_1054

KKYVKYDWKGTFTAHKTNTL

>Sequence_1055

LKSTAAGRYQLLFRWWKPYR

>Sequence_1056

VYKRQTVAGAWKRNSYGTWY

>Sequence_1057

PSTVPKKGWIAVWSAGTYAE

>Sequence_1058

KKEAPKQKKSTEKKKIIPHI

>Sequence_1059

KKKQPQMTWNWKGRFTSNST

>Sequence_1060

KYPTNPSTGYFYCAVCRITD

>Sequence_1061

MARYVGRHRKHTTPAPVKAV

>Sequence_1062

IKGGVALPASGAFTSGFGSR

>Sequence_1063

ASGAFTSGFGSRWGSFHSGV

>Sequence_1064

NDGTSGWSAAQYNAYVRGCA

>Sequence_1065

RGCAAILNKLGQPSSHVIGH

>Sequence_1066

VVNQIDAQAKVATWLGKRLH

>Sequence_1067

AKVATWLGKRLHAGEKPCKD

>Sequence_1068

ALHKALKARKWATFAKGYNG

>Sequence_1069

RKWATFAKGYNGPDYARNLY

>Sequence_1070

LRWNKGGGKVMKGLVRRREA

>Sequence_1071

MKKTINKIGLKLIAQFEGCY

>Sequence_1072

LCPANVWTIGIGTTIYPNGV

>Sequence_1073

VPTIGFGTTSGVKIGDTTTP

>Sequence_1074

MNTKIRYGLSAAVLALIGAG

>Sequence_1075

GVRHNPYKDIVGVWTVCYGH

>Sequence_1076

VWTVCYGHTGKDIIPGKTYT

>Sequence_1077

KGACDQLRRWTYAGGNQWKG

>Sequence_1078

NFQREFLNKIKPGAIAGWHK

>Sequence_1079

KGWKIIPNERATVPKQGWIG

>Sequence_1080

WNSLAKSSFSGWCRPELAGL

>Sequence_1081

FSGWCRPELAGLPWLRCTRA

>Sequence_1082

NWYWFDNSGHMATGWKRIAE

>Sequence_1083

MNIPTSLLKALCTFIVGYCI

>Sequence_1084

RLRTDLPQVGYAPYRQVHAH

>Sequence_1085

TIFNGISGSRPTEKPKYYIM

>Sequence_1086

QEGWKRNSTGWWYVNADCSY

>Sequence_1087

WYYFKETGVMATGWVKYKDH

>Sequence_1088

NRVTYSMTYRNGPGSFDCSS

>Sequence_1089

AGVEAGMPKLPWPCSTESMH

>Sequence_1090

SAEGLLKTFPKYFNKDQAAA

>Sequence_1091

FPKYFNKDQAAAYARNQQKI

>Sequence_1092

QAAAYARNQQKIANRVYANR

>Sequence_1093

AFRGRGPIQLTGKHNYKAFG

>Sequence_1094

LVSEPVLGCLSAGWYWKANS

>Sequence_1095

AGSKKVTWNWKGRFTANTTI

>Sequence_1096

NWKGRFTANTTIKVRRKPGL

>Sequence_1097

FRLRGNAKDAINNKLPSGWK

>Sequence_1098

DFSNMAWHCANSYGNANYAS

>Sequence_1099

MRLKQGLSAIVAVGTVVSGI

>Sequence_1100

LHVYKLHTGLDFAAPCGTPV

>Sequence_1101

GLDFAAPCGTPVGAAADGVV

>Sequence_1102

GTPVGAAADGVVSFVGWAGG

>Sequence_1103

SGIGAGVATAQTKPAPAPTT

>Sequence_1104

LDQWNNTVSKYFKGVASIIG

>Sequence_1105

SKYFKGVASIIGRQRIGVYG

>Sequence_1106

SIIGRQRIGVYGSSRVISWA

>Sequence_1107

WSPRFNFGSPRNTSNIIGVC

>Sequence_1108

STGNKGNDVLLHVSFVCRAA

>Sequence_1109

VLLHVSFVCRAAWTREKWLA

>Sequence_1110

KWLAYPKMLRGGATVVAHWC

>Sequence_1111

LRGGATVVAHWCKKYNIPPH

>Sequence_1112

AHWCKKYNIPPHKVTAAGLP

>Sequence_1113

RRFNFGGPRSTSNIRNIIIH

>Sequence_1114

QLVREYAGQAPKPAPRPAPK

>Sequence_1115

QAPKPAPRPAPKTGGNMNTH

>Sequence_1116

MNTHRDIVKPINKFTGDFIK

>Sequence_1117

KPINKFTGDFIKGFCGPIYD

>Sequence_1118

DFIKGFCGPIYDRANEAVLL

>Sequence_1119

HVGLKVDGIAGPATRAAIHA

>Sequence_1120

IAGPATRAAIHAELLKRPLV

>Sequence_1121

AIHAELLKRPLVTFGTARPT

>Sequence_1122

RPLVTFGTARPTPKPVPVID

>Sequence_1123

ARPTPKPVPVIDTIPPAASS

>Sequence_1124

KYSAYMAGLRKRLLWRFIAE

>Sequence_1125

LRKRLLWRFIAELIFGASVF

>Sequence_1126

ASVFIVLVFFFIASAPSCAQ

>Sequence_1127

ICQRWAFTLSAYNGGLGWVN

>Sequence_1128

RHQPLYVAAGWGTSVCGVGG

>Sequence_1129

GYRYRGRGFIQLTGRHNYQR

>Sequence_1130

PRRISPAGIDLIKRWEGCRL

>Sequence_1131

IDLIKRWEGCRLTAYRCPAG

>Sequence_1132

GCRLTAYRCPAGVWTVGYGS

>Sequence_1133

CPAGVWTVGYGSTGDHVHDG

>Sequence_1134

RRDLERFERAVAGACPASAP

>Sequence_1135

AGDFDGAAKQFGRWVFAGGK

>Sequence_1136

KQFGRWVFAGGKRLPGLVNR

>Sequence_1137

LTGRANYRAFTAWTKRQGLS

>Sequence_1138

TNGTNWIGSFPKVLNDFIGG

>Sequence_1139

FNNAMRTAGITTVKRAAMWC

>Sequence_1140

IGLIAPVLNKYMPGAGYKVR

>Sequence_1141

LFERHWMYKLLKQAGKPTPI

>Sequence_1142

KLLKQAGKPTPISDVCNPKA

>Sequence_1143

PTPISDVCNPKAGGYKGGAA

>Sequence_1144

SWGLFQIMGFHWKSLKYGSL

>Sequence_1145

GAVSAGGVNFATKWLDYVKS

>Sequence_1146

NFATKWLDYVKSKTGVTPMF

>Sequence_1147

PRELYGSLKYWSKMTCFQYT

>Sequence_1148

KYWSKMTCFQYTSSGRLSGY

>Sequence_1149

YNVLGMFKVNRQGGAKLYTS

>Sequence_1150

VFFPKIGWVGANEIIHQGGH

>Sequence_1151

TSGYRPPTVNAAVGGASNSQ

>Sequence_1152

SSFTHIDLRGYRARWAYGNA

>Sequence_1153

CPHRSAKLHSGIDPLKQGWN

>Sequence_1154

KPTSTTTTKKVASASTPATR

>Sequence_1155

KKVASASTPATRKTINGWKT

>Sequence_1156

PATRKTINGWKTNKYGTLYK

>Sequence_1157

GWKTNKYGTLYKAKKGSFTA

>Sequence_1158

TLYKAKKGSFTANTAIITRY

>Sequence_1159

RPSTVPQKGWIVVLTAGTYS

>Sequence_1160

NQGDQKGACDQLRRWTYAKG

>Sequence_1161

CDQLRRWTYAKGKQWKGLVT

>Sequence_1162

IEDRITRWNRCLSIGAALLP

>Sequence_1163

VEGQVRWDWPLYGRIAEVFK

>Sequence_1164

WPLYGRIAEVFKAAAVDLKT

>Sequence_1165

TPIITRTVGKLRSCPHPPLL

>Sequence_1166

GKLRSCPHPPLLPAGATSVY

>Sequence_1167

LPIRTWNGVAPGNAGYAVGP

>Sequence_1168

DGDGIKYAGRGYVMITGYAN

>Sequence_1169

SVFELITRRINGGLNGLKDR

>Sequence_1170

GWKPIDSNPKKVTKKVNTPS

>Sequence_1171

WRDWGMGDFHNIWGVAVHHT

>Sequence_1172

IVPIIGGKISWNLMIYPLPK

>Sequence_1173

GQNWNGKGWTNGVAQPGWGP

>Sequence_1174

FPNNLSVGNKAKGIIKQATT

>Sequence_1175

TNERDFIRKYITPNIAKYLR

>Sequence_1176

KYITPNIAKYLRHAGHEVAL

>Sequence_1177

HGKPIGGLVAGNVKTSAKNQ

>Sequence_1178

VAGNVKTSAKNQKNPPVPAG

>Sequence_1179

KNMLPSIWRKLTSKLGKEKK

>Sequence_1180

WRGDPTFLPDVFRAFGVNFR

>Sequence_1181

DSSGYFQQRPPWWGTPAQRM

>Sequence_1182

LKLVSYLCPANVWTIGFGTT

>Sequence_1183

PANVWTIGFGTTVYPSGKKV

>Sequence_1184

FGTTVYPSGKKVKQGEKCTP

>Sequence_1185

AADQFPLWNKGGGKVLKGLV

>Sequence_1186

IGRDKKNSNIKGFVYTFNKY

>Sequence_1187

ILFKGRGPIQTTGRKNYDVA

>Sequence_1188

PVRPGVPSENGWPMCNRGGC

>Sequence_1189

ENGWPMCNRGGCILITAPGA

>Sequence_1190

RGGCILITAPGAGRTVPVRA

>Sequence_1191

PWGRRVMPAARIAKVRALLA

>Sequence_1192

RDGVYTFVGAGIAWHAGTGS

>Sequence_1193

GVAAILNRLGQTASHVIGHK

>Sequence_1194

LITPDGKCHLIAVGPCNHAG

>Sequence_1195

HLIAVGPCNHAGIGKYPGIA

>Sequence_1196

QYPNSTWKIYANTPSFVPKP

>Sequence_1197

IYANTPSFVPKPGDVVCWTY

>Sequence_1198

VPKPGDVVCWTYGAYGHTAI

>Sequence_1199

KETTKNKIKSKTKPVKKAKA

>Sequence_1200

KDGYWWVRFRYVQPGSSKKD

>Sequence_1201

FRYVQPGSSKKDFYCAVCKI

>Sequence_1202

FPMWFIRPLFKSENNTKKSN

>Sequence_1203

LKKWKLPANRNTVRLHMEYV

>Sequence_1204

TGWDPVKQGRPNQATINKLK

>Sequence_1205

FVRPNFKAAEKITWNWSGRF

>Sequence_1206

YYDNPMYFIRPLYKAKTTVV

>Sequence_1207

IRPLYKAKTTVVDKVKDKVT

>Sequence_1208

TTVVDKVKDKVTSVAKPTTK

>Sequence_1209

SVAKPTTKGKKILIAAGHGY

>Sequence_1210

NGKPINGAPASKKKVTWNWK

>Sequence_1211

PASKKKVTWNWKGRFYPNVG

>Sequence_1212

WNWKGRFYPNVGKTGIKVRR

>Sequence_1213

PNVGKTGIKVRRKPGLQGAI

>Sequence_1214

KYPTNPRAGYFYLAVCKIKD

>Sequence_1215

GGNSAAEDLAKYLGNAANQV

>Sequence_1216

SANPRSINLCFAGSRASWSR

>Sequence_1217

GDQFPWDYFANRVAFWAAGG

>Sequence_1218

AAGGVDGPTQPPTPAKPQPV

>Sequence_1219

GGFAVDLVPIVNGKVSWDWR

>Sequence_1220

PIVNGKVSWDWRYFYAIAEA

>Sequence_1221

RWGGCWEVINNKSGTAKSWV

>Sequence_1222

FVRWSNANGKWMLGLFRRRL

>Sequence_1223

GKWMLGLFRRRLSEAMIYAG

>Sequence_1224

WLRFKYQAQVQVKKISTAPF

>Sequence_1225

PNNTFPKGWKVVKNYPSYVP

>Sequence_1226

KKKAPDKWASLIKKASKEMG

>Sequence_1227

TWKNDYNPNGGWSPRGAKVK

>Sequence_1228

NGGWSPRGAKVKAGGGGRDF

>Sequence_1229

TFIVGWADNRLGNVPPARCA

>Sequence_1230

NGYRWITYIANNGQRCYIAT

>Sequence_1231

ISSFGNFSALWKKVCKVSYE

>Sequence_1232

KGIVKSFNLPKKSSAVYHTV

>Sequence_1233

AYGGNPRGKPRDIYPGDHHR

>Sequence_1234

WNHPLGNQKPAMWQFGSNAL

>Sequence_1235

RTLCALAAVAASATATVAAP

>Sequence_1236

KVDLFKAVTGLLTSTAGALN

>Sequence_1237

ACGYSRNVKNLDKQIAKYIN

>Sequence_1238

KNLDKQIAKYINGVKKSAPS

>Sequence_1239

PPICAIAIYTKGVYSRWGHT

>Sequence_1240

ADDGTWQRSSPYGPRGGGHH

>Sequence_1241

ADRAPGSVSGFGNWIWGDAQ

>Sequence_1242

GWKVNKYGTYYKSDVAHFTP

>Sequence_1243

WFIRPKFSQKSSTKKASLFS

>Sequence_1244

STWKVYGLGWARRAMGTLAA

>Sequence_1245

MKTKKQALKWILNTIGQGID

>Sequence_1246

GNAIDAPKNNFKGTAKVIKN

>Sequence_1247

GVSHFIRPKFKKTAKTEVTS

>Sequence_1248

GLKNAGYSRYAQGVAHAYAS

>Sequence_1249

KNSGSLPKKQGKKQTSKSNI

>Sequence_1250

EVGRFTVKVAGLNVRKAPHL

>Sequence_1251

ANRRDMNGWKINKYGTCLLY

>Sequence_1252

WGNTDHTDPGSGFPICLLYT

>Sequence_1253

TCGVRQGIVTRTTGPFTSCP

>Sequence_1254

QALRDRNWQRFAYYYNGPAY

>Sequence_1255

VQGWNTRSLGICLAGGVSEK

>Sequence_1256

PTIHSVIGHRDTGAKKACPS

>Sequence_1257

SLSKLEGVDPRLVKVIKRAI

>Sequence_1258

TVKAVCARRLSFLRGLSTFS

>Sequence_1259

RLSFLRGLSTFSTFGKGWSR

>Sequence_1260

STFSTFGKGWSRRVADVEAK

>Sequence_1261

LGNTHAGDGWKFRGRGLKQL

>Sequence_1262

GWKFRGRGLKQLTGRDNYRR

>Sequence_1263

RRINGGLNGYADRQARYARA

>Sequence_1264

VRFADAARPVPKPNPAPARG

>Sequence_1265

PVPKPNPAPARGTPAGGGVI

>Sequence_1266

PARGTPAGGGVIAVVVAAIA

>Sequence_1267

GGVIAVVVAAIAAVAAFIGL

>Sequence_1268

ALGFYTGQIDGLWGPLSHGA

>Sequence_1269

IDGLWGPLSHGAFTNARRSV

>Sequence_1270

MNRARTWVGALAMSLAAFAT

>Sequence_1271

DFSGQYGSANWRKSSMRRHL

>Sequence_1272

YAKACDALLMWRKAGGYDCS

>Sequence_1273

YDCSTLVNGKPNRRCWGVWV

>Sequence_1274

GKPNRRCWGVWVRQLERHAQ

>Sequence_1275

VRLWGNAKDAIKNDMKGLAT

>Sequence_1276

HYYDGVTHFIRPHFRKESKP

>Sequence_1277

FIRPHFRKESKPKNVYKWNG

>Sequence_1278

ESKPKNVYKWNGKFTSFKTN

>Sequence_1279

KWNGKFTSFKTNKRPIRVRL

>Sequence_1280

KDGFWWLGFYYQQKGASKNR

>Sequence_1281

FYYQQKGASKNRFYMSIGKI

>Sequence_1282

MTPSMKKKLIGVIAGGGGAI

>Sequence_1283

GRYQQLYLFWPHYKKQLALP

>Sequence_1284

ESFNYSITGLAGFVRAGRLT

>Sequence_1285

RNGNKGPGDGWLYRGRGLIQ

>Sequence_1286

QDENAARSAAWFCATKGCLK

>Sequence_1287

AAWFCATKGCLKYSGDLVRV

>Sequence_1288

QPVYDAVVSWAFNVGTYAAC

>Sequence_1289

SWAFNVGTYAACRSTLGAYI

>Sequence_1290

WRSACLQLPRWVFVKGVFSQ

>Sequence_1291

PRWVFVKGVFSQGLQNRRDR

>Sequence_1292

MNLQTVKRCTVGVVLAVAAT

>Sequence_1293

VSFAFNVGTGNACGSTLVKL

>Sequence_1294

LVKLLNQRRWADACRQLPRW

>Sequence_1295

RWADACRQLPRWVYVNGVFN

>Sequence_1296

YVNRNECLWYHPTDYVEWHC

>Sequence_1297

FGTSCPHRSWDIHVGKGAAN

>Sequence_1298

VFNGGYGGGAGHVAPVTRAT

>Sequence_1299

IRFEFPSNINAGKKAKKIIK

>Sequence_1300

INAGKKAKKIIKNAVSKNEK

>Sequence_1301

KDFINFKWPKGFQVIKGKNQ

>Sequence_1302

RSGKLKGVVIHNTAGSATAK

>Sequence_1303

GLPVNRSTVRLHCEFVPTAC

>Sequence_1304

VRLHCEFVPTACPHRSMTIH

>Sequence_1305

PTACPHRSMTIHTGWNPVTK

>Sequence_1306

MTIHTGWNPVTKGAAPSNIV

>Sequence_1307

KTKTAVSKKIGNGWKKNKYG

>Sequence_1308

KIGNGWKKNKYGILWKKEKG

>Sequence_1309

RYKGPSIHNPIAGGLEYNQS

>Sequence_1310

RYQLLTIGTRRPGRQLRTGK

>Sequence_1311

KFKYPTNPGTGYFYLPVCEI

>Sequence_1312

RPHYKAKVSTISKVKAAVTK

>Sequence_1313

STISKVKAAVTKPKKTKSTG

>Sequence_1314

AVTKPKKTKSTGKKILIVSG

>Sequence_1315

IGGTSAGNKKAPQTRWNWKG

>Sequence_1316

KKAPQTRWNWKGRFTPNTAI

>Sequence_1317

YWWISYTSKGKTYYSAVCKI

>Sequence_1318

FTNGNQPIIARTTGPFRSCP

>Sequence_1319

IARTTGPFRSCPIGYTFQPG

>Sequence_1320

RSCPIGYTFQPGGYTPTYKE

>Sequence_1321

TWNGVAPPSHSVGKLWGTIK

>Sequence_1322

INGKPISATSGGSKKSLGTG

>Sequence_1323

ETVMLHCPFVSTACPHRTMA

>Sequence_1324

VWIGFYWNNTRYYMPMRTWN

>Sequence_1325

KLLWPTSATWITSPYGWRTH

>Sequence_1326

TWITSPYGWRTHPIFGTPRF

>Sequence_1327

TPRFHSGVDIAGPFGTPIYA

>Sequence_1328

TVYAHLGGHFVSTGQRVSRG

>Sequence_1329

IVHSILKKTNRNIILAVFFL

>Sequence_1330

GQWWWADARTCMDPTLSAGL

>Sequence_1331

YGDWKNIGHGDFGGIWGVVA

>Sequence_1332

HGDFGGIWGVVAHHTGGNSS

>Sequence_1333

AGVAYVVGAGIAWHAGNGSW

>Sequence_1334

AGIAWHAGNGSWPGIAANNA

>Sequence_1335

WSPAQYWAYVKCCAAICRRL

>Sequence_1336

YVKCCAAICRRLGVRADRVI

>Sequence_1337

KQGTYQLTSPFGPRGGTMHQ

>Sequence_1338

FGQWIVLDHNIGGKVYSTVY

>Sequence_1339

HNIGGKVYSTVYGHMFPDDL

>Sequence_1340

IGQFPKVLNEYMPAAKYKHR

>Sequence_1341

LNCRCGCGMTITKPLLDKLN

>Sequence_1342

LAGVPFAVTSGARCKEHNRK

>Sequence_1343

INNKLPKGWKLVLNRPSTVP

>Sequence_1344

GNKPAPVRAATAVAANAIAK

>Sequence_1345

ASRAGRAVSTAEYFASTSRS

>Sequence_1346

RLYGQGRTAAQCIAKGVPAG

>Sequence_1347

AAQCIAKGVPAGYAKPAERK

>Sequence_1348

ENGKLGLWPKLAQAFKQAAR

>Sequence_1349

AEARPMDYACVRPLFGGRMT

>Sequence_1350

DGWAYRGRGYVQITGRANYA

>Sequence_1351

DIAAWILVSGCVKGWFTGRR

>Sequence_1352

PGNYMKPLYITVHNTANTAK

>Sequence_1353

GIPLANVVPHKRWSGKQCPR

>Sequence_1354

PHKRWSGKQCPRKLLNRWDS

>Sequence_1355

IAIAHTNKKPTVKPAKTTSK

>Sequence_1356

KPTVKPAKTTSKPAASKPAK

>Sequence_1357

TTSKPAASKPAKKTHNLPSG

>Sequence_1358

KVTKPLTKGAGVKALQKALA

>Sequence_1359

KIAKKTKAILEKVYGATVKL

>Sequence_1360

GKQQKVVHDAIYRKIKGKAV

>Sequence_1361

KVSSSNKTKPTNKPSPKGVK

>Sequence_1362

KPTNKPSPKGVKMAVVKPNA

>Sequence_1363

HSNLYDWGQCTWHVFNKRAQ

>Sequence_1364

FPKNVSVAKKAKRKLTSKKV

>Sequence_1365

QGRKWADFARRYNGPAFKDN

>Sequence_1366

WHTDPEPHGRGWRDVGYHYV

>Sequence_1367

GGYNTGNVGICLVGGVAADA

>Sequence_1368

AVIMGHNGFPGHESRGCPCF

>Sequence_1369

FPGHESRGCPCFDWRAYRDA

>Sequence_1370

KDVKVKATPTQVTTGATPYT

>Sequence_1371

FVSVTKRNGYWWAKFKYPTN

>Sequence_1372

DFDKWYGRQCADLSTAYCYY

>Sequence_1373

IALTWQWLPDTFTRIKNVAS

>Sequence_1374

SPPAYVKHNYSGVWGFIRPK

>Sequence_1375

NYSGVWGFIRPKFATASKAK

>Sequence_1376

TGNVGNYKWNNTSMTYGAGT

>Sequence_1377

RITSIDVKPTIKGGAKKKTS

>Sequence_1378

KPGDVVVWNRKYGGGYGPVS

>Sequence_1379

PVKGTPAPRGPPSSAVPPPP

>Sequence_1380

RGPPSSAVPPPPAPEAGGKM

>Sequence_1381

VVGNVPPSGGCHLHLAVNKN

>Sequence_1382

FIHVNTVAPFGTFRTKIRKW

>Sequence_1383

PPEAYITSKFGPRWGTVHRG

>Sequence_1384

WLAGALTPGTKAGGTKPHGM

>Sequence_1385

RQWNYPLGNQRPAVWQFGSN

>Sequence_1386

KDDKKLIKHEKWHGVTQTAC

>Sequence_1387

SYIGNSGYRRYVAYRRLTGN

>Sequence_1388

TIDQLKHLFTGTRATPPAPR

>Sequence_1389

FTGTRATPPAPRPERERKPM

>Sequence_1390

YNCGPASAQTIIRAATGVLL

>Sequence_1391

TLNTPCKSHVPGSKHIAPLA

>Sequence_1392

WGTDFGLAGGSGGKPVYAVK

>Sequence_1393

LNWTQRFTFGKPRPTHHIKT

>Sequence_1394

FGKPRPTHHIKTIIIHVTVN

>Sequence_1395

KGKKILLVAGHGKGACQSYQ

>Sequence_1396

AGHGKGACQSYQPRGISAAG

>Sequence_1397

PVFAVKSGRVTRSGPASGFG

>Sequence_1398

CGVGVAYHAGRGYKTGWPTN

>Sequence_1399

FNDIVKPINKYTGDFVKGFC

>Sequence_1400

GGGQGSRDRGRGGTCNHLSI

>Sequence_1401

RPFAPGPWPINEKQVDALVS

>Sequence_1402

ALVSLLARLCRQHGIAVTRQ

>Sequence_1403

IAGKMTVDAVQKALAKGICT

>Sequence_1404

SQTSLDKLKGVNPALVKVVK

>Sequence_1405

KGVNPALVKVVKRAIQISKQ

>Sequence_1406

NLPINRDTVNLHRKYFSTSC

>Sequence_1407

VNLHRKYFSTSCPHRSWDIH

>Sequence_1408

KTDWVKFAQVIKKDGYWWHH

>Sequence_1409

GYWWHHFKYQAPGSSNKHFY

>Sequence_1410

VFNIGGGNFRKSTLLRKLNV

>Sequence_1411

WKYAGGKPILLSRRLREQAV

>Sequence_1412

YAKDIPFKNNFSGLATCLLY

>Sequence_1413

GKRQKQSKNSKKPRPKKQKS

>Sequence_1414

NSKKPRPKKQKSKPQKPKTE

>Sequence_1415

MWFIRPKYKDEKKTANYSAK

>Sequence_1416

MKSIRTKLIAAFVAAGLSAP

>Sequence_1417

EACQQLSRWVKAKGKTLRGL

>Sequence_1418

IPISNVRTHKSWSGKHCPHR

>Sequence_1419

GPNGNYKGNVDGSYPYGIFA

>Sequence_1420

SDFVWIPRYGGNKPAYPCDI

>Sequence_1421

ETGNVPGIGKCDLNQLIGSK

>Sequence_1422

GDQTGREIWACNYYNYPWNC

>Sequence_1423

WACNYYNYPWNCVLRYVGNN

>Sequence_1424

PWNCVLRYVGNNKSTNKHKQ

>Sequence_1425

TSPSIKSPIVACYGRNQPIH

>Sequence_1426

RWISYIGVNSGQRRYVACRR

>Sequence_1427

NSGQRRYVACRRLSGDARAW

>Sequence_1428

DGNVFDLVNYYWNYLFGHGL

>Sequence_1429

NYYWNYLFGHGLKGSGAKDI

>Sequence_1430

YGSHWGQGWGHTGIVLSATL

>Sequence_1431

KRTHHYSNPMWFIRPKYKVK

>Sequence_1432

PMWFIRPKYKVKTTAKQKTK

>Sequence_1433

YKVKTTAKQKTKAVVAKVTP

>Sequence_1434

IKKNLTKFNKAIANAINGKA

>Sequence_1435

NKAIANAINGKAIGGAPASK

>Sequence_1436

NGKAIGGAPASKPKPKPKAK

>Sequence_1437

PASKPKPKPKAKQTVWNWKG

>Sequence_1438

PKAKQTVWNWKGRFTANTTI

>Sequence_1439

GKFKYPTNPGAGYFWMALTP

>Sequence_1440

FIKQIRTYQAGKIPTATVAK

>Sequence_1441

PFRSCPYAYDFQPGGYTPTY

>Sequence_1442

YWAKLFGHGLRGTGAADIPF

>Sequence_1443

HNYEFPMWFIRPFYKKTNVA

>Sequence_1444

ESRIGWHTANAIGNKNGYGI

>Sequence_1445

MLQDGHVWIGYTWKSKRYYL

>Sequence_1446

YTWKSKRYYLPIRTWNGVAP

>Sequence_1447

YLPIRTWNGVAPPHHGVGTL

>Sequence_1448

MRIPARRTLLAAPALAAALV

>Sequence_1449

LLAAPALAAALVAAGGSVAS

>Sequence_1450

SVASAAEGTCPTPLKPLRAP

>Sequence_1451

TCPTPLKPLRAPALGTVTLR

>Sequence_1452

GSAVRSLQNLLNHAKRLRTS

>Sequence_1453

QCLSEGTIGWHAPPNPCLLY

>Sequence_1454

GWHAPPNPCLLYTSPSPRDL

>Sequence_1455

HAIDPMPWLRGATQPSGTGP

>Sequence_1456

GTGPNIRNVAAVTPGVGCGD

>Sequence_1457

VPAAFEPWIIKAGKTQGGLK

>Sequence_1458

MTNRIAKSAAAVAAAVSVAG

>Sequence_1459

KPYQDIVGVWTVCYGSTGAH

>Sequence_1460

VWTVCYGSTGAHVRSGGVRT

>Sequence_1461

PKWSYAGGKQVRGLLNRRLA

>Sequence_1462

RTTREYGGPRTRYAPYYGRG

>Sequence_1463

QVTWRDNYAKFGAWCKARGL

>Sequence_1464

AKFGAWCKARGLVPDADYFV

>Sequence_1465

APAAKPVSADRKTWLRKKIA

>Sequence_1466

ADRKTWLRKKIATHGIWSPA

>Sequence_1467

AVLCRRLCDKYGIPKRKLSA

>Sequence_1468

RDRGHGDFGTIWGVVCHHTG

>Sequence_1469

YLGRDGAYTVCGAGIAWHAG

>Sequence_1470

WHAGAGSWPGITNGNANQVT

>Sequence_1471

AILRHLRLPASRAIAHREWA

>Sequence_1472

GHGDFGSIWGVICHHTGSFG

>Sequence_1473

DQLVKGWPQLGKNAKGDPLT

>Sequence_1474

WGTDFGWPGGSANKPVFACQ

>Sequence_1475

GGSANKPVFACQGGTVTMVG

>Sequence_1476

FACQGGTVTMVGTASGFGMW

>Sequence_1477

HALPLDRYRRLLPAVTCALL

>Sequence_1478

EGRADLGNVFPGDGPRFKGH

>Sequence_1479

VFPGDGPRFKGHGPIQITGR

>Sequence_1480

VAGPGSKDQRGGLRPTGWPQ

>Sequence_1481

QRGGLRPTGWPQLGGRSLVD

>Sequence_1482

QTSGPGWSWDRTQYRGRGPI

>Sequence_1483

RGPIQLTWQANYRKFGEWCK

>Sequence_1484

QANYRKFGEWCKAKGYVTDS

>Sequence_1485

ASWYWLHGGPRPGRINAFAD

>Sequence_1486

NGMPDRRDRWNRCLALGSAL

>Sequence_1487

PNPSSPRGGAPVLWFLLHTQ

>Sequence_1488

FAGSRASWTRQQWFYNMRRG

>Sequence_1489

NKAGQRRTLRAGLAALLRGG

>Sequence_1490

GEPLTLVDGVAKAIGLLGKI

>Sequence_1491

ALLKALKGKKWAAFAKAYNG

>Sequence_1492

KKWAAFAKAYNGPAYARNLY

>Sequence_1493

AGAKAGVFVPALNAAMSKYG

>Sequence_1494

GSRKKIIIWVVALILVPVVI

>Sequence_1495

KSQWKNIKGEWYYFDNRGYC

>Sequence_1496

GEWYYFDNRGYCFINKWFND

>Sequence_1497

MVTGWMHIDHRWYYFKSDGR

>Sequence_1498

VWTIGYGTIKYPNGVRVKKG

>Sequence_1499

GAADQFLVWNKGGGRVLKGL

>Sequence_1500

WNKGGGRVLKGLVIRREAER

>Sequence_1501

GFWYVRPNGTYPKGQFEYIE

>Sequence_1502

ATSWKRIGGTWYFFNRDGSM

>Sequence_1503

GTWYFFNRDGSMQTGWIKYY

>Sequence_1504

IGYGTTKYPNGIRVKKGDTC

>Sequence_1505

DQFGLWVNARGKRLQGLVNR

>Sequence_1506

FDYANYGWNKLFGYNLAGNG

>Sequence_1507

GWGHVAWVVSATLNQIVVIE

>Sequence_1508

TWNWSGVFTANSTIKVRVKP

>Sequence_1509

IDKNGKRTSSYGKFSKVWYN

>Sequence_1510

SSYGKFSKVWYNQQTLTYLF

>Sequence_1511

TYLFSHILWGKFSTCPSFFM

>Sequence_1512

DYKMNLKTLLLTKNACYIAG

>Sequence_1513

LSGVLFCNDCGCKLYQLRKT

>Sequence_1514

RQEYYICSTFRKRGECTSHY

>Sequence_1515

FGIKKNGWPGKTYIKAATEQ

>Sequence_1516

MKKKFLVGAIVALFLLPIFP

>Sequence_1517

MRRIKQAGYTPMYYSYKPFT
