# Supplementary material for: Exploration of Human Skin Phageome to Reveal Endolysins and Novel Antimicrobial Peptides for Therapeutic Applications
Source: Microbiologyopen. 2025 Nov 9;14(6):e70115. doi: 10.1002/mbo3.70115 (PMC12597775; doi:10.1002/mbo3.70115)
Supplement: Supplementary file 3 — Figure S3: Endolysin Biochemical Characterization. A) 6% of endolysins have signal peptides, a feature crucial for targeting extracellular activity, enhancing their ability to interact with bacterial cell walls effectively. B) Shows the GRAVY index, where hydrophobic proteins (positive values) are associated with membranes, and hydrophilic proteins (negative values) are soluble. This is critical for understanding protein localization and interactions, which influence endolysin design for therapeutic applications. C) Illustrates stability metrics, with most endolysins being stable (Instability Index < 50) and thermostable (Aliphatic Index > 70). This stability is vital for their functionality in diverse environments. D) 94% of endolysins are soluble proteins and 6% are membrane‐bound. This distinction is significant because soluble proteins are often involved in enzymatic activity, while membrane proteins may play roles in interactions with bacterial cell walls or membranes. E) Indicates transmembrane helix predictions, where most endolysins lack helices, suggesting they are primarily soluble and not embedded in membranes. This supports their role in extracellular or cytoplasmic activity. [file MBO3-14-e70115-s001.pdf]

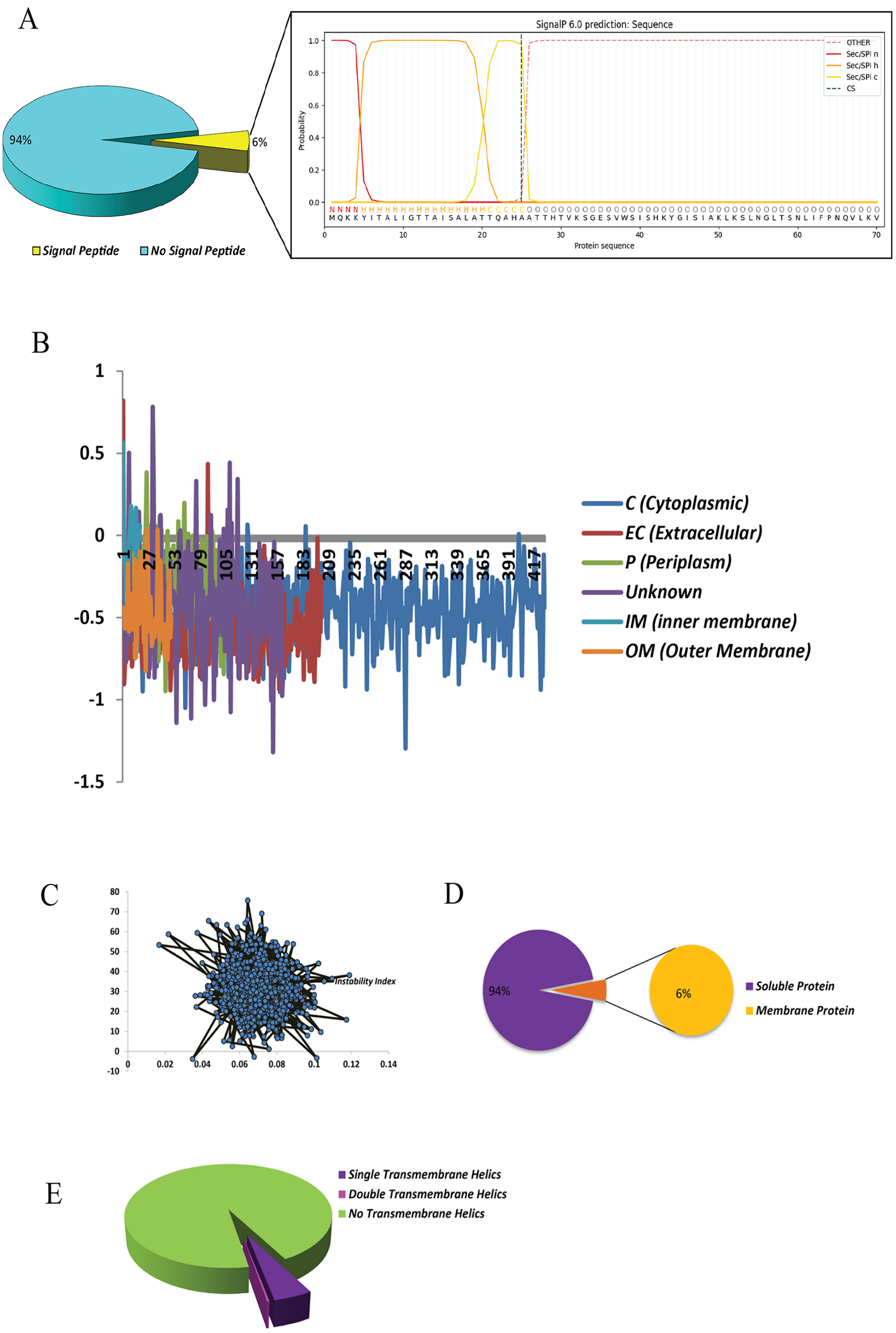
A
94%
6%
Signal Peptide
No Signal Peptide
SignalP 6.0 prediction: Sequence
Probability
Protein sequence
OTHER
Sec/SPI n
Sec/SPI h
Sec/SPI c
CS
B
C (Cytoplasmic)
EC (Extracellular)
P (Periplasm)
Unknown
IM (inner membrane)
OM (Outer Membrane)
C
Instability Index
D
94%
6%
Soluble Protein
Membrane Protein
E
Single Transmembrane Helics
Double Transmembrane Helics
No Transmembrane Helics
